# Supplementary material for: CAR design equips diverse immune cells with unique antigen-specific activation identities
Source: Mol Ther Oncol. 2026 Jul 29;34(3):201312. doi: 10.1016/j.omton.2026.201312 (PMC13521014; doi:10.1016/j.omton.2026.201312)
Supplement: Supplementary file 1 [file mmc1.pdf]

OMTON, Volume *34*

Supplemental information

CAR design equips diverse immune cells with unique antigen-specific activation identities

Ayano Sugiyama-Finnis, Takaharu Kimura, Kiyoko Izawa, Yuko Kawano, Takao Yogo, Hyojung Jeon, Tsuyoshi Fukushima, Ryoji Ito, and Satoshi Yamazaki

Table S1: Individual values underlying Figure S1e. Individual values for lineage distributions in peripheral blood at 15 weeks post-transplantation, expressed as the percentage of T cells, NK cells, B cells and myeloid cells among live, single hCD45+GFP+ cells for each CAR design. GFP expression denotes CAR-transduced cells.

Tissue	Condition	T cell (CD3+)					NK cell (CD56+)					B cell (CD19+)					Myeloid cell (CD33+)				
Peripheral blood	4-1BB/ζ	16.9	13.2	7.69	17.7	2.04	0	0	0	0.63	0	63.7	61.8	75.2	57.4	73.5	6.2	6.76	8.97	9.68	5.44
	2B4/ζ	0	35.2	13.2	0	32.1	0	0	0	0	0	20	40.2	57.9	68	53.2	20	11.5	13.2	0	2.63
	CD3ζ	8.75	0	13.6	1.34	0.91	0.19	0	0	0	0	57.2	70	51.3	80.8	80.8	26	12.5	14.3	2.23	4.11

Table S2: Individual values underlying Figure 1f and Figure 1g. Individual values for lineage distributions in spleen and bone marrow shown as the percentage of T cells, NK cells, B cells and myeloid cells among live, single hCD45+GFP+ (CAR+) and hCD45+GFP- (CAR-) cell populations for each CAR design. GFP expression denotes CAR-transduced cells.

Tissue	Condition	T cell (CD3+)			NK cell (CD56+)			B cell (CD19+)			Myeloid cell (CD33+)		
Spleen	4-1BB/ζ (GFP+)	5.83	5.77	2.46	0.16	1.6	0	20.2	24.4	10.7	62.6	53.5	77.9
	2B4/ζ (GFP+)	4.81	1.56	6.64	0.2	0.73	0	10.4	27.2	62.2	79.3	63.5	12.8
	CD3ζ (GFP+)	0.33	0.84	1.31	0.26	1.96	0.44	17.8	4.76	65.6	75.4	88.5	8.18
	4-1BB/ζ control (GFP-)	13.7	14.3	12.7	0.27	0.85	0.23	71	73.7	73	5.59	2.47	6.8
	2B4/ζ control (GFP-)	8.05	3.12	9.34	0.18	0.17	0.24	75.6	87.8	74	5.59	2.08	0.49
	CD3ζ control (GFP-)	1.39	10.4	1.51	0.26	0.85	0.34	84.8	58.4	73.6	3.9	17.2	0.56
Bone marrow	4-1BB/ζ (GFP+)	0.3	0.77	0.091	0.044	0.26	0.091	0.48	1.54	0.37	93.5	81	96.3
	2B4/ζ (GFP+)	0.34	0.39	0.27	0.11	0.39	0.14	0.76	2.71	1.08	91.1	87.2	92
	CD3ζ (GFP+)	0	0	0	0	0	0.33	1.13	1.71	0.31	94.6	85.5	93.1
	4-1BB/ζ control (GFP-)	0.66	0.97	1.14	0.043	0.17	0.071	58.1	79.9	57.2	30.6	11.5	33.9
	2B4/ζ control (GFP-)	0.93	0.92	0.5	0.04	0.038	0.089	56.4	66.6	60.6	31.4	26	29
	CD3ζ control (GFP-)	0.083	0	0.34	0	0	0.16	72	82.8	24.1	19.1	11.2	62.6

Table S3: Antibodies used for flow cytometry and western blotting

Target	Application	Isotype or source	Conjugate	Clone	Manufacturer	Catalogue number
CD34	Flow cytometry	Mouse IgG1 κ	APC	581	Biolegend	343510
CD201	Flow cytometry	Rat IgG1 κ	PE	RCR-401	Biolegend	351940
Lin antibody cocktail	Flow cytometry	Mouse IgG1 κ, Mouse IgG2b κ	BV421	UCHT1, HCD14, 3G8, HIB19, 2H7, HCD56	Biolegend	348805
CD45RA	Flow cytometry	Mouse IgG2b κ	BV510	HI100	Biolegend	304142
CD90	Flow cytometry	Mouse IgG1 κ	APC-cy7	5E10	Biolegend	328132
CD45	Flow cytometry	Mouse IgG1 κ	APC-cy7	HI30	Biolegend	304014
Mouse CD45	Flow cytometry	Rat IgG2b κ	e450	30-F11	eBioscience	48-0451-82
CD3	Flow cytometry	Mouse IgG2a κ	PE	OKT3	eBioscience	12-0037-42
CD56	Flow cytometry	Mouse IgG1 κ	BV510	HCD56	Biolegend	318340
CD19	Flow cytometry	Mouse IgG1 κ	APC	HIB19	eBioscience	17-0199-42
CD33	Flow cytometry	Mouse IgG1 κ	PE-cy7	WM-53	eBioscience	25-0338-42
CD69	Flow cytometry	Mouse IgG1 κ	BV650	FN50	Biolegend	310933
phospho-NF-κB	Western blotting	Rabbit	-	-	Cell Signalling Technologies	3033
phospho-Akt	Western blotting	Rabbit	-	-	Cell Signalling Technologies	9271
Anti-rabbit IgG, horseradish peroxidase linked	Western blotting	Goat	Horseradish peroxidase linked	-	Cell Signalling Technologies	7074

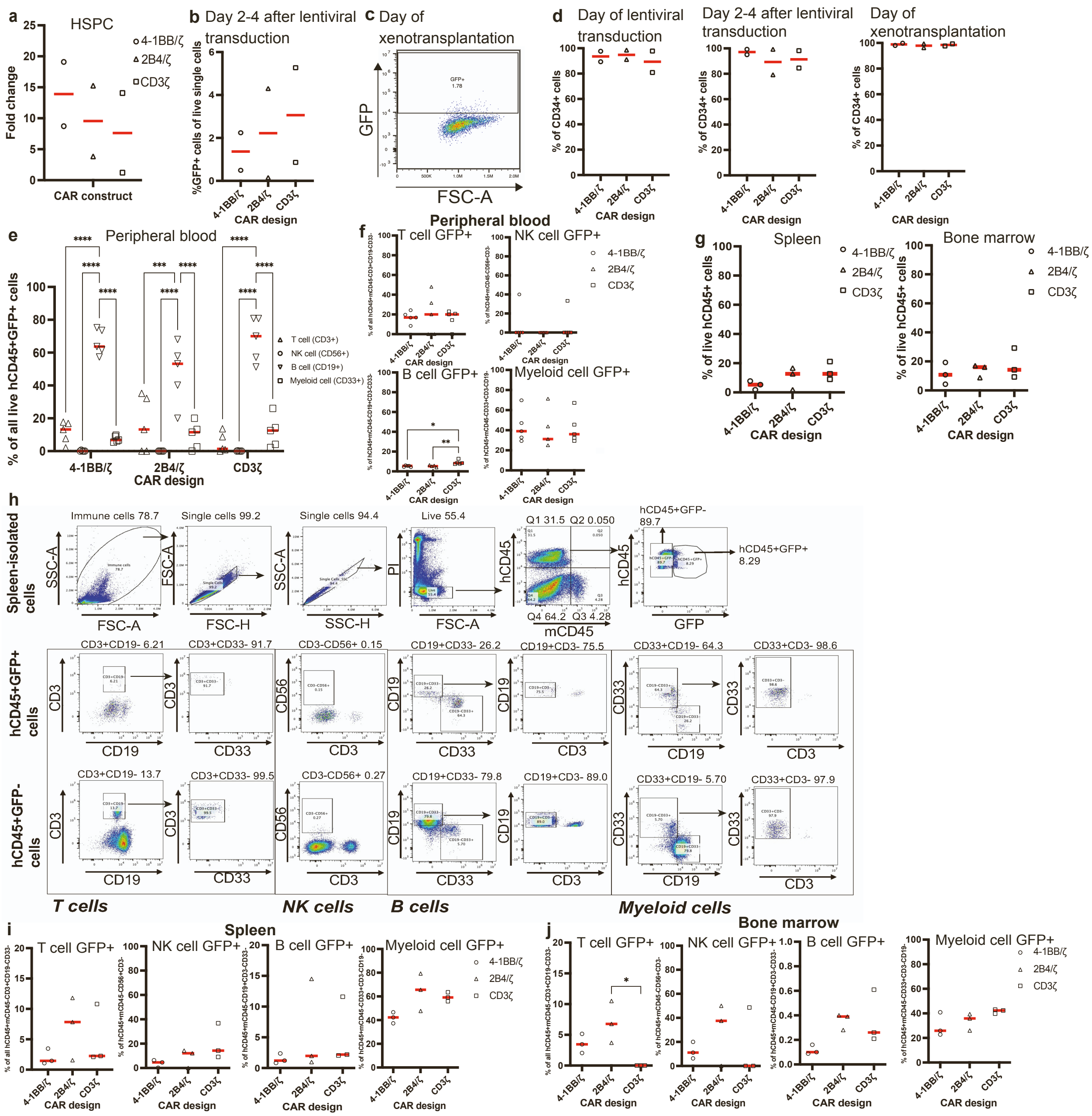

Figure S1: Development of HSPC-derived CAR-immune cells **a** Fold change in live cell number at day 14 compared with day 0 of HSPC expansion culture across the CAR designs. **b** Bar graph showing the percentage of GFP+ cells of live, single CD34+ cells across CAR designs at days 2-4 after lentiviral transduction. **c** Representative flow cytometry plot on the day of xenotransplantation showing GFP+ cells gated on live, single cells. **d** Percentage of CD34+ cells gated on live, single cells across CAR designs on the day of lentiviral transduction (left), 2-4 days after transduction (middle) and on the day of xenotransplantation (right). **e** Immune cell composition in peripheral blood at 15 weeks post-transplantation across CAR designs showing the proportion of T (CD3+), NK (CD56+), B (CD19+) and myeloid cells (CD33+) gated on live, single hCD45+GFP+ cells. **f** Percentage of T (CD3+)(top left), NK (CD56+)(top right), B (CD19+)(*p=0.0209)(bottom left) and myeloid cells (CD33+)(bottom right) in the peripheral blood that are hCD45+GFP+ across CAR designs. **g** Percentage of GFP+ cells out of the live, hCD45+ single cells in the spleen (left) and bone marrow (right) across CAR designs. **h** Representative gating strategy for spleen-isolated cells including gating of immune, single and live cells followed by sequential separation of hCD45+mCD45-GFP+ and hCD45+mCD45-GFP- populations (first row), isolation of T, NK, B and myeloid cells within the hCD45+mCD45-GFP+ population (second row) and the corresponding gating strategy within the hCD45+mCD45-GFP- population (third row). **i** Percentage of T (CD3+)(far left), NK (CD56+)(centre left), B (CD19+)(centre right) and myeloid cells (CD33+)(far right) that are hCD45+GFP+ across CAR designs in the spleen. **j** Percentage of T (CD3+)(*p=0.0175)(far left), NK (CD56+)(centre left), B (CD19+)(centre right) and myeloid cells (CD33+)(far right) that are hCD45+GFP+ across CAR designs in the bone marrow. **a, b, d, e, f, g, i, j** represent individual values with red crossbars indicating the median. No symmetric measure of variance is displayed. **a, b, d** n=2; **e, f** n=5**; g, i, j** n=3. **e,** two-way ANOVA with Tukey's post-hoc test (all pairwise comparisons; selected comparisons shown between cell lineages within each CAR design) **f, g, i, j** one-way ANOVA with Tukey's post-hoc test (all pairwise comparisons across CAR designs), *p < 0.05, **p ≤ 0.01, ***p ≤ 0.001, ****p ≤ 0.0001

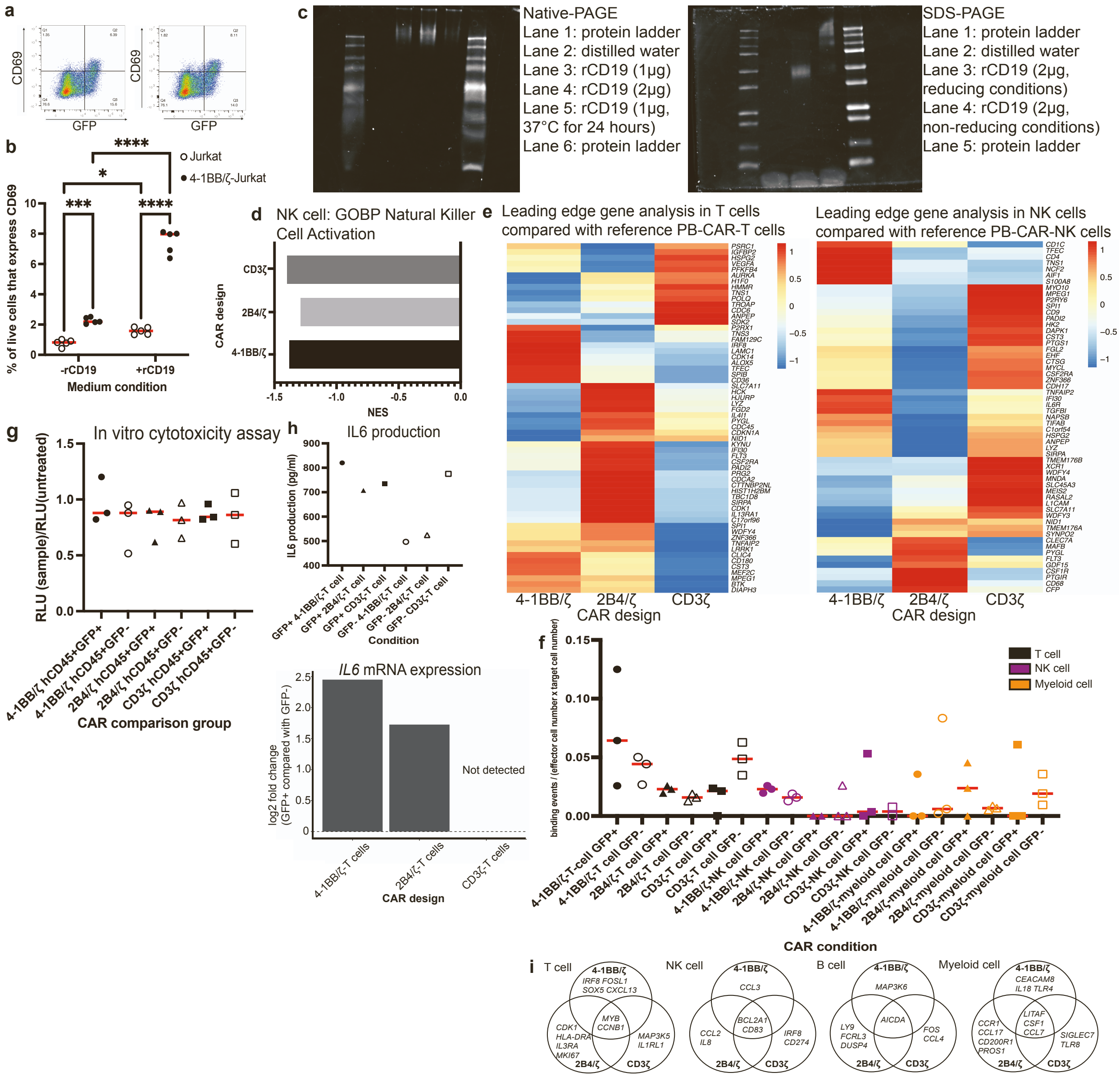

Figure S2: Validation of antigen-specific activation and functional characterisation of HSPC-derived CAR-immune cells. **a** Representative flow cytometry plots of Jurkat (GFP-) and 4-1BB/ζ-Jurkat cells (GFP+) cultured in control medium (left) or rCD19-supplemented medium (right) gated on live, single Jurkat cells. **b** Percentage of CD69+ cells gated on live single cells, for Jurkat (GFP-) and 4-1BB/ζ-Jurkat cells (GFP+) cultured in control or rCD19-supplemented medium (*p=0.0468). **c** Native-PAGE and SDS-PAGE gels stained with Coomassie Blue (Bio-Safe) with lane annotations indicating amounts of rCD19 protein and condition per lane. **d** Bar graph showing NES from GSEA for the GOBP Natural Killer Cell Activation pathway in NK cells across CAR designs following antigen-specific activation. **e** Leading-edge gene contribution heatmaps from GSEA for T cells using reference activated PB-CAR-T cells (left) and for NK cells using reference activated PB-CAR-NK (right) cells across the different CAR designs. The top 30 contributing genes were selected, combined across designs and row-scaled to visualise relative contributions across CAR designs. **f** Quantification of effector cell-target cell binding events from quantitative phase imaging data. Binding events are normalised to effector and target cell number [binding event / (effector cell number x target cell number)]. Wells lacking detectable effector cells or target cells were excluded from analysis. Data are shown descriptively due to the limited number of evaluable wells in certain conditions. **g** In vitro cytotoxicity assay showing relative luminescence units (RLU) normalised to target cell-only wells for each condition across CAR designs. GFP+ indicates CAR+ cells; GFP- indicates non-transduced control cells. **h** IL6 production from CAR-T cells (GFP+) compared with non-transduced control T cells (GFP-) across CAR designs measured by ELISA. Cytokine concentrations are presented as pg/ml (top). Bar graphs showing the log2FC of *IL6* mRNA expression in T cells comparing GFP+ and GFP- cells across CAR designs after 24 hours of antigen-specific activation. *IL6* expression was not detected in CD3ζ-T cells (bottom). **i** Venn diagrams illustrating candidate upregulated genes following antigen-specific activation in T cells (far left), NK cells (centre left), B cells (centre right) and myeloid cells (far right) with each CAR design represented as a separate set. Genes satisfying expression and effect size thresholds (CPM > 1 and |log2FC| > 1 between CAR designs) were included. **b, f, g** represent individual values with median values indicated by red crossbars **b** n=5, two-way ANOVA with Tukey's post-hoc test (all pairwise comparisons; selected comparisons between culture medium and cell type shown). **f** Individual points represent independent wells, n=2/3 due to well exclusion **g** n=3, two-way ANOVA with Šídák's multiple comparisons test (comparisons restricted to GFP+ (CAR+) and GFP- (CAR-) populations within each CAR design) **h** ELISA n=1. **d, e, h, i** RNA-sequencing n=1. *p < 0.05, **p ≤ 0.01, ***p ≤ 0.001, ****p ≤ 0.0001

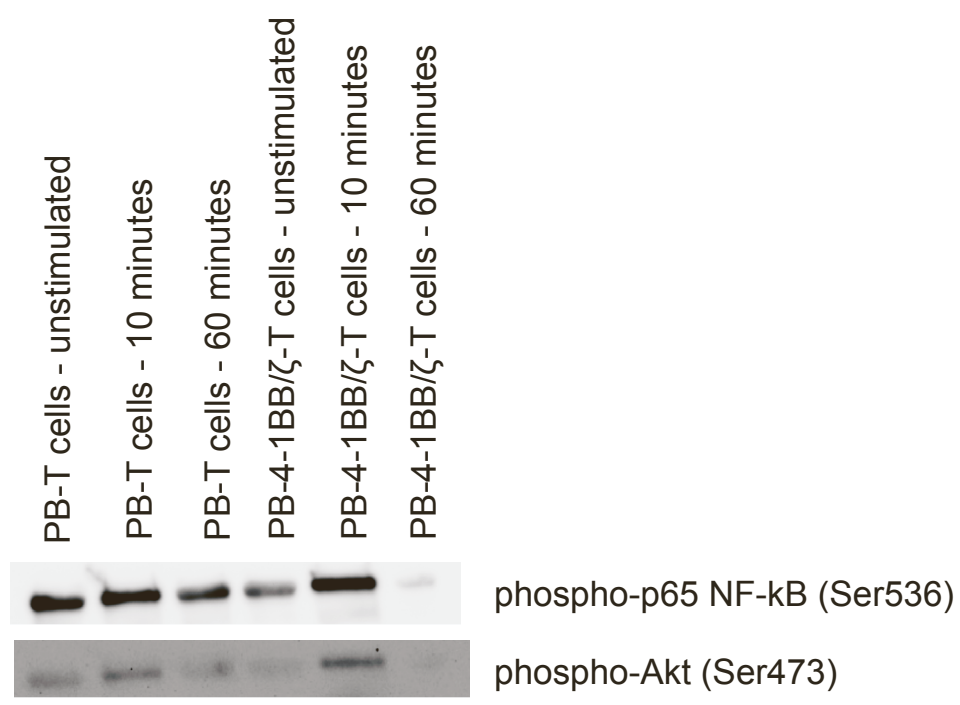

Figure S3: Validation of 10-minute antigen-stimulation. Western blot of peripheral blood (PB)-T cells and PB-4-1BB/ζ-T cells, assessing phosphorylation of p65 NF-kB (Ser536) and Akt (Ser473) in PB-T cells and PB-4-1BB/ζ-T cells that are unstimulated, have undergone 10 minutes of antigen-specific activation or 60 minutes of antigen-specific activation, n=1 samples.

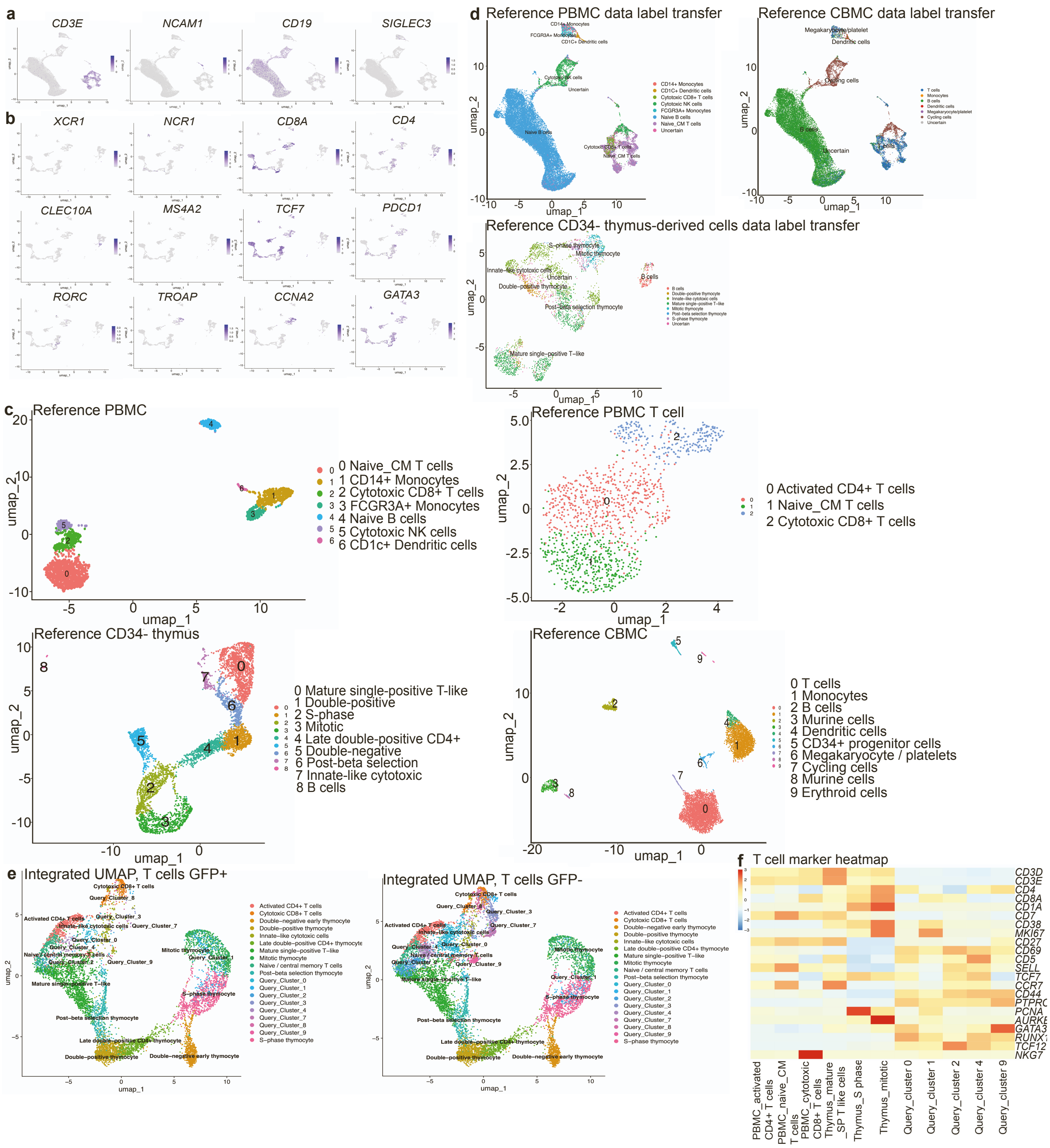
a
CD3E
NCAM1
CD19
SIGLEC3
b
XCR1
NCR1
CD8A
CD4
CLEC10A
MS4A2
TCF7
PDCD1
RORC
TROAP
CCNA2
GATA3
d
Reference PBMC data label transfer
Reference CBMC data label transfer
Reference CD34- thymus-derived cells data label transfer
c
Reference PBMC
0 Naive_CM T cells
1 CD14+ Monocytes
2 Cytotoxic CD8+ T cells
3 FCGR3A+ Monocytes
4 Naive B cells
5 Cytotoxic NK cells
6 CD1c+ Dendritic cells
Reference PBMC T cell
0 Activated CD4+ T cells
1 Naive_CM T cells
2 Cytotoxic CD8+ T cells
Reference CD34- thymus
0 Mature single-positive T-like
1 Double-positive
2 S-phase
3 Mitotic
4 Late double-positive CD4+
5 Double-negative
6 Post-beta selection
7 Innate-like cytotoxic
8 B cells
Reference CBMC
0 T cells
1 Monocytes
2 B cells
3 Murine cells
4 Dendritic cells
5 CD34+ progenitor cells
6 Megakaryocyte / platelets
7 Cycling cells
8 Murine cells
9 Erythroid cells
umap_1
umap_2
e
Integrated UMAP, T cells GFP+
Integrated UMAP, T cells GFP-
f
T cell marker heatmap
CD3D
CD3E
CD4
CD8A
CD1A
CD7
CD38
MKI67
CD27
CD69
CD5
SELL
TCF7
CCR7
CD44
PTPRC
PCNA
AURKB
GATA3
RUNX3
TCF12
NKG7
PBMC_activated CD4+ T cells
PBMC_naive_CM T cells
PBMC_cytotoxic CD8+ T cells
Thymus_mature _SP T like cells
Thymus_S phase
Thymus_mitotic
Query_cluster 0
Query_cluster 1
Query_cluster 2
Query_cluster 4
Query_cluster 9

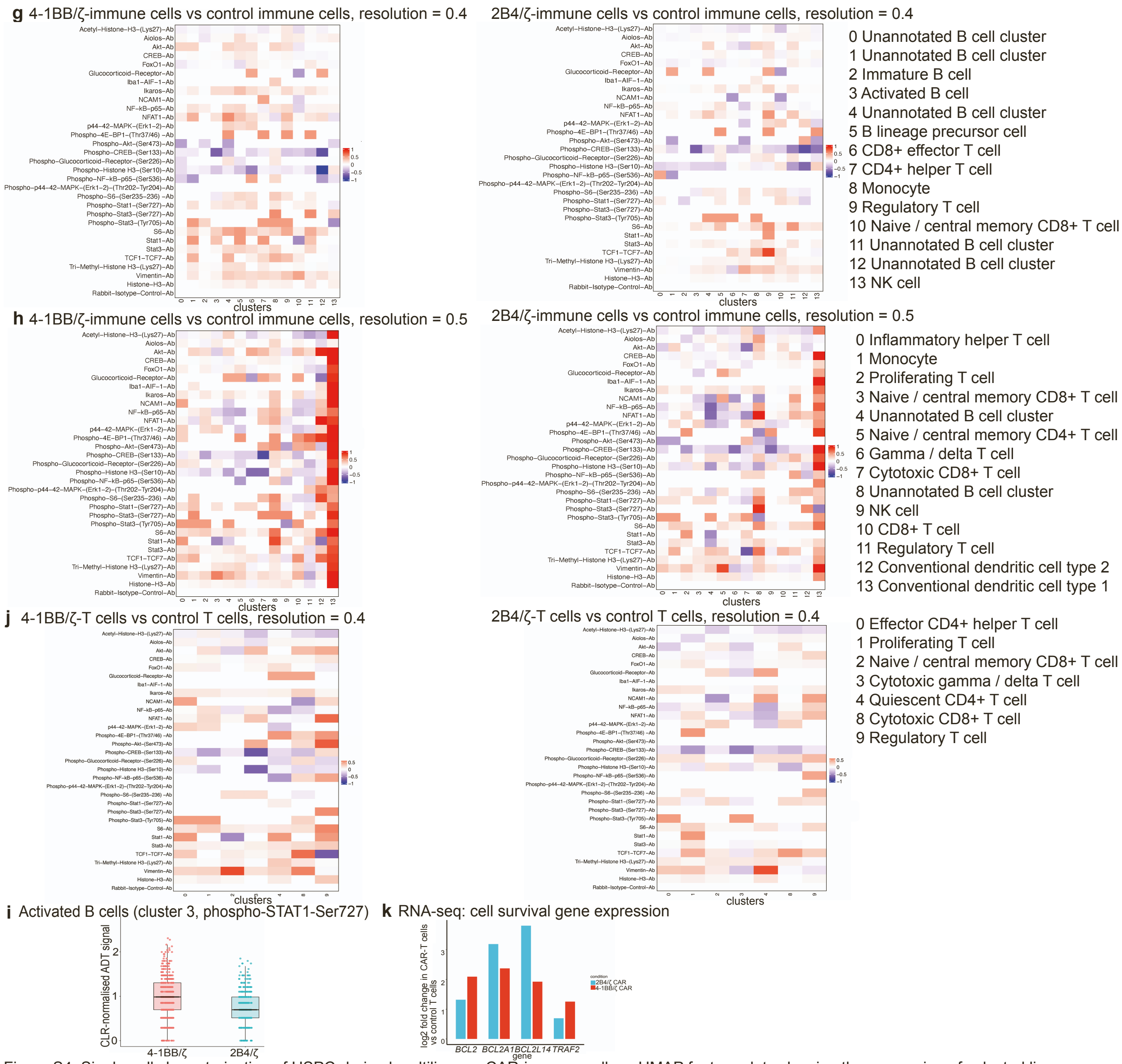

Figure S4: Single-cell characterisation of HSPC-derived multilineage CAR-immune cells. **a** UMAP feature plots showing the expression of selected lineage marker genes across pooled spleen-isolated HSPC-derived immune cells (resolution, 0.4). **b** UMAP feature plots of additional marker gene expression across re-clustered subsets (clusters 6, 7, 8, 9, 10, 13 from **a**; resolution, 0.5). **c** UMAP feature plots of reference PBMC (top left), subset T cells from reference PBMC (top right), reference thymus CD34- cells (bottom left) and reference CBMC (bottom right) processed independently using identical QC filtering and log-normalisation as HSPC-derived CAR-immune cells from **a**. **d** UMAP of pooled HSPC-derived immune cells (from **a**) coloured by predicted cell identity from label transfer using reference PBMC (top left), reference CBMC (top right) and HSPC-derived T cells (clusters 6, 7, 9, 10 from **a**; resolution, 0.4) coloured by predicted cell identity from label transfer using reference thymus CD34- cell (bottom left) datasets. Predicted identities were filtered by prediction score (threshold ≥ 0.5), with low-confidence assignments labelled as "Uncertain". **e** Three-way integrated UMAP of reference PBMC, thymus CD34- and HSPC-derived T cells (GFP+)(left) and HSPC-derived T cells (GFP-)(right) generated using shared gene features and CCA-based integration. Original cluster labels were maintained. **f** Heatmap of selected marker gene expression (rows) across selected reference PBMC, thymus CD34- and HSPC-derived T cell clusters (columns). Values represent row-scaled average single-cell RNA expression. Columns are not hierarchically clustered. **g** Heatmaps of CLR-normalised ADT signalling features with median differences between 4-1BB/ζ-immune cells (GFP+) vs non-transduced control immune cells (GFP-)(left) and 2B4/ζ-immune cells (GFP+) vs non-transduced control immune cells (GFP-)(right) across all HSPC-derived immune cells from **a**, resolved at 0.4. **h** Heatmaps of CLR-normalised ADT signalling features with median differences between 4-1BB/ζ-immune cells (GFP+) vs non-transduced control immune cells (GFP-)(left) and 2B4/ζ-immune cells (GFP+) vs non-transduced control immune cells (GFP-)(right)(clusters 6, 7, 8, 9, 10, 13 from **a**; resolution, 0.5). **i** Boxplots overlaid with individual cells, showing CLR-normalised ADT expression of pSTAT1-(Ser727) for cluster 3 (from **a**, resolution, 0.4). **j** Heatmaps of CLR-normalised ADT signalling features with median differences between 4-1BB/ζ-T cells (GFP+) vs non-transduced control T cells (GFP-)(left) and 2B4/ζ-T cells (GFP+) vs non-transduced control T cells (GFP-)(right)(clusters 6, 7, 9, 10 from **a**; resolution, 0.4). **k** Bar graph showing log2FC expression of selected survival-associated genes from RNA-seq analysis in 4-1BB/ζ-, 2B4/ζ-T cells (GFP+) compared with respective non-transduced control T cells (GFP-) after 24-hour activation. **g**, **h**, **j** Only clusters present in both conditions are shown. Rows represent signalling proteins or post-translational modifications and columns represent clusters. GFP+ denotes CAR+, CAR-expressing cells; GFP- denotes non-transduced control cells. RNA-seq and InTraSeq samples: n=1 biological replicate. Abbreviations: CM, central memory

Movie S1: Quantitative phase imaging of HSPC-derived 4-1BB/ζ-T cells (GFP+) co-cultured with A549-CD19-mKate target cells. Phase contrast videos of a single field of view of HSPC-derived 4-1BB/ζ-T cells (GFP+) co-cultured with A549-CD19-mKate target cells at a 1:1 E:T ratio and imaged at 10x magnification at 15-minute intervals for 48 hours. Scale bar, 200μm, related to Figure 2.

Movie S2: Quantitative phase imaging of HSPC-derived 4-1BB/ζ-NK cells (GFP+) co-cultured with A549-CD19-mKate target cells. Phase contrast videos of a single field of view of HSPC-derived 4-1BB/ζ-NK cells (GFP+) co-cultured with A549-CD19-mKate target cells at a 1:1 E:T ratio and imaged at 10x magnification at 15-minute intervals for 48 hours. Scale bar, 200μm, related to Figure 2.

Movie S3: Quantitative phase imaging of HSPC-derived 4-1BB/ζ-myeloid cells (GFP+) co-cultured with A549-CD19-mKate target cells. Phase contrast videos of a single field of view of HSPC-derived 4-1BB/ζ-myeloid cells (GFP+) co-cultured with A549-CD19-mKate target cells at a 1:1 E:T ratio and imaged at 10x magnification at 15-minute intervals for 48 hours. Scale bar, 200μm, related to Figure 2.
