# Supplementary material for: CAR design equips diverse immune cells with unique antigen-specific activation identities
Source: Mol Ther Oncol. 2026 Jul 29;34(3):201312. doi: 10.1016/j.omton.2026.201312 (PMC13521014; doi:10.1016/j.omton.2026.201312)
Supplement: Supplementary file 2 [file mmc5.pdf]

Molecular Therapy
Oncology
Original Article

CAR design equips diverse immune cells with unique antigen-specific activation identities

Ayano Sugiyama-Finnis,[1] Takaharu Kimura,[1] Kiyoko Izawa,[1] Yuko Kawano,[1] Takao Yogo,[1] Hyojung Jeon,[1] Tsuyoshi Fukushima,[1] Ryoji Ito,[2] and Satoshi Yamazaki[1,3]

[1]Division of Cell Regulation, Center for Experimental Medicine and Systems Biology, The Institute of Medical Science, The University of Tokyo, Tokyo 108-8639, Japan; [2]Central Institute for Experimental Medicine and Life Science, Kawasaki, Kanagawa 210-0821, Japan; [3]Division of Cell Engineering, Center for Stem Cell Biology and Regenerative Medicine, The Institute of Medical Science, The University of Tokyo, Tokyo 108-8639, Japan

Hematopoietic stem and progenitor cells (HSPCs) are multipotent cells capable of generating all hematopoietic lineages, representing a powerful platform for regenerative immunotherapy. In parallel, chimeric antigen receptor (CAR) engineering has extended beyond T cells, which have achieved success in treating B cell malignancies, to broader immune cell types alongside cell type-specific CAR design. However, how CAR architecture modulates activation phenotypes across immune lineages remains poorly understood. Here, we systematically compared previously validated, immune cell-optimized CARs across immune cell types using a human HSPC-derived multilineage CAR-immune cell platform. HSPC-derived T, natural killer (NK), and myeloid cells demonstrated immune activation across different CAR designs, whereas B cells showed CAR design dependency for activation. Hallmark pathway analysis revealed that immune lineage was the dominant determinant of activation-induced transcriptional states, while CAR design modulated signaling programs within lineage-constrained circuits, notably proliferation, inflammation, and cytokine responses. Single-cell InTraSeq highlighted differential activation of signaling pathways depending on CAR design and lineage identity with links to downstream transcriptional programs across T, B, and myeloid cells. Although exploratory and performed with limited sample sizes, this demonstrates the activated phenotypes and pathways mediated by CAR design and lineage identity that may inform the development of next-generation CAR-immune cells with tailored activation-induced characteristics.

INTRODUCTION

Hematopoietic stem and progenitor cells (HSPCs) are multipotent cells capable of self-renewal and multilineage differentiation, generating the hematopoietic lineages.[1] Advances in *ex vivo* HSPC culture enable genome modification while preserving stem-defining properties,[2,3] providing a platform to investigate genome engineering strategies for cellular immunotherapies.[4]

Chimeric antigen receptor (CAR)-T cell therapies are immunotherapies approved for relapsed or refractory B cell malignancies.[5] CARs are recombinant receptors comprising an antigen-binding single-chain variable fragment (scFv), a transmembrane domain, and signaling domains derived from T cell receptor signaling such as CD3ζ, CD28, or 4-1BB,[5,6] enabling major histocompatibility complex-independent antigen recognition and activation of effector programs.

Building on the success of CAR-T cells, CAR-engineering has expanded to additional immune cell types, including natural killer (NK) cells,[7,8] macrophages,[9] and neutrophils.[10,11] More recently, efforts to generate CAR-engineered HSPCs and hematopoietic stem cells (HSCs) have introduced the concept of multilineage CAR-immune cell systems, enabling the generation of diverse CAR-expressing immune cell types from a common precursor.[12,13] Alternative effector cell types may offer advantages over T cells such as a reduced risk of toxicities and a greater diversity of effector mechanisms.[14] Consistent with this, it has been demonstrated that a combined CAR-immune cell therapy can modestly improve median survival in a murine glioblastoma model, with distinct immune cell combinations exhibiting differential anti-tumor activity and tumor microenvironment-modulating behaviors.[15]

Despite these advances, CAR designs have largely been optimized within a T cell-centric signaling framework. While conventional CAR-T receptors can induce proximal signaling events across multiple immune cell types including phosphorylation of key signaling intermediates such as SYK and ERK1/2,[10,16] attempts to optimize CAR activity have prompted the exploration of cell type-specific domains.[16] The introduction of myeloid-associated signaling domains was reported to increase NF-κB pathway activation resulting in stronger inflammatory and tumor-suppressing phenotypes of CAR macrophages.[17,18] This has included modifications of CD3ζ-based CAR constructs through addition of the macrophage signaling domain, the Toll-like receptor 4 intracellular Toll/interleukin-1-receptor (TIR) domain, or replacement of CD3ζ with a MyD88

Received 11 January 2026; accepted 27 July 2026;
https://doi.org/10.1016/j.omton.2026.201312.

Correspondence: Satoshi Yamazaki, Division of Cell Regulation, Center for Experimental Medicine and Systems Biology, The Institute of Medical Science, The University of Tokyo, Tokyo 108-8639, Japan.
E-mail: y-sato4@ims.u-tokyo.ac.jp

© 2026 The Author(s).
Published by Elsevier Inc. on behalf of The American Society of Gene and Cell Therapy.
This is an open access article under the CC BY license (http://creativecommons.org/licenses/by/4.0/).

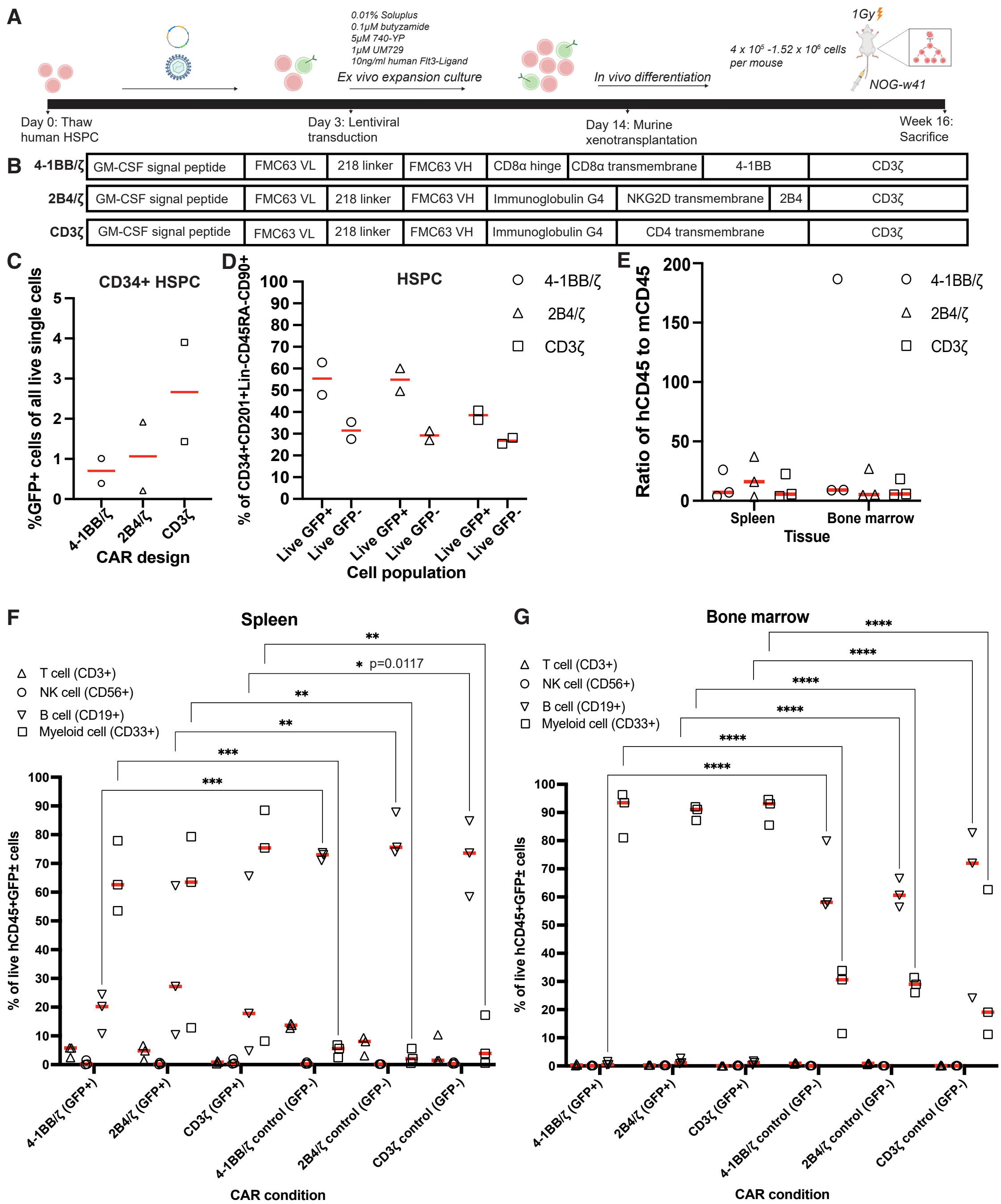

Figure 1. Establishing a multilineage CAR-immune cell system from engineered human HSPCs using a NOG-w41 murine xenotransplantation model

(A) Schematic of the experimental workflow. Human cord blood-derived CD34+ HSPCs underwent lentiviral transduction with previously validated, anti-CD19 CAR constructs and were cultured in a chemically defined *ex vivo* expansion system. Cells were subsequently xenotransplanted into sub-lethally irradiated NOG-w41 mice for *in vivo* differentiation, and immune cells were harvested from the spleen and bone marrow at 16 weeks post-transplantation. (B) Linear protein schematics of the previously validated anti-CD19 CAR designs used in this study (not to scale): 4-1BB/ζ, 2B4/ζ, and CD3ζ. (C) Percentage of GFP+ cells among live, single CD34+ HSPCs at the time of xenotransplantation for each CAR design. (D) Percentage of phenotypic HSPCs (CD34+CD201+Lin-CD45RA−CD90+) within live, single CAR-HSPC (GFP+; CAR+) and non-transduced control (GFP−; CAR−) HSPC fractions at the time of xenotransplantation across CAR designs. (E) Ratio of human CD45+ (hCD45+) to murine CD45+ (mCD45+) cells among live, single cells isolated from the spleen and bone marrow at 16 weeks post-xenotransplantation, reflecting human cell engraftment. (F) Distribution of immune cell lineages isolated from the spleen, shown as percentages of T cells (CD3+), NK cells (CD56+), B cells (CD19+), and myeloid cells (CD33+) among live, single hCD45+GFP+ (GFP+; CAR+) or non-transduced control hCD45+GFP− (GFP−; CAR−) cell populations for each CAR design. (G) Distribution of immune cell lineages isolated from

(legend continued on next page)

domain. NK cell-specific CAR design has explored NK cell-associated transmembrane and signaling domains such as NKG2D and DAP10, DAP12, or 2B4 domains, respectively, either replacing or complementing the CD8 and CD3ζ domains.[16,19] Although the optimal transmembrane domain remains unresolved, a dual 2B4 and CD3ζ intracellular domain[19] was shown to improve NK cell signaling.[16] In NK cells, cell type-matched CAR designs have demonstrated improved *in vivo* tumor clearance in murine xenograft models,[16] whereas CAR-T-like CD3ζ receptors expressed in neutrophils have been shown to elicit robust anti-tumor responses.[10] Collectively, these findings suggest that cell type-specific CARs are not universally optimal across all immune cell types.

These observations raise the question of whether validated CAR designs can shape activation phenotypes in immune cells beyond their matched lineage, with the potential to broaden the scope of antigen-specific immune responses. Here, we assess whether CAR design modulates activation-induced phenotypes across myeloid and lymphoid immune cells, focusing on transcriptional and signaling programs rather than a comprehensive functional evaluation.

Genomic modification of HSPCs prior to *in vivo* differentiation enables the simultaneous generation of multilineage CAR-engineered immune cell types from the same developmental environment. Using a chemically defined human HSC culture platform[2] alongside a murine xenotransplantation model,[20] we investigate CAR designs previously validated in screening studies for T cells, NK cells, and neutrophils. This enables the direct comparison of activation-induced molecular programs of functionally validated CAR designs in a manner that is aligned with current preclinical investigations. By integrating *ex vivo* HSPC modification with multilineage differentiation, this study enables systematic profiling of CAR design-dependent activation-induced molecular phenotypes across immune cell types. We explored antigen-specific transcriptional and intracellular signaling programs shaped by these CAR designs, including at single-cell resolution. While preliminary functional assays are investigated, the primary aim of this study is to define lineage-specific molecular programs, providing insight into how CAR design and effector immune cell identity shape immune activation and guide the development of next-generation CAR strategies.

RESULTS

A chemically defined lentiviral transduction and *ex vivo* human HSPC culture system enables the generation of multilineage CAR-immune cells

First, to establish multilineage CAR-expressing immune cells, CAR-engineering of human HSPCs and *in vivo* differentiation using NOG-w41 xenotransplantation was conducted, generating diverse immune cell types arising from the same *in vivo* environment (Figure 1A). We transduced human cord blood HSPCs (CD34+ cells) with lentiviral vectors encoding one of three CAR constructs, previously validated in T cells, NK cells, and neutrophils, namely, the CD8α-CD8α-4-1BB-CD3ζ (4-1BB/ζ),[21] IgG4-NKG2D-2B4-CD3ζ (2B4/ζ),[10] and IgG4-CD4-CD3ζ (CD3ζ),[10] respectively, and expanded the transduced HSPCs in a chemically defined medium (Figure 1B; Figure S1A).[2] 2B4/ζ and CD3ζ constructs were adapted by incorporating an anti-CD19-binding domain (Figure 1B). GFP served as a lentiviral reporter. Following lentiviral transduction, GFP+ populations were detectable across the CAR designs (Figure S1B). Furthermore, at day 14 of chemically defined *ex vivo* expansion culture, flow cytometry revealed a comparable proportion of GFP+ cells in the live CD34+ population across all three CAR-HSPC types (Figure 1C; Figure S1C). Flow cytometric assessment on the day of lentiviral transduction, at early post-transduction time points (day 2–4) and on the day of transplantation confirmed retention of a substantial CD34+ population prior to transplantation (Figure S1D). The phenotypic HSPC population (CD34+CD201+Lin-CD45RA−CD90+) was preserved in both CAR-HSPCs (GFP+; CAR+) and non-transduced control HSPCs (GFP−; CAR−) (Figure 1D). Preservation of phenotypic HSPCs during *ex vivo* expansion was important for enriching the population of cells that possess engraftment and multilineage differentiation capacity. Xenotransplants were conducted in two independent experiments using commercially sourced pooled human cord blood-derived CD34+ HSPCs from different manufacturing lots with three recipient mice per condition in the first cohort and five recipient mice per condition in the second cohort. These results demonstrate that this system maintains phenotypic CAR-HSPCs for subsequent NOG-w41 xenotransplantation.

Peripheral blood draws of NOG-w41 mice at 15 weeks post-transplantation revealed the presence of multilineage human immune cells across T cells, NK cells, B cells, and myeloid cells in the human CD45+ (hCD45+) and murine CD45− (mCD45−) GFP+ fraction across the CAR designs (Figure S1E; Table S1). Analysis of GFP expression within individual immune lineages demonstrated the presence of CAR-T, CAR-B, and CAR-myeloid cells across all the CAR designs (Figure S1F). GFP+ NK cells exhibited greater variability, possibly due to the overall low abundance of NK cells in the peripheral blood (Figure S1E; Table S1). GFP+ B cells were detected at significantly different frequencies across CAR designs and were highest in the CD3ζ condition (Figure S1F). At 16 weeks post-transplantation, NOG-w41 mice were sacrificed and human chimerism was detected in both the spleen and bone marrow across

the bone marrow, shown as percentages of T cells (CD3+), NK cells (CD56+), B cells (CD19+), and myeloid cells (CD33+) among live, single hCD45+GFP+ cells (GFP+; CAR+) or non-transduced control hCD45+GFP− (GFP−; CAR−) cell populations for each CAR design. Individual biological replicates are shown with median values indicated by red horizontal crossbars. In accordance with the small sample sizes, no symmetric measure of variance is displayed. GFP expression denotes CAR-transduced cells. Data are presented as $n = 2$ (C and D) and $n = 3$ (E–G). Statistical analysis: two-way ANOVA with Tukey's post hoc multiple comparisons test, with all pairwise comparisons performed (E) and two-way ANOVA with Šidák's multiple comparisons test with comparisons restricted to GFP+ (CAR+) and GFP− (CAR−) populations within each CAR design and immune cell lineage (F and G). $^{*}p < 0.05$, $^{**}p \leq 0.01$, $^{***}p \leq 0.001$, and $^{****}p \leq 0.0001$. Schematic created with BioRender.com.

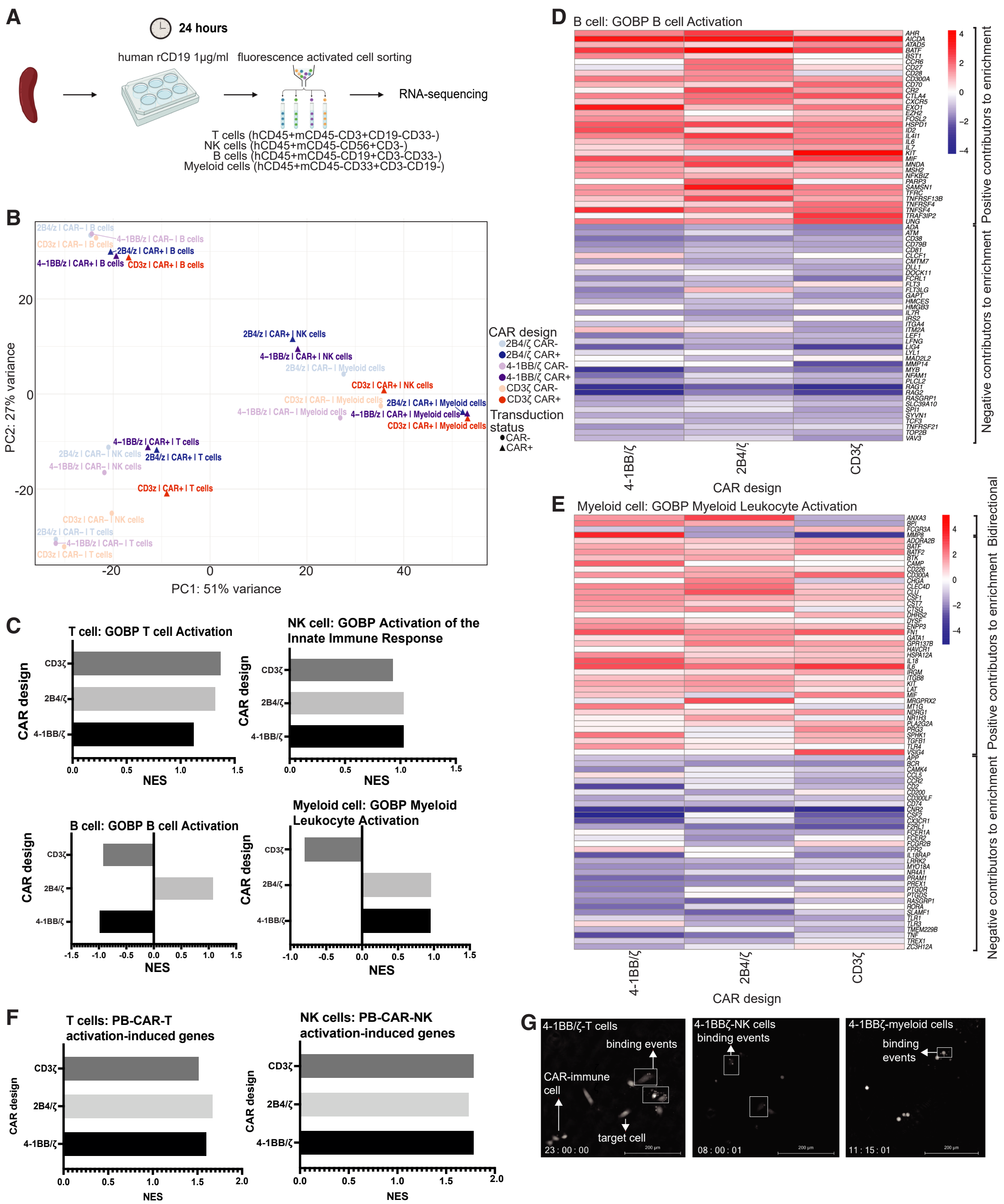

Figure 2. Antigen exposure initiates immune activation gene expression programs in HSPC-derived myeloid and lymphoid CAR-immune cells

(A) Schematic representing the experimental workflow for *ex vivo* antigen-specific activation of spleen-derived, human 4-1BB/ζ-, 2B4/ζ-, and CD3ζ-immune cells with recombinant human CD19 (rCD19) antigen for 24 h. Cells were subsequently sorted into T cell, NK cell, B cell, and myeloid cell fractions for GFP+ (CAR+) and GFP− (non-transduced control; CAR−) populations prior to RNA-seq. (B) Principal-component analysis (PCA) of RNA-seq data from the indicated immune cell types across CAR designs following antigen-specific activation. Samples are annotated by transduction status (GFP+, CAR+; GFP−, CAR−), immune cell type, and CAR design. (C) Bar graphs showing the normalized enrichment score (NES) from GSEA for the GOBP gene sets; T cell Activation in T cells (top left), Activation of Innate Immune Response in NK cells (top right), B cell Activation in B cells (bottom left), and Myeloid Leukocyte Activation in myeloid cells (bottom right) across CAR designs following antigen-specific activation. (D) Heatmap showing gene-level contributions to the GOBP B cell Activation gene set enrichment following antigen-specific activation across CAR designs for 4-1BB/ζ-, 2B4/ζ-, and CD3ζ-B cells. (E) Heatmap showing gene-level contributions to the GOBP Myeloid Leukocyte Activation gene set enrichment following antigen-specific activation of 4-1BB/ζ-, 2B4/ζ-, and CD3ζ-myeloid cells. (F) Bar graphs showing NES from GSEA for differentially expressed genes in activated reference PB-CAR-T cells (left) and

(legend continued on next page)

all three CAR-HSPC-transplanted conditions (Figure 1E), indicating that transplanted human HSPCs retained *in vivo* engraftment capability. A proportion of hCD45+ cells in the spleen and bone marrow were also GFP+ across the CAR designs (Figure S1G). Within the hCD45+mCD45− population, multilineage immune cells were detected including T (CD3+CD19−CD33−), NK (CD56+CD3−), B (CD19+CD3−CD33−), and myeloid (CD33+CD3−CD19−) cells in the GFP+ and GFP− populations (Figures 1F and 1G; Figure S1H; Table S2). Analysis of GFP+ expression within spleen-derived T cells, NK cells, B cells, and myeloid cells demonstrated comparable frequencies of GFP+ cells across the CAR designs (Figure S1I). Similarly, GFP+ frequencies within the immune cell types were largely comparable between CAR designs in the bone marrow (Figure S1J). However, no T cells could be detected in the bone marrow of CD3ζ-HSPC-transplanted mice, resulting in significantly reduced GFP+ T cell frequencies compared with 2B4/ζ-HSPC-transplanted mice (Figure S1J). The CAR design-dependent difference in GFP+ B cell frequency observed in the peripheral blood (Figure S1F) was not detected in the spleen or bone marrow. Although reduced statistical power in these tissues cannot be excluded due to a smaller sample size, all downstream analyses were performed using spleen-isolated immune cells, owing to its higher cellular yield and more diverse lineage representation compared with the peripheral blood and bone marrow. Therefore, this peripheral blood-specific difference in GFP+ B cell frequencies is unlikely to be a major contributor to the CAR design-dependent phenotypes presented here. Hereafter, cell-specific CAR designs will be denoted as "*CAR design-cell type*" (e.g., 4-1BB/ζ-T cells), while "*CAR-cell type*" will refer to CAR-expressing immune cells irrespective of design (e.g., CAR-T cells). Lineage distribution indicated skewing, with NK cells detected at the lowest frequency and myeloid cells as the most abundant population in the hCD45+GFP+ compartment. In contrast, the hCD45+GFP− compartment was B cell dominant (Figures 1F and 1G). Together, this *ex vivo* CAR-HSPC culture and xenotransplantation platform successfully generates diverse CAR-immune cells from a common CAR-engineered HSPC precursor cell type.

Antigen exposure induces transcriptional activation programs in CAR-immune cells across myeloid and lymphoid lineages

To determine whether multilineage CAR-immune cells generated *in vivo* could mount antigen-specific activation responses, *ex vivo* antigen stimulation was conducted to measure CAR-dependent transcriptional activation (Figure 2A). Splenocytes from CAR-HSPC transplanted NOG-w41 mice were used, as the spleen provided the highest yield and most diverse representation of human immune cell lineages for downstream transcriptional analysis. These cells were cultured with recombinant human CD19 (rCD19) antigen for 24 h, and this method was validated in Jurkat cells expressing the 4-1BBζ receptor (4-1BBζ-Jurkat). These cells served as a proxy for antigen-dependent activation of splenic CAR-immune cells, with CD69 used as an early activation marker (Figures S2A and S2B).[22] Native-PAGE and non-reducing and reducing SDS-PAGE confirmed that rCD19 formed higher-order complexes (Figure S2C) consistent with previous reports demonstrating the requirement of ligand-induced CAR clustering[23] for CAR-dependent activation and the formation of oligomeric CD19 complexes in solution, sufficient to induce CAR-dependent activation.[24] These data support the use of rCD19 stimulation as a perturbation to probe CAR-associated activation responses. hCD45+mCD45−GFP+ CAR-immune cells (hCD45+GFP+) and hCD45+mCD45−GFP- non-transduced control immune cells (hCD45+GFP−) were sorted into CD3+CD19-CD33− T cells (CD3+), CD56+CD3− NK cells (CD56+), CD19+CD3−CD33− B cells (CD19+), and CD33+CD3−CD19− myeloid cells (CD33+) for RNA sequencing (RNA-seq). The GFP+ and GFP− populations were derived from the same CD34+ HSPC xenotransplanted graft and were, therefore, subjected to identical cell culture and transplantation conditions; this strategy enables internal comparison of CAR-associated effects.

Principal-component analysis (PCA) of RNA-seq data revealed clear separation of CAR-immune cells and non-transduced control immune cells for T cells, NK cells, and myeloid cells across all CAR designs (Figure 2B). NK cells that do not express a CAR appear to cluster closely with T cells, while NK cells expressing a CAR cluster closely with myeloid cells. This may reflect the lymphoid characteristics of the NK cells obtained by this model at steady state, which due to CAR expression or CAR activation may exhibit a transcriptional signature that aligns more closely with an innate or myeloid-like response. The relatively low abundance of NK cells may have also contributed to the heterogeneity in the NK cells observed. Notably, B cells displayed the least marked differences in transcriptional profiles upon antigen stimulation.

To determine the biological pathways underlying these responses, gene set enrichment analysis (GSEA) was conducted using Gene Ontology Biological Process (GOBP) reference gene sets, to assess coordinated transcriptional changes corresponding to validated

PB-CAR-NK cells (right), assessed in T cells and NK cells respectively, expressing the different CAR designs following antigen-specific activation. (G) Representative quantitative phase images of 4-1BB/ζ-T cell (left), 4-1BB/ζ-NK cell (middle), and 4-1BB/ζ-myeloid cell (right) interactions with A549-CD19−mKate target cells at a 1:1 E:T ratio (10× magnification); binding events are marked with white squares. Images are representative and shown for qualitative assessment; scale bar, 200 μm. (D and E) Genes (rows) shown represent the top positive, negative, or bidirectional contributors to pathway enrichment per CAR design (columns). For each CAR design, the top 20 positive and top 20 negative contributing genes based on FGSEA statistics from ranked log2FC in CAR-immune cells (GFP+) versus non-transduced control immune cells (GFP−) were selected and combined across CAR designs. Values represent the ranked gene-level statistic used for enrichment where positive values indicate enrichment toward CAR-immune cells (GFP+) and negative values indicate enrichment toward non-transduced control immune cells (GFP−) and are displayed using a symmetric color scale centered at zero (red, positive; blue, negative). GSEA was performed using Gene Ontology Biological Process (GOBP) gene sets from MSigDB v2025. RNA-seq data: $n = 1$ biological replicate. Schematic created with BioRender.com.

immune activation programs. Across at least two CAR designs, T cells showed weak but directionally consistent enrichment of T cells for "T cell Activation," NK cells for "Activation of Innate Immune Response," and myeloid cells for "Myeloid Leukocyte Activation" (Figure 2C). However, specific CAR design and immune cell type combinations did not always induce immune cell type-specific activation programs.

Only 2B4/ζ-B cells displayed enrichment for B cell-associated activation programs, whereas other CAR designs did not (Figure 2C), suggesting that canonical B cell activation is specific to the expressed CAR design. Assessment of gene-level contribution patterns to B cell activation enrichment across different CAR designs suggested that 2B4/ζ-B cells exhibited stronger positive contributors including chemokine and cytokine receptors, *CCR6* and *CXCR5*, and the class-switch recombination regulating transcription factor, *BATF*.[25] Negative contributors showed reduced negative influence, for example, genes with roles in B cell development like *MYB*[26] and *NFAM1*[27] (Figure 2D). 4-1BB/ζ-B cells also showed comparatively lower expression levels of positive contributors to enrichment compared with CD3ζ-B cells including *IL6*[28] and *SAMSN1*,[29] as well as greater negative contributions from *MYB*. This correlates with the lowest enrichment of 4-1BB/ζ-B cells with B cell activation programs (Figure 2C).

CD3ζ-myeloid cells similarly lacked enrichment for expected myeloid activation signatures despite immune activation of CD3ζ-T cells and CD3ζ-NK cells. Conversely, 4-1BB/ζ-myeloid cells and 2B4/ζ-myeloid cells displayed myeloid cell activation (Figure 2C). Gene-level contributions to myeloid leukocyte activation enrichment across different CAR designs highlighted that CD3ζ-myeloid cells showed differences compared with myeloid cells expressing the other CAR constructs (Figure 2E). This included weaker positive contributors, including for immune signaling components such as receptors for antigen detection, *CLEC4D*,[30] and adaptors to facilitate signaling complex assembly, *LAT*,[31] as well as for inflammatory regulators such as *MMP8*, which has been widely associated with a pro-inflammatory role.[32,33] However, simultaneously, genes involved in regulating the inflammatory state, such as *RORA*[34] and *TNF*[35] (Figure 2E), exhibited a relatively weaker negative contribution to enrichment in CD3ζ-myeloid cells. In conjunction, positive contributions to enrichment involved inflammatory mediators such as *IL6*, *IL18*, and *FN1*[36] at comparable levels to the other CAR-myeloid cells. At the gene level, these findings do not exhibit uniform inflammatory suppression but a partial or non-canonical activation state in CD3ζ-myeloid cells where selected inflammatory modules are upregulated in response to stimulation instead of a coordinated upregulation of all the pathways involved in myeloid cell activation. This may reflect the heterogeneous CD33+ myeloid compartment where cell type-specific transcriptional responses may be averaged during RNA-seq. Alternatively, these profiles may reflect differences in activation dynamics such as sustained CAR engagement where CAR-myeloid cells may have encountered endogenous human CD19+ B cells *in vivo* or during *ex vivo* culture, contributing to altered inflammatory and activation states.[37] Further gene contribution analysis suggests that there is an enrichment of inflammatory-associated programs in 4-1BB/ζ-compared with 2B4/ζ-myeloid cells such as *IL18*, *SPHK1*, *TLR4*, and *CAMP*. In contrast, 2B4/ζ-myeloid cells have greater contributions from *KIT*, *CLEC4D*, *GATA1*, and *BTK*, suggesting a distinct transcriptional state with greater developmental or signaling features despite a similar overall normalized enrichment score (NES). These results highlight that CAR-mediated canonical B cell activation is CAR design specific, while canonical myeloid cell activation occurs in some, but not all, CAR designs. In B cells and myeloid cells, different CAR designs may mediate partial activation states or influence the qualitative characteristics of the resulting activation state.

Interestingly, NK cells across CAR designs displayed positive enrichment for "Activation of Innate Immune Response" but negative enrichment for "Natural Killer Cell Activation" upon antigen stimulation (Figure S2D). This suggests that broader innate immune programs, shared across immune cell subsets, were upregulated upon antigen stimulation as opposed to coordinated NK cell-specific gene upregulation, and this is also supported by the clustering of activated CAR-NK cells with myeloid cells. This non-canonical activation upon antigen stimulation may reflect developmental differences of HSPC-derived NK cells arising in a humanized mouse model.

To further assess whether these transcriptional activation states reflect physiologically relevant effector programs, RNA-seq data were benchmarked against activated peripheral blood-derived CAR-T (PB-CAR-T) and CAR-NK (PB-CAR-NK) reference signatures. While PB-CAR-T cells were activated *in vitro* for 24 h,[6] PB-CAR-NK datasets were less frequently reported in the literature, and, therefore, a reference dataset investigating a prolonged 10-day *in vitro* activation was used.[38] Differences in transcriptional profiles may, therefore, reflect both the cellular origin or differences between early and sustained activation programs. GSEA using activation-associated transcriptional programs derived from peripheral blood CAR-T cells (4-1BB/ζ) demonstrated significant enrichment across HSPC-derived T cells across the different CAR designs, indicating concordance with established PB-CAR-T (4-1BB/ζ) cell activation states (Figure 2F). Interestingly, 2B4/ζ-T cells demonstrated the greatest enrichment, possibly reflecting possible innate-like characteristics of *in vivo* differentiated HSPC-derived T cells. Leading-edge analysis revealed that enrichment was driven by genes associated with proliferation, cell cycle progression, and immune activation in 2B4/ζ- and CD3ζ-T cells, whereas 4-1BB/ζ-T cells displayed a reduced proliferative signature alongside enrichment of specialized immune activation genes with innate-associated features such as *IRF8*[39] and *CD36*[40] (Figure S2E). Benchmarking against PB-CAR-NK (2B4/ζ) cell datasets demonstrated enrichment of activation-associated transcriptional programs in HSPC-derived NK cells. The magnitude of enrichment for CAR-mediated activation signatures exceeded that observed for canonical NK cell activation programs, suggesting that introduction of CAR signaling may induce additional or different transcriptional programs not typically present in

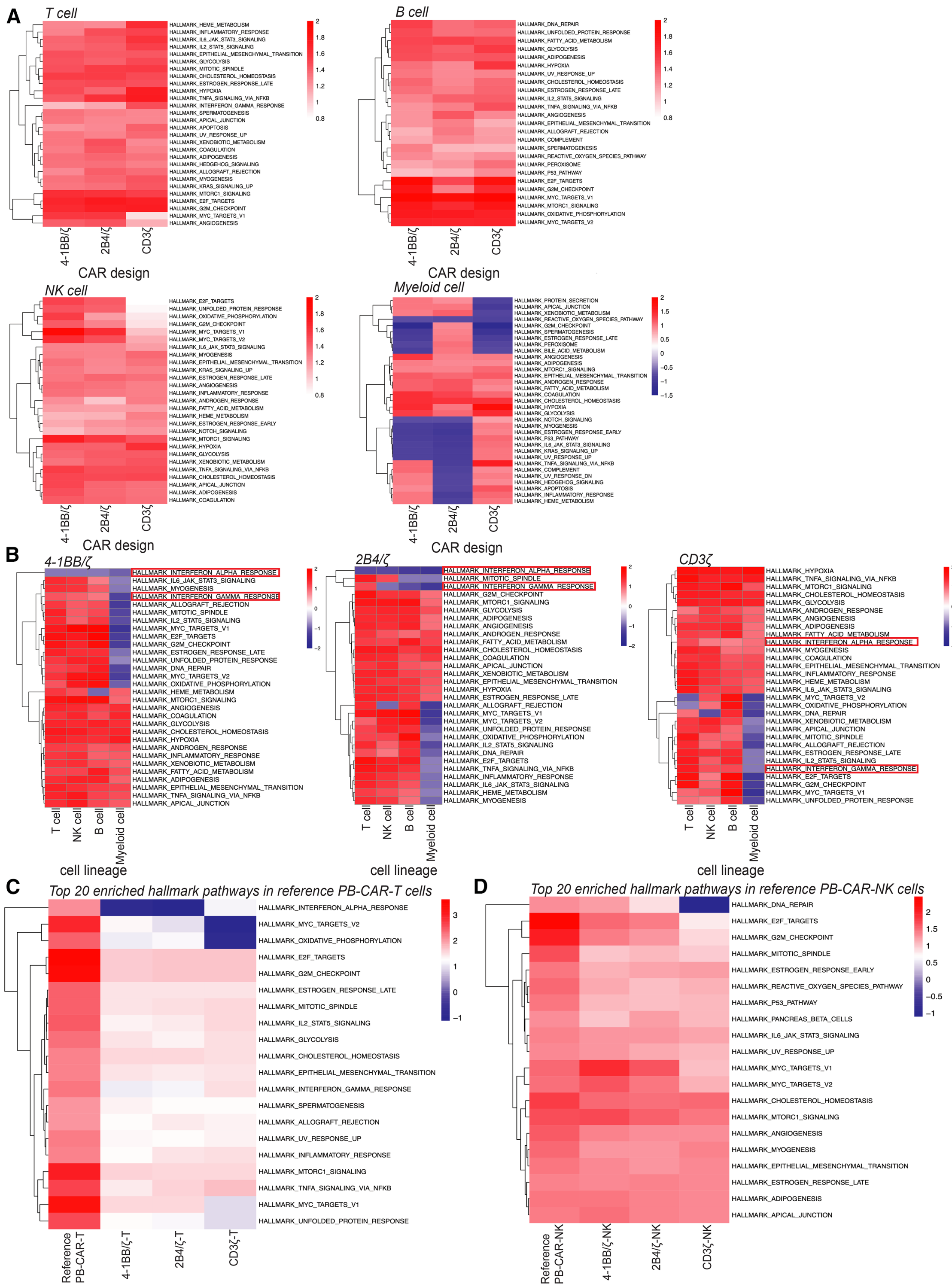

Figure 3. CAR design and immune cell type confer unique activation-induced gene expression identities

(A) Heatmaps showing NES for the top 20 enriched Hallmark pathways (rows) identified for each CAR design (columns) where the union across CAR designs is displayed for T cells (top left), NK cells (bottom left), B cells (top right), and myeloid cells (bottom right) across the three CAR designs, 4-1BB/ζ, 2B4/ζ, and CD3ζ. Color scales were fixed within each lineage for T cells, NK cells, and B cells where heatmaps were plotted using a positive NES scale (0.8–2; white to red), which covered the complete range of NES

(legend continued on next page)

activated NK cells (Figure 2F). Leading-edge analysis suggests that non-canonical NK cell genes such as those associated with myeloid lineages (e.g., *CD1C*, *LYZ*, *SIRPA*, *SPI1*, *CSF2RA*, and *CLEC7A*) were positive contributors across CAR designs, further suggesting a myeloid-like activation state or altered NK cell differentiation in the murine environment (Figure S2E).

To broadly assess functionality of CAR-expressing immune cells across the CAR designs, quantitative phase imaging provided qualitative evidence of CAR-T cells, CAR-NK cells, and CAR-myeloid cells physically interacting with A549-CD19−mKate target cells (Figure 2G; Videos S1, S2, and S3). Quantification of effector cell and target cell binding demonstrated that binding was detectable across GFP+ and GFP– immune populations; however, no consistent increased trend in binding frequency was observed in GFP+ immune cells relative to GFP– non-transduced control immune cells across immune cell type or CAR design (Figure S2F). To further investigate cytotoxic activity, a luminescence-based *in vitro* cytotoxicity assay was conducted where no clear *in vitro* cytotoxic activity of CAR-immune cells could be detected across the CAR designs (Figure S2G). Notably, a trend for increased IL6 production was detected in 4-1BB/ζ-T cells and 2B4/ζ-T cells following antigen activation, but not in CD3ζ-T cells, broadly consistent with *IL6* gene expression following antigen activation as identified by RNA-seq (Figure S2H). This lack of clear functional activity could possibly be due to incomplete functional maturation of cord-blood human HSPC-derived lymphoid cells in the murine environment, caused by disrupted lymphoid organ structure and absence of education by human antigens,[41] or the potentially limited terminal maturation of HSPC-derived myeloid cells as human cytokines such as IL3, IL6, and GM-CSF, that support myeloid cell differentiation, were absent.[20]

Together these results support that antigen stimulation, mediated by CARs, can induce transcriptional responses associated with immune activation despite limited effector function. Antigen engagement elicits canonical immune cell-specific activation transcriptional programs in select CAR design and immune cell type combinations: HSPC-derived 4-1BB/ζ-, 2B4/ζ-, CD3ζ-T cells, 4-1BB/ζ-, 2B4/ζ-myeloid cells, and 2B4/ζ-B cells. Other CAR design and immune cell combinations induce partial or non-canonical activation, for example, CD3ζ-myeloid cells or 4-1BB/ζ- and CD3ζ-B cells, where B cell activation appears to be particularly restricted to specific CAR designs.

Shared and distinct antigen-induced transcriptional programs emerge across CAR designs within immune cell types

As Figure 2 established that antigen stimulation induces immune activation across different CAR-immune cell types, next, the activation signatures conferred by different CAR designs were further investigated. Hallmark pathway analysis was performed on upregulated genes in CAR-immune cells compared with non-transduced control immune cells following antigen-specific activation, as determined by RNA-seq. Heatmaps of the top 20 enriched pathways across CAR designs within an immune cell type highlighted CAR design-specific signatures (Figure 3A).

In T cells, most activation-related pathways were similarly enriched across CARs; however, CD3ζ-T cells exhibited the greatest enrichment of the interferon-γ response (IFN-γ), a pathway with a multi-faceted role in tumor biology (Figure 3A), and under conditions of long-term stimulation can inhibit the maintenance and proliferative functions of stem-like T cells.[42,43] Unexpectedly, 4-1BB/ζ-T cells appeared to show a decreased inflammatory profile compared with 2B4/ζ- and CD3ζ-T cells as determined by the lower enrichment of “Inflammatory response” and “Allograft rejection” pathways as well as a reduced magnitude of enrichment for the “TNFα signaling via NF-κB” pathway despite the matched cell type with CAR design. This attenuated inflammatory response may reflect the maintenance of a comparatively more stem-like activated T cell state, alongside delayed acquisition of terminal effector programs. The retention of a less-differentiated state may also be supported by the enrichment of “Hedgehog signaling” observed in 4-1BB/ζ-T cells and 2B4/ζ-T cells, which has been reported to reduce T cell receptor signaling strength in mice[44] and regulate T cell development and differentiation.[45,46]

Despite 4-1BB/ζ-B cells and CD3ζ-B cells not showing induction of B cell-specific activation programs (Figure 2C), several activation-associated pathways, including cytokine response pathways such as “IL2-STAT5 signaling” and mitogenic signaling pathways such as “E2F targets” and “MYC targets,” were enriched (Figure 3A). This suggests antigen stimulation of 4-1BB/ζ-B cells and CD3ζ-B cells can elicit partial or limited immune cell-specific activation states defined by selective induction of activation pathways, whereas only 2B4/ζ-B cells supported the coordinated induction of canonical B cell-specific activation pathways.

values across the identified Hallmark pathways, whereas the heatmap for myeloid cells was plotted using a diverging scale (−1.5–2; blue to white to red) to visualize both negative and positive enrichment for the complete range of NES values across the identified Hallmark pathways. (B) Heatmaps showing the NES for the top 10 enriched Hallmark pathways (rows) following antigen stimulation, identified across cell lineages (columns) for each CAR design, 4-1BB/ζ (left), 2B4/ζ (middle), and CD3ζ (right). For each CAR design, the top 10 enriched Hallmark pathways were selected for each cell lineage, and the union across cell lineages is displayed. A fixed diverging color scale (−2 to 2; blue, white, red) was applied to visualize both positive and negative enrichment across cell lineages. (C) Heatmap of the top 20 positively enriched Hallmark pathways (rows) of a reference PB-CAR-T dataset, following 24-h CAR activation of peripheral blood (PB)-CAR-T cells (4-1BB/ζ). NES values for these pathways were compared with HSPC-derived 4-1BB/ζ-, 2B4/ζ-, and CD3ζ-T cells (columns). Color scale indicates NES (blue as negative; red as positive). (D) Heatmap of the top 20 positively enriched Hallmark pathways (rows) of a reference PB-CAR-NK dataset following a 10-day activation of PB-CAR-NK cells with an antigen-specific target cell line. NES values for these pathways were compared with HSPC-derived 4-1BB/ζ-, 2B4/ζ-, and CD3ζ-NK cells (columns). Color scale indicates NES (blue as negative; red as positive). RNA-seq data: *n* = 1 biological replicate. Hallmark pathways from Hallmark gene sets (MSigDB v.2024.1) were used.

In NK cells, comparatively weaker enrichment of multiple mitogenic pathways including "E2F targets," "MYC targets," and the "G2M checkpoint" was observed in CD3ζ-NK cells relative to the other CAR designs, suggesting that co-stimulatory signaling domains may enhance activation-associated transcriptional programs in these cells. However, as canonical NK cell activation characteristics were not clearly resolved previously, further interpretation of additional Hallmark pathway differences was not pursued.

Myeloid cells displayed the greatest divergence in hallmark pathway expression, with preferential activation of "TNFα signaling via NF-κB" in CD3ζ-myeloid cells (Figure 3A) despite a negative NES for the broader myeloid leukocyte activation gene set (Figure 2C), consistent with a partial activation that maintains pro-inflammatory characteristics. This was reflected in the greater positive contribution of NF-κB-dependent genes such as *IL6*[47] and genes that reinforce NF-κB signaling such as *MIF*[48] and *TGFB1*[49] to myeloid leukocyte activation enrichment in CD3ζ-myeloid cells (Figure 2E). In addition, a comparatively decreased enrichment of the "Inflammatory response" pathway in 2B4/ζ-myeloid cells combined with the reduced involvement of inflammatory-associated positively contributing genes for myeloid leukocyte activation gene set enrichment compared with 4-1BB/ζ-myeloid cells further supports a reduced inflammatory phenotype. CAR design-specific differences were also evident at the gene level (Figure S2I) with each cell type upregulating a set of shared activation-induced genes alongside distinct CAR design-specific genes.

To compare how CAR design shapes responses across different immune cell types, heatmaps of the top 10 enriched hallmark pathways across different immune cell types expressing the same CAR were examined (Figure 3B). This visualization enables additional comparison of antigen-specific signaling by immune cell type within a given CAR design. Primarily, this revealed that immune cell type-specific biology appears to be the most important determinant of activation-induced transcriptional changes at the pathway level, where the most pronounced differences are observed between myeloid and lymphoid lineages. This suggests a role for CAR design in the modulation and fine-tuning of activation-induced molecular phenotypes within lineage-constrained transcriptional programs.

"Interferon (IFN-α) response" showed lower enrichment in 4-1BB/ζ-immune cells and 2B4/ζ-immune cells compared with CD3ζ-immune cells, indicating CAR design-dependent differences in type I IFN signaling. "IFN-γ response" showed greater enrichment in NK cells and B cells expressing 4-1BB/ζ or CD3ζ compared with 2B4/ζ-expressing counterparts, suggesting that 4-1BB/ζ and CD3ζ stimulation induces greater IFN-γ-associated transcriptional programs across lymphoid cells, whereas 2B4/ζ stimulation restricts this to T cells. In particular, the coordinated enrichment of "IFN-α response" and "IFN-γ response" pathways in CD3ζ-B cells supports the observed partial B cell activation, where initial IFN-α stimulation has been reported to increase B cell survival and induce the expression of B cell costimulatory molecules such as CD86.[50] IFN-γ signaling may then act in concert, where IFN-γ stimulation of B cells after antigen exposure has been shown to promote proliferation.[50] Only "IFN-γ response" was enriched in 4-1BB/ζ-B cells and may contribute to the comparatively reduced enrichment of B cell activation. In contrast, enrichment of "IFN-α response" and "IFN-γ response" pathways were not observed in 2B4/ζ-B cells despite the induction of canonical B cell activation programs, suggesting that 2B4/ζ-mediated B cell activation may arise through distinct downstream signaling processes independent of IFN-associated transcriptional responses. This indicates that CAR architecture can shape distinct activation-associated transcriptional states in B cells characterized by different downstream signaling programs.

At the pathway level, when benchmarked against reference PB-CAR-T[6] and PB-CAR-NK[38] datasets, enrichment patterns were broadly conserved in HSPC-derived immune cells across the CAR designs, although consistently observed at reduced magnitude (Figures 3C and 3D). In HSPC-derived CAR-T cells, the most strongly enriched Hallmark pathways in PB-CAR-T cells showed similar patterns of enrichment across CAR designs but with lower intensity, including "E2F targets" and "mTORC1 signaling," suggesting a reduced capacity to mount robust effector programs, consistent with incomplete terminal effector maturation in this model. This was particularly evident in CD3ζ-T cells, which demonstrated reduced enrichment of MYC target pathways. In HSPC-derived NK cells, pathway enrichment patterns were likewise broadly conserved relative to PB-CAR-NK datasets, although the reduction in magnitude was not as pronounced as for HSPC-derived T cells. Nevertheless, CD3ζ-NK cells showed lower enrichment of proliferation-associated pathways including "DNA repair," "G2M checkpoint," and "Mitotic spindle" (Figure 3D). These findings indicate that HSPC-derived CAR-T and CAR-NK cells arising from a humanized mouse model can recapitulate key transcriptional features of PB-CAR activation programs, albeit at a reduced intensity, which may be below the thresholds required for the execution of effector functionality.

Collectively, these findings reveal that CAR design and effector immune cell type together shape antigen-specific activation programs, engaging a core set of pro-proliferative and immune-response pathways, while conferring distinct, CAR design- and immune cell type-specific transcriptional states.

InTraSeq single-cell profiling reveals CAR design-specific signaling across diverse immune cells

To determine whether these transcriptional programs correspond to early intracellular signaling events, single-cell InTraSeq profiling[51] was performed on 4-1BB/ζ-immune cells and 2B4/ζ-immune cells following 10 min antigen-specific activation (Figure 4A). This approach quantifies, in single cells, signaling protein and post-translational modification (PTM) states simultaneously with transcriptomic profiles. Activation of signaling pathway phosphorylation was validated in PB-4-1BB/ζ-T cells by western blot following a 10-min antigen stimulation (Figure S3). CD3ζ-immune cells were excluded due to low engraftment of CD3ζ-HSPCs in the transplant

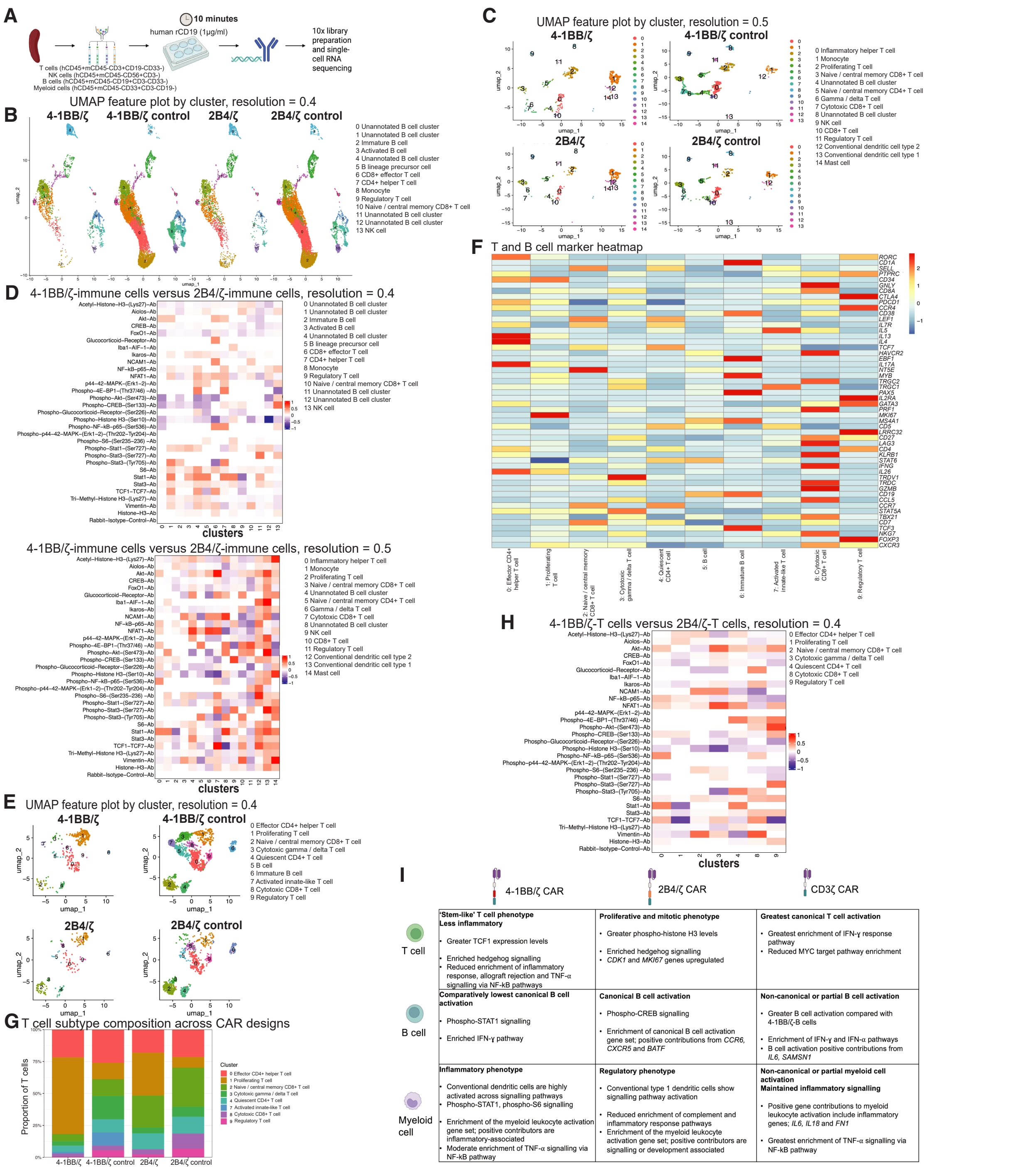

Figure 4. Single-cell analysis across diverse immune cells reveals CAR design-specific signaling pathway activation

(A) Schematic of the experimental workflow. Spleen-isolated, 4-1BB/ζ- and 2B4/ζ-immune cells (GFP+) and non-transduced control immune cells (GFP−) arising from the respective xenotransplants were sorted into T cells, NK cells, B cells, and myeloid cells and subjected to *ex vivo* antigen stimulation and subsequent analysis by InTraSeq. (B) UMAP feature plots of CAR-immune cells across 4-1BB/ζ-immune cells (GFP+) (far left), 4-1BB/ζ-control immune cells (GFP−) (center left), 2B4/ζ-immune cells (GFP+) (center right), and 2B4/ζ-control immune cells (GFP−) (far right) when resolved at 0.4. Cells are colored by cluster identity, and clusters are labeled from 0 to 13. (C) UMAP feature plots of the T cell, NK cell, and myeloid cell compartments, clusters 6, 7, 8, 9, 10, and 13 from (B) resolved at 0.5 for 4-1BB/ζ-immune cells (GFP+) (top left), 4-1BB/ζ-control immune cells (GFP−) (top right), 2B4/ζ-immune cells (GFP+) (bottom left), and 2B4/ζ-control immune cells (GFP−) (bottom right). Cells are colored by cluster identity, and clusters are labeled from 0 to 14. (D) Heatmaps of CLR-normalized antibody-derived tag (ADT) signaling features showing median differences between 4-1BB/ζ-immune cells (GFP+) and

(legend continued on next page)

cohort for this investigation, resulting in insufficient human CAR-immune cell recovery. Low- and high-resolution clustering, driven by differentially expressed genes per cluster and lineage-specific marker gene expression from single-cell RNA-seq data (Figures 4B and 4C; Figures S4A and S4B), identified T cell, B cell, and dendritic cell subsets and monocytes in CAR-expressing and non-transduced control samples with an additional mast cell cluster in CAR-immune cells, confirming the presence of diverse, multilineage immune cells derived from 4-1BB/ζ- and 2B4/ζ-HSPCs at single-cell resolution (Figures 4B and 4C).

In addition to enabling analysis of early signaling events, the transcriptomic component of the InTraSeq dataset allowed us to assess the lineage identity and developmental maturity of HSPC-derived immune cells. As hematopoiesis occurred *in vivo* within a humanized mouse model, we aimed to assess the physiological relevance and developmental maturity of HSPC-derived immune cells by comparison of their transcriptional profiles with a reference human peripheral blood mononuclear cell (PBMC) dataset.[52] Label transfer analysis of combined HSPC-derived immune cells across conditions, using reference PBMC clusters annotated by the top differentially expressed genes per cluster, demonstrated that myeloid and lymphoid lineages such as monocytes, B cells, and T cells could be broadly assigned (Figures S4C and 4D). PBMCs exhibited defined mature states as revealed by B cell and T cell clusters in the HSPC-derived immune cell dataset that closely aligned to naive B cell and T cell transcriptional states in the PBMC dataset. Some overlap between NK cells with predicted T cell populations was observed, suggesting that the T cells in this system may exhibit a more innate, NK cell-like phenotype. In addition, predicted NK cell populations showed partial overlap with B cell clusters, which may reflect some artificial development in the lymphoid compartment arising in this model. Additional label transfer analysis using reference cord blood-derived mononuclear cells[53–55] (CBMCs) was conducted, which may more closely reflect the developmental state of the HSPC-derived immune cells from this system. As the *in vivo* differentiated HSPC-derived immune cells consisted of hCD45+ and human lineage marker populations, CBMC populations representing erythroid lineages, CD34+ progenitor cells, and murine 3T3 fibroblast cells were excluded from the comparison prior to label transfer. The annotation of T cells, B cells, and dendritic cells was largely consistent with the original cluster annotation (Figure 4B) and label transfer using a PBMC reference (Figure S4D). Cluster 5, annotated as B lineage precursor cells (Figure 4B), showed inconsistency across reference datasets, annotated as cytotoxic NK cells and as cycling cells (Figure S4D) when using reference PBMC and CBMC datasets, respectively, which may support a proliferative, immature lymphoid population whose transcriptional identity remains unresolved. This ambiguity may reflect an intermediate developmental lymphoid population arising in the humanized mouse model that is not fully represented during physiological human immune cell differentiation. In addition, cluster 8, originally annotated as monocytes (Figure 4B), aligned with monocyte populations in the PBMC reference dataset but did not map to the CBMC monocyte reference population and may indicate that at a transcriptional level, *in vivo* differentiated HSPC-derived monocytes more closely resemble PBMC-derived monocytes than CBMC-derived monocytes.

To further assess T cell developmental states, label transfer analysis of *in vivo* differentiated HSPC-derived T cells from this system was performed using reference human thymocyte[56] data (Figure S4D). Although the T cell phenotype is influenced by the tissue and microenvironment context, this approach enabled comparison across key T cell developmental stages. Broad agreement was observed between T cell proliferation state in both datasets with proliferating T cell clusters aligning with proliferative thymocyte populations. While clusters annotated as naive/central memory CD8+ T cells, cytotoxic gamma/delta T cells, and quiescent CD4+ T cells in the original dataset (Figure 4E) predominantly mapped to mature single-positive T-like cell states, several other T cell clusters aligned with developmental thymocyte populations, suggesting retention of immature or developmental transcriptional features. In addition, originally annotated cytotoxic CD8+ T cells and regulatory T cells displayed innate-like characteristics that may reflect altered T cell differentiation within the humanized mouse environment. Cluster 5, originally annotated as a B cell population with comparatively higher expression of *CD19* and *MS4A1* (Figures 4E and 4F) showed the highest similarity to double-positive thymocytes in the reference dataset, and this was interpreted cautiously, as this discrepancy could reflect overlapping developmental lymphoid transcriptional programs, limitations of label transfer analysis, or altered lymphoid development within the humanized mouse system. Next, integrated uniform manifold approximation and projection (UMAP) analysis with the reference human thymocyte and PBMC-derived T cells (Figures S4C and S4E) allowed

2B4/ζ-immune cells (GFP+) across all the CAR-immune cell clusters from (B) resolved at 0.4 (top). Heatmaps of CLR-normalized ADT signaling features showing median differences between 4-1BB/ζ-immune cells (GFP+) and 2B4/ζ-immune cells (GFP+) across clusters 6, 7, 8, 9, 10, and 13 from (B) resolved at 0.5 (bottom). (E) UMAP feature plots of the T cell compartment (clusters 6, 7, 9, and 10 from B), re-clustered at a resolution of 0.4 for 4-1BB/ζ-T cells (GFP+) (top left), 4-1BB/ζ-control T cells (GFP−) (top right), 2B4/ζ-T cells (GFP+) (bottom left), and 2B4/ζ-control T cells (GFP−) (bottom right). (F) Heatmap of scaled average single-cell RNA expression of T cell and B cell lineage and functional marker genes across T cell clusters (clusters 6, 7, 9, and 10 from B, resolution 0.4). (G) Stacked bar plots showing the proportional distribution of T cell clusters (clusters 6, 7, 9, and 10 from B, resolution 0.4 with cluster 5 and 6 excluded, across conditions: 4-1BB/ζ-T cells (GFP+), 4-1BB/ζ-control T cells (GFP−), 2B4/ζ-T cells (GFP+), and 2B4/ζ-control T cells (GFP−). (H) Heatmap of CLR-normalized ADT signaling features showing median differences across T cell clusters (clusters 6, 7, 9, and 10 from B, resolution 0.4), between 4-1BB/ζ-T cells (GFP+) and 2B4/ζ-T cells (GFP+). (I) Summary comparative schematic integrating transcriptional and signaling phenotypes across CAR designs (columns) and immune cell type (rows) across T cells, B cells, and myeloid cells; NK cells have been excluded due to non-canonical identity and low abundance. Annotations summarize dominant signaling features following antigen stimulation and transcriptional programs 24 h after activation. (D and H) Rows represent signaling proteins and post-translational modifications, and columns represent clusters. Rows and columns are ordered alphabetically and numerically consistently across heatmaps. The color scale is symmetric around zero. InTraSeq samples: $n = 1$ biological replicate. Schematic created with BioRender.com.

for comparative assessment of developmental and mature T cell states. Except for cluster 1, which transcriptionally resembled mitotic thymocytes, the HSPC-derived T cells clustered predominantly closer to PBMC-derived T cells including effector populations such as cytotoxic CD8+ T cells and activated CD4+ T cells. Analysis of T cell lineage markers across the nearby thymocyte and HSPC-derived T cell clusters revealed that there was comparatively lower expression of early T cell developmental (*CD27* and *CD7*) and proliferative markers (*AURKB*, *PCNA*, and *MKI67*). Some canonical T cell markers such as *CD3D* and *CD3E* had lower expression in the HSPC-derived T cells, while other developmental markers such as *CD5* retained expression, suggesting some differences from physiological T cell development (Figure S4F). Together, these analyses indicate that while the humanized mouse model for *in vivo* differentiation lacks a fully physiological microenvironment for T cell development, which could reflect the developmental or innate-like characteristics of the produced T cells, the resulting HSPC-derived T cells, nevertheless, acquire transcriptional features that broadly resemble human PBMC-derived T cells and are not restricted to an exclusively immature or artificial phenotype.

Across this diverse multilineage cell landscape, centered log-ratio (CLR)-normalized signaling protein expression levels and PTM levels revealed that antigen stimulation induced widespread molecular changes beyond CAR design-matched cell types (Figures S4G and S4H). This indicates that CAR activation can propagate signaling responses across the broader immune cell population. In 4-1BB/ζ-immune cells, higher phosphorylated-4EBP1 (p4E-BP1), pSTAT3-Ser727, and pSTAT1-Ser727 signals were observed across multiple immune subsets compared with non-transduced control cells. 2B4/ζ-immune cell stimulation was associated with higher p4E-BP1 and pSTAT3-Tyr705 levels across several populations compared with non-transduced control cells. Some outputs such as pNF-κB exhibited cell type-dependent patterns across both CARs (Figure S4G). At higher resolution, conventional dendritic cell type 1 emerged as a key responsive population for both CAR designs, while 4-1BB/ζ also strongly activated conventional dendritic cell type 2 (Figure S4H). These observations indicate CAR engagement triggers broad and heterogeneous signaling responses across the multilineage immune landscape, extending beyond CAR design and cell type-matched populations.

To investigate activation biases imposed by CAR architecture, CLR-normalized signaling profiles of 4-1BB/ζ-immune cells and 2B4/ζ-immune cells were compared (Figure 4D). Within the same cell type, CAR design mediated distinct signaling preferences. 4-1BB/ζ-activated B cells displayed higher levels of pSTAT1-Ser727 (Figure 4D; Figure S4I) whereas 2B4/ζ-activated B cells showed higher levels of pCREB (Figure 4D). This signaling divergence is consistent with transcriptional changes observed by RNA-seq, where 4-1BB/ζ-B cells demonstrated activation-induced enrichment of the hallmark IFN-γ pathway, whereas 2B4/ζ-B cells did not. Previous reports have demonstrated that the increase in activated pSTAT1-Ser727 in B cells when stimulated with IFN-γ[57] and loss of pSTAT1-Ser727 phosphorylation in fibroblasts led to a concomitant decrease in specific downstream IFN-γ target gene expression.[58] Alternatively, in 2B4/ζ-activated B cells, higher levels of pCREB has been linked to the expression of AP1 transcription factors in activated B cells,[59] which may also be supported by the greatest positive contribution of *BATF,* an AP1 family member (Figure 2D),[60] to B cell activation enrichment. AP1 signaling facilitates activation-induced proliferation and survival of activated B cells,[52] which may mediate the canonical B cell activation observed, supporting the earlier observation that distinct CAR architectures in B cells induce activation-associated phenotypes through different downstream signaling programs. This suggests a link between CAR-dependent signaling biases and downstream transcriptional programs.

In the myeloid compartment, across monocytes and dendritic cell clusters, higher levels of pSTAT1-Ser727 and pS6 levels could be observed for 4-1BB/ζ-expressing cells compared with those expressing 2B4/ζ. STAT1 has been documented to be a key inflammatory mediator in macrophages in a murine model of traumatic brain injury.[61] In addition, mTORC1, an upstream activator of S6 kinase phosphorylation has been reported to be an important driver of systemic inflammation in a murine model of Still's disease, where mTORC1 activation was observed in monocytes from this inflammatory disease model and mTORC1 inhibition resulted in reduced proliferation of inflammatory monocytes.[62] This implication of STAT1 and mTORC1-S6 signaling in specific models of pathology suggests that the increased activation of these pathways by antigen stimulation of 4-1BB/ζ-myeloid cells may facilitate the corresponding induction of the observed pro-inflammatory transcriptional programs. These pathways are not enriched in 2B4/ζ-myeloid cells that exhibit lower levels of STAT1 and mTORC1-S6 (Figure 4D) phosphorylation.

Additionally, across several CD4+ and CD8+ T cell clusters such as 2B4/ζ-inflammatory helper T cells, 2B4/ζ-naive/central memory CD8+ T cells, and 2B4/ζ-naive/central memory CD4+ T cells, higher levels of p-histone-H3 were observed (Figure 4D). Although G2M checkpoint and E2F targets were both highly enriched in 4-1BB/ζ- and 2B4/ζ- T cells, this also corresponded with a greater upregulation of mitotic genes such as *CDK1* and *MKI67* in 2B4/ζ-T cells 24 h after antigen stimulation (Figure S2I), which may suggest a more pronounced proliferating phenotype mediated by 2B4/ζ. In contrast, 4-1BB/ζ-CD8+ cytotoxic T cells showed a significant upregulation of TCF1, a key transcriptional regulator in the maintenance of the stem-like T cell compartment, compared with 2B4/ζ-T cells (Figure 4D). Although this expression is unlikely to result from *ex vivo* antigen stimulation, this may suggest maintenance of a stem-like state facilitated by this CAR design possibly by *in vivo* antigen stimulation when interacting with human CD19+ B cells produced by the human CD34+ xenotransplant or through CAR-dependent tonic signaling. Upon activation, this is associated with a decreased inflammatory transcriptional signature compared with 2B4/ζ-T cells, in agreement with a greater stem-like T cell phenotype (Figure 3A).

Next, in greater detail, we specifically examined T cell subsets expressing either 4-1BB/ζ or 2B4/ζ and their respective non-transduced

control T cells that were isolated and re-clustered from the global clustering (Figure 4E). Cluster annotation was similarly conducted by assessing differentially expressed genes for each cluster as well as investigating comparative expression of key T cell state and lineage marker genes (Figure 4F). Diverse T cell states including activated and quiescent T cells as well as subsets of CD4+ and CD8+ T cells could be identified. Activated innate-like T cells, cluster 7, exhibiting a comparatively high expression for *IL5* and *TRGC1*, supporting a γδ-T cell population,[63] were only present in the 4-1BB/ζ-xenotransplant and were not observed in the 2B4/ζ-xenotransplant, possibly reflecting human HSPC heterogeneity in cell fate potential. Two contaminant B cell clusters (clusters 5 and 6) were identified and removed for the downstream analysis. A comparison of the proportion of T cell subsets in each condition revealed that cells expressing the CAR had an increased proportion of proliferating T cells, which may reflect *in vivo* activation through contact with CD19+ human B cells in the spleen or CAR-dependent tonic signaling, which may have implications for the acquisition of exhausted T cell transcriptional programs at later time points (Figure 4G).[64]

A comparison of the CLR-normalized medians of signaling proteins and their PTMs between T cell subsets expressing 4-1BB/ζ or 2B4/ζ CARs and their respective controls reveals that in both cases regulatory T cells are a particularly responsive subset to antigen-induced activation (Figure 4H; Figure S4J). In addition, proliferating T cells exhibit greater signaling activation by the 2B4/ζ receptor compared with the 4-1BB/ζ receptor, whereas cytotoxic CD8+ T cells show greater signaling activation by the 4-1BB/ζ-receptor. 4-1BB/ζ-CD8+ cytotoxic T cells demonstrate greater levels of pAkt, p4E-BP1 and pNF-κB compared with those expressing the 2B4/ζ-receptor (Figure 4H; Figure S4J), reported to be major mediators of CD8+ T cell activation.[65–67] In particular, NF-κB signaling has been implicated in the survival of T cells following antigen stimulation[68] with a dependence on NF-κB activation by TRAF proteins, which themselves have NF-κB-dependent expression.[69] While shifts in the balance between NF-κB-responsive pro- and anti-apoptotic factor expression levels are important for the final cell state outcome,[70] RNA-seq data after 24 h of antigen stimulation demonstrate a trend where anti-apoptotic genes have increased expression in 4-1BB/ζ-T cells such as *TRAF2* and *BCL2* and pro-apoptotic genes such as *BCL2L14* show a lower expression level. However, there are exceptions such as the higher expression level of *BCL2A1* in 2B4/ζ-T cells, which may reflect the complex regulation of target genes downstream of the NF-κB pathway (Figure S4K).[71–74]

These findings enable the development of a comparative framework summarizing the dominant molecular phenotypes associated with each CAR design across T cells, B cells, and myeloid cells (Figure 4I). NK cells were not included in this framework due to their low abundance and non-canonical transcriptional phenotype, which limited robust and representative categorization at the same level as other immune lineages. Overall, these exploratory results indicate that both CAR design and immune cell type shape immediate signaling states, producing receptor- and cell type-specific activation programs, that can be linked to downstream transcriptional outcomes.

DISCUSSION

Here, we leveraged a chemically defined *ex vivo* HSC culture platform[2] to enable the generation of multilineage CAR-immune cells for systematic investigation of CAR signaling across diverse immune cell types. A key strength of this approach is the simultaneous generation of diverse CAR-expressing immune cells from *ex vivo* CAR-engineered HSPCs, providing a platform for the systematic comparison of CAR design-dependent modulation of activation phenotypes in different immune cells.

In this NOG-w41 xenotransplantation model, effector immune cell populations including T cells, NK cells, B cells and myeloid cells could be detected in the spleen and bone marrow. Stimulation of the different CARs induced activation-associated transcriptional changes in T cells, NK cells, and myeloid cells, whereas canonical B cell-specific activation was only observed in 2B4/ζ-B cells. Specifically, antigen engagement did not uniformly trigger canonical immune cell-specific activation, instead driving partial or non-canonical activation states depending on the CAR design and immune cell type pairing. CD3ζ-myeloid cells did not exhibit enrichment of a complete myeloid leukocyte activation signature following antigen stimulation, whereas 4-1BB/ζ-myeloid cells displayed a more inflammatory phenotype compared with 2B4/ζ-myeloid cells. Previous CAR-myeloid cell studies have demonstrated that CD3ζ-containing CARs can support myeloid activation and effector function,[10,75,76] whereas the consequences of 4-1BB signaling in myeloid cells remain less clearly defined with context-dependent effects, for example, on macrophage activation and polarization.[77–80] Although the majority of CAR-myeloid cell studies investigate CAR-macrophages specifically, several of these platforms have incorporated myeloid-adapted signaling domains, including FcRγ-, Megf10-, MyD88-, and TLR-associated signaling domains, to additionally enhance immune activation profiles.[18,81] Pathway analysis further revealed that immune cell type-specific biology appears to be the dominant determinant of activation-induced transcriptional changes, suggesting that CAR design can modulate activation-associated molecular phenotypes within lineage-constrained cell circuitry. Within each immune cell subset, CARs drove both shared activation programs and design-specific transcriptional responses. In particular, inflammatory responses differed, for example, lower enrichment of "Inflammatory response" in CD3ζ-T cells or greater enrichment of "TNFα signaling via NF-κB" in CD3ζ-myeloid cells, relative to their counterparts expressing the other CAR designs. These findings indicate that CAR design can shape distinct activation-induced characteristics across matched and mismatched CAR-immune cell type combinations. Importantly, single-cell signaling protein and PTM profiling further supported that signaling biases reflect both receptor design and intrinsic cell circuitry, with links to observed downstream transcriptional responses. Collectively, these findings show that CAR design equips a diverse set of immune cells with unique antigen-specific activation identities.

To date, CAR-engineering studies have largely focused on limited effector immune cell populations.[82–84] While powerful, these reductionist approaches capture only a subset of possible activation signatures.[85] In contrast, the *in vivo* HSPC differentiation approach here allows probing of a broader immune landscape including dendritic cells and mast cells. Combined with CAR-engineering, this platform allows systematic evaluation of how distinct CAR designs influence molecular activation across multiple immune cell types. While previous studies have compared CAR responses across different immune cell types using a common CAR construct,[15] the present study evaluates previously validated, immune cell type-optimized CAR designs within a unified experimental framework, enabling assessment for how CAR design and immune cell type shape activation programs. This is enabled by the common HSPC origin, their CAR-engineering, *ex vivo* expansion, and multilineage differentiation, allowing direct cross-lineage comparisons of CAR-dependent activation states including summary phenotypes, expected activation-associated signaling transduction events, and transcriptional pathways. Although the murine developmental environment is not physiological, this model supports multilineage differentiation from the same *in vivo* environment enabling the simultaneous generation of an immune cell system for dissecting CAR-driven molecular responses. An additional distinction from standard CAR-engineering using HSPC-derived, rather than peripheral blood-derived, effector immune cells allows the investigation of cell types that are difficult to engineer at mature stages, such as conventional dendritic cells, which are challenging to isolate from peripheral blood sources at sufficient yield.[86] As regenerative cell therapies often exhibit altered phenotypes and functionality,[87–90] benchmarking these findings against standard, clinically relevant peripheral blood-derived systems will help determine how transcriptional and signaling programs in HSPC-derived immune cells translate to mature immune cells.

Despite the advantages of this system, several limitations must be acknowledged. A potential source of variability in the assessment of antigen-induced signaling and transcriptional activation is the CAR copy number present in the genome per cell across CAR designs, due to varying lentiviral transduction efficiencies. As a result, variation in the number of CAR molecules per cell, mediating the antigen response, could influence downstream signaling strength and affect the characteristics[91] of specific activated CAR design and immune cell type combinations such as specific hallmark pathway enrichment. Although quantitative assessment of CAR vector copy number was not conducted in this system, low human HSPC transduction efficiency in a comparable range[92] was observed across the CAR designs. In addition, immune activation at the transcriptional level showed a weak but broadly consistent trend across different CAR designs, suggesting that the observed signaling and transcriptional programs are unlikely to be solely explained by differences in CAR transgene copy number.

The xenotransplantation environment introduces lineage biases such as disproportionately elevated B and myeloid cell frequencies in the spleen and particularly in the bone marrow, creating technical challenges for the downstream analyses. At 15 and 16 weeks post-transplantation, only low yields of T cells and NK cells could be detected, respectively, in the peripheral blood and bone marrow, limiting the analogous investigation of activation-induced signaling and transcriptional changes of HSPC-derived multilineage CAR-immune cells from these sources. CAR-immune cells also displayed alternative lineage distributions when compared with non-transduced control immune cells generated in the same environment. In addition, low CD3ζ-HSPC engraftment in particular transplant cohorts prevented InTraSeq profiling of the resulting CD3ζ-immune cells and no T cells could be detected in the bone marrow of CD3ζ-HSPC-transplanted mice at the experimental endpoint. These observations suggest that CAR expression at the HSPC stage may alter functional characteristics such as engraftment, self-renewal, and differentiation trajectories and may additionally influence lineage-specific tissue distribution or persistence *in vivo*. However, these effects were not directly assessed in this investigation and may require future study. Accordingly, we cannot exclude the possibility that differences in differentiation arising from CAR expression contribute, at least in part, to the transcriptional differences observed in CAR-immune cells. However, single-cell analyses indicate that spleen-derived T cells generated in this model follow broadly similar developmental trajectories to their non-CAR counterparts, supporting the interpretation that these cells adopt comparable lineage identities.

Moreover, functional cytotoxic activity was not observed *in vitro* and is consistent with the presence and persistence of human CD19+ B cells in xenotransplanted NOG-w41 mice. While this system allowed assessment of molecular activation phenotypes, we were unable to robustly assess functional outcomes such as cytotoxicity, phagocytosis, proliferation, or persistence of each CAR-immune cell. This is consistent with known limitations of the NOG-w41 humanized mouse model, which supports engraftment and development of phenotypic immune cell populations but may not fully recapitulate terminal immune maturation across myeloid and lymphoid lineages, likely due to the lack of key human developmental cues, cytokines, and human antigen presentation.[20,93–95] Benchmarking against PB-CAR-T and PB-CAR-NK cells demonstrates antigen-dependent enrichment of activation pathways at the transcriptional level but with reduced magnitude. When considered with the absence of robust effector function, this suggests conservation of core molecular activation programs despite attenuated functional output, which may reflect transcriptional states that do not reach thresholds required for full effector functionality. Consequently, it remains unclear how these activation-induced molecular changes translate to effector functionality and thus, more broadly, to therapeutic relevance. In addition, as this study aimed to systematically evaluate previously validated, cell type-specific CAR designs, future work could focus on the mechanistic basis of individual CAR domain contributions through domain-swapping or inhibition approaches.

Finally, we emphasize the exploratory nature of this study. As xenotransplantation cohorts were generated using commercially sourced pooled human cord blood-derived CD34+ HSPCs from independent

manufacturing lots, potential lot-related and donor-related variability was not explicitly assessed in this study. Analyses were performed once per cell type per CAR, and thus the transcriptomic and signaling trends serve as a proof of concept rather than statistically definitive. Future investigations incorporating replicate sequencing across additional donor sources and transplantation cohorts will be critical to enable a more rigorous statistical evaluation of these responses.

When considered in the context of these limitations, our findings support a model in which CAR design and immune cell type act as modular components that determine molecular activation identity. This concept suggests that CARs and immune cells can be paired to generate tailored, antigen-specific activation phenotypes. These findings hold promise for future applications of rational, cell-specific CAR-engineering to produce tailored activation-induced phenotypes whereby distinct immune cell types are conferred with complementary activation-induced phenotypes by different CAR designs. Importantly, this framework highlights the potential of CAR-engineering for non-canonical cell types such as dendritic cells or mast cells ,which may exhibit complementary activation-induced activity alongside CAR-T cell or CAR-NK cell therapies. By defining the specific activation characteristics mediated by different CAR designs across diverse immune cell types, this model may inform the constitution of combinatorial CAR-immune cell strategies, in which complementary CAR-dependent activation programs are combined at defined ratios to optimize a unified CAR-immune cell therapy. While systematically different activation profiles that depend on immune cell type and CAR design were observed in an HSPC-derived system, these principles may have implications for emerging CAR therapy strategies that involve diverse immune cell types including CAR-engineering of PBMCs[96] and *in vivo* CAR-engineering of HSCs.[97] Some immune cell types may be more sensitive to the configuration of the CAR expressed, exhibiting an attenuated immune activation or inflammatory transcriptional response, whereas expression of specific CAR designs in these cell types may enhance pro-inflammatory responses. Together, this highlights the importance of considering heterogeneous activation responses mediated by different CAR designs when developing multilineage CAR-immune cell therapies. In summary, this approach lays the foundation for future studies validating effector functionality of validated CAR designs across diverse immune cell types and systematically tuning CAR design and effector cell identity to engineer next-generation CAR-immune cell therapies.

MATERIALS AND METHODS

Cell culture

HEK293T cells were used for lentiviral production and maintained in Dulbecco's Modified Eagle Medium (DMEM) supplemented with 10% fetal bovine serum (FBS) and 1% penicillin-streptomycin (P/S) (Wako, Osaka, Japan, #: 168-23191). Cells were passaged every 2–3 days using 0.05% (w/v) trypsin solution. A549 cells were transduced with lentiviral vectors expressing human CD19 (pCDH-CMV-CD19 puro) (Addgene, Watertown, Massachusetts, #196634) and mKate fluorescent protein. Cells were cultured in DMEM supplemented with 10% FBS and 1% P/S and passaged using 0.05% (w/v) trypsin every 2–3 days. A monoclonal A549-CD19−mKate target cell line was generated by single-cell index sorting followed by clonal expansion. A549 cells were transduced with lentiviral vectors expressing human CD19 (pCDH-CMV-CD19 puro) (Addgene, Watertown, Massachusetts, #196634) and akaluc-modified luciferase. Cells were cultured in DMEM supplemented with 10% FBS and 1% P/S and passaged using 0.05% (w/v) trypsin every 2–3 days. A monoclonal A549-CD19−akaluc target cell line was generated by single-cell index sorting followed by clonal expansion.

PBMCs were isolated from a healthy volunteer (in accordance with University of Tokyo guidelines and approved by the Ethics Committee, approval no: 2026-18-0616) using density gradient centrifugation (Lymphoprep, STEMCELL Technologies, Vancouver, Canada, #07851/07861). Cells were cultured in Roswell Park Memorial Institute (RPMI)-1640 medium (Wako, Osaka, Japan, #189-02145) supplemented with 10% FBS, 1% P/S, 5 ng/mL recombinant human interleukin-7 (PeproTech, Cranbury, New Jersey, #200-07-10UG), and 5 ng/mL recombinant human interleukin-15 (PGI Proteintech Group Inc, Rosemont, Illinois, #HZ-1323). To increase T cell abundance prior to isolation, PBMCs were activated using a T cell Activation/Expansion Kit (Miltenyi Biotec, Bergisch Gladbach, Germany, #130-091-441), according to the manufacturer's instructions, with medium changes every 3 days.

Pooled human cord-blood CD34+ HSPCs were purchased from CGT Global (CGT Global, Folsom, California, catalog #: CBP3400.5C, lot #: 2407020027, 2407030045, and 2411250111). Cells were thawed and washed twice in 0.01% Soluplus in PBS. CD34+ cells were cultured in Iscove's Modified Dulbecco's Medium L-glutamine(−) (IMDM) (Sigma-Aldrich, St. Louis, Missouri, catalog #: I3390-500mL) supplemented with 1% P/S, 1x insulin-transferrin-selenium-X (ITS-X) (Gibco, Grand Island, New York, #51500-056), 0.01% Soluplus (BASF, Ludwigshafen, Germany, lot #: 38830216KO), 5 μM 740-YP (Anygen, Gwangju, South Korea, catalog #: 29153, lot #: J240124), 0.1 μM butyzamide (Shionogi, Osaka, Japan), 4 mM L-glutamine (Gibco, Grand Island, New York, catalog #: 25030-149), 10 ng/mL human Flt3-Ligand (PeproTech, Cranbury, New Jersey, catalog number: AF-300-19-100UG, lot #: 0124AF45), and 1 μM UM729 (STEMCELL Technologies, Vancouver, Canada, catalog #: 72332, lot #: 1000124015). Cells were plated at 3–5 $\times$ 10^5 cells per mL per well of a Corning CellBIND surface 24-well plate (Corning Costar, Corning, New York, catalog #3337) with half-medium changes every 2–3 days and expanded as required.

NOG-w41 mouse-derived splenocytes were thawed and maintained in RPMI-1640, 1% bovine serum albumin (BSA) (Wako, Osaka, Japan, catalog # 019-23293, lot # LKH4780), and 1% P/S as the basal medium for maintenance in culture and live-cell imaging experiments. Antigen-specific activation was performed with human CD19/Leu-12 protein (His-Tag), biotinylated (Sino Biological, Beijing, China, catalog #11880-H08H-B), added to the basal medium

at a concentration of 1 μg/mL. Jurkat cells were cultured in RPMI supplemented with 1% P/S as a control medium or RPMI supplemented with 1% P/S and human CD19/Leu-12 protein (His-Tag), biotinylated, 1 μg/mL.

Molecular cloning

The 4-1BB/ζ construct was obtained from Addgene (Addgene, Watertown, Massachusetts, plasmid #194457). 2B4/ζ and CD3ζ constructs were adapted from Addgene plasmids (#157744 and #157743, respectively). For 2B4/ζ and CD3ζ, nucleotide regions 3,738–5,189 and 3,738–4,829, respectively, were PCR amplified (Q5 High-Fidelity DNA Polymerase, New England Biolabs, Ipswich, Massachusetts, #M0491S) from the original plasmids with additional flanking homology. The anti-CD19 scFv sequence was PCR-amplified from nucleotides 3,151–3,960 of the 4-1BB/ζ plasmid. Constructs were cloned into a lentiviral backbone corresponding to the lentiviral backbone vector of the Addgene #194457 plasmid by HiFi assembly (NE Builder HiFi DNA Assembly Master Mix, New England Biolabs, #E2621L), using the added flanking homology regions between the fragments and the lentiviral vector. Sequences were verified by Sanger sequencing post-assembly.

Lentiviral production

Lentivirus was produced in HEK293T cells. HEK293T cells were plated at a density of 4×10^6 cells per 10-cm dish the day prior to transfection. The following day, cells were transfected using the TransIT-PRO Transfection Kit (catalog number # MIR5700, lot # 000000215) according to the manufacturer's instructions. Per 10-cm dish, 10 μL of TRANSIT-PRO Reagent and 5 μL of PRO Boost Reagent, 10 μg of transfer plasmid, 5 μg of psPAX2, and 2.5 μg of VSV-G were diluted and mixed in 1 mL Opti-MEM (Gibco, catalog # 31985-062) and incubated for 15 min at room temperature before addition to the cells. 16 h after transfection, the medium was replaced with fresh DMEM, 10% FBS, and 1% P/S. Viral supernatant was harvested 48 h post-transfection, filtered through a Millex-HP 0.45 μm fast-flow low-binding filter (Merck, catalog # SLHPR33RS), and concentrated by ultracentrifugation at 4,200 × *g* for 16 h at 4°C. Lentiviral pellets were resuspended in PBS and stored on ice for immediate use or aliquoted and stored at −80°C.

PB-T cell transduction

Pre-activated whole PBMCs, as described in the "cell culture" section, were subject to negative T cell isolation (Pan T cell Isolation Kit, Miltenyi Biotec, Bergisch Gladbach, Germany, #130-096-535). The isolated T cells were then re-activated using T cell Activation/Expansion Kit to stimulate proliferation and promote efficient lentiviral transduction. PB-T cells were transduced with 4-1BB/ζ lentivirus at 48 and 72 h after PB-T cell isolation and activation. A half-medium change was conducted 6 h after each transduction.

Human HSPC transduction

Human HSPCs were transduced by adding lentiviral particles directly to cells plated in a CellBIND 24-well plate, 72 h after thawing. After 6 h, a half-medium change was conducted followed by a second round of lentiviral particles. A second half-medium change was conducted the following day, and cells were *ex vivo* expanded for downstream applications.

Murine xenotransplantation and peripheral blood collection

Immunodeficient NOD.Cg-Prkdc^{scid} Il2rg^{tm1Sug} Kit$^{em1(V831M)Jic}$/Jic (W41/W41) (NOG-w41) mice were kindly provided by the Central Institute for Experimental Medicine and Life Science (Kanagawa, Japan). They were sub-lethally irradiated at 1 Gy prior to transplantation. Human HSPCs (between 4×10^5 and 1.52×10^6 cells per mouse), suspended in 200 μL of culture medium, were injected into each mouse via retro-orbital injection. Two independent xenotransplantation experiments were performed as this was a material-generating experiment to obtain CAR-immune cells. Multiple mice were transplanted per condition within each cohort. The first transplant cohort consisted of 4-1BB/ζ (n = 3), 2B4/ζ (n = 3), and CD3ζ (n = 3), and the second transplant cohort consisted of 4-1BB/ζ (n = 5), 2B4/ζ (n = 5), CD3ζ (n = 5). The two transplantation cohorts consisted of commercially sourced, pooled, cord blood-derived CD34+ HSPCs from different manufacturing lots.

Peripheral blood was collected by submandibular bleeding at weeks 4, 8, 12, and 15 post-transplantation to assess human chimerism and differentiation of myeloid and lymphoid immune cells. Red blood cells (RBCs) were lysed using 4.15 g NH_4Cl/500 mL Milli-Q water RBC lysis buffer in a two-step lysis protocol. 1 mL of buffer was added to samples and incubated for 15 min at room temperature, followed by centrifugation at 440 × *g*, 4°C. A second lysis step was performed through addition of 1 mL of RBC lysis buffer and incubation for 10 min at room temperature followed by centrifugation at 440 × *g* at 4°C.

Mice were euthanized at 16 weeks post-transplantation by CO_2 asphyxiation. The first transplant cohort consisted of 4-1BB/ζ (n = 1), 2B4/ζ (n = 2), and CD3ζ (n = 3), and the second transplant cohort consisted of 4-1BB/ζ (n = 5), 2B4/ζ (n = 5), and CD3ζ (n = 3) alive at sacrifice at 16 weeks post-transplantation. Bone marrow cells were isolated from the long bones of the hind legs by flushing with PBS, and spleen cells were isolated by crushing the spleen between sterile microscopy slides. Single-cell suspensions from each mouse were pooled per CAR condition, per transplant cohort and filtered to remove debris. Cells were either analyzed by flow cytometry or cryopreserved at 4–6 × 10^6 cells per cryotube in CellBanker 1 (Takara Bio, #11910). All animal experiments were conducted in accordance with the University of Tokyo guidelines and approved by the Institutional Animal Care and Use Committee of the Institute of Medical Science, The University of Tokyo (approval no: A25IMS0615).

Flow cytometry and sorting

For single-cell fluorescence-activated cell sorting (FACS) of A549-CD19+mKate+ cells, staining with Zombie Aqua Fixable Viability Kit (BioLegend, San Diego, California, # 423101) at a 1:500 dilution for 20 min at 4°C was conducted. Following washing in PBS

supplemented with 2% FBS, cells were stained with anti-CD19 antibody as in Table S3, at dilutions indicated by the manufacturer. FACS was performed using a FACS ARIA III Special Order Research Product (SORP) (BD Biosciences, Franklin Lakes, New Jersey) using a 100-μm nozzle.

A sample of cultured human HSPCs were stained for phenotypic analysis using antibodies against CD34, CD201, lineage markers (Lin), CD90, and CD45RA (Table S3) at dilutions indicated by the manufacturer for 30 min at 4°C. Cells were washed in 1 mL of PBS, and propidium iodide (PI) was added at a final concentration of 0.5 μg/mL prior to acquisition. Flow cytometric analysis was performed using Cytoflex-S (Beckman Coulter, Brea, California) cytometer and FACS ARIA III Special Order Research Product (SORP) (BD Biosciences, Franklin Lakes, New Jersey).

FACS of PB-4-1BB/ζ-T cells was performed on a FACS ARIA III SORP using a 100-μm nozzle. PB-T cells were sorted into CD3+GFP+ (PB-4-1BB/ζ-T cell) and CD3+GFP− (PB-T cell) populations. The sorted PB-4-1BB/ζ-T cells and PB-T cells were subsequently expanded using medium change and passaging schedules indicated in the protocol for the T cell Activation/Expansion Kit for a total of 2 weeks.

FACS of CAR-immune cells isolated from murine tissues was performed on a FACS ARIA III SORP using a 100-μm nozzle. Prior to antibody staining, human Fc receptors were blocked with FcR Blocking Reagent (Miltenyi Biotec, Bergisch Gladbach, Germany, catalog # 130-059-901) for 10 min at 4°C, and mouse Fc receptors were blocked with Purified Rat Anti-Mouse BD FC Block for 5 min at 4°C (BD Biosciences, Franklin Lakes, New Jersey, catalog #553142) at the respective manufacturer-recommended dilutions. Cells were stained with anti-human CD45 (hCD45), anti-murine CD45 (mCD45), anti-human CD3, anti-human CD56, anti-human CD19, and anti-human CD33 (Table S3) using dilutions indicated by the manufacturer for 30 min at 4°C. Cells were washed in 1 mL PBS and filtered, and PI was added at a final concentration of 0.5 μg/mL prior to acquisition. Flow cytometry and sorting was supported by the FACS Core Laboratory, Institute of Medical Sciences, University of Tokyo.

The gating strategy involved the broad exclusion of debris by gating according to FSC-A and SSC-A. Sequential gating was used to isolate single cells followed by live cells. This population was gated into the hCD45+mCD45-GFP+ (hCD45+GFP+) and hCD45+mCD45-GFP− (hCD45+GFP−) populations. GFP fluorescence was observed as a continuum, and GFP+ and GFP− populations were gated based on relative GFP fluorescence intensity within each hCD45+ sample using consistent gating across samples. Within each population, CD3+ T cells were defined as CD3+CD19−CD33−; CD19+ B cells as CD19+CD3−CD33−; CD33+ myeloid cells as CD33+CD3−CD19−, and CD56+ NK cells as CD56+CD3−. Single-stain controls were used to adjust the voltage of each channel during acquisition and compensation parameters. Gates were set using unstained cell controls. A representative gating strategy is demonstrated in Figure S1H.

SDS-PAGE and native PAGE

rCD19 was diluted in distilled water and loaded at 1–2 μg per well, as indicated. For native PAGE, one condition was incubated at 37°C for 24 h prior to analysis, while the remaining samples were prepared fresh. Samples were mixed 1:1 with native sample buffer (Bio-Rad, California, USA, #1610738) and resolved on Any kD Mini-PROTEAN TGX precast gels (Bio-Rad, #4569034) using Tris/glycine running buffer (Bio-Rad, #1610734). Gels were run at 50 V for 20 min followed by 120 V for 40 min. Precision Plus Protein Dual Color Standards (Bio-Rad, #1610374) were used as a molecular weight marker. For non-reducing SDS-PAGE, samples were mixed 1:1 with 2x Laemmli sample buffer (Bio-Rad, #1610737). For reducing SDS-PAGE, samples were mixed 1:1 2x Laemmli sample buffer and 5% β-mercaptoethanol. For both denaturing conditions, samples were heated at 95°C for 5 min and cooled briefly on ice prior to electrophoresis. Gels were run in Tris/glycine/SDS running buffer (Bio-Rad, #1610732) at 50 V for 20 min and 150 V for 30 min. Following electrophoresis, gels were washed three times for 5 min with 200 mL distilled water and stained with 50 mL Bio-Safe Coomassie stain (Bio-Rad, #1610786) for 1 h followed by washing in 200 mL of distilled water for 30 min. Gels were imaged using a Bio-Rad ChemiDoc imaging system with Coomassie blue detection settings.

Western blotting

Protocol was adapted from Kawano et al.[98] Cells were washed and resuspended in a phosphatase inhibitor- (Cell Signaling Technologies #5872) containing lysis buffer (Cell Signaling Technologies #9803) at a volume of 60 μL per 5×10^5 cells. Cells were lysed by repeatedly passing through a 21G × 1/2 needle. Lysates were boiled at 95°C for 5 min and cooled briefly on ice in a 1:1 or 3:1 sample to Laemmli sample buffer using either 2x or 4x Laemmli buffer (Bio-Rad #161-0737, #161-0747) and 5% β-mercaptoethanol (Sigma-Aldrich #M7522), depending on the protein concentration, as measured by lysate absorbance at 280 nm. Lysates were separated by SDS-PAGE at 50 V for 20 min followed by 150 V for 30 min in Tris/glycine/SDS buffer (Bio-Rad #1610732). Electroblotting of the gel onto polyvinylidene fluoride (PVDF) membranes was performed by the Bio-Rad Trans-Blot Turbo. PVDF membranes were blocked in 1x Tris-buffered saline (TBS) (Bio-Rad #1706435), 0.1% Tween 20 (Sigma-Aldrich #P9416), and 5% skim milk (Fujifilm Wako #190-12865) in distilled water, collectively the blocking buffer, for 30 min and washed in distilled water. Primary antibodies were diluted in blocking buffer, and PVDF membranes were stained overnight at 4°C. The following day, PVDF membranes were washed four times in distilled water. Secondary antibodies were diluted in blocking buffer, and PVDF membranes were stained for 1 h at room temperature. PVDF membranes were washed four times in distilled water. Protein bands were visualized using Super Signal West Femto (Thermo Fisher #34096) and stained in the dark for 4 min. Luminescence was then visualized using the Bio-Rad ChemiDoc imaging

system. Membranes were stripped using Western Blot Stripping Buffer (Thermo Fisher #21059) at room temperature for 15 min, followed by a further 10 min in a 1:1 diluted solution.

RNA-seq

For each of the CAR constructs, spleen-isolated cells were subject to *ex vivo* rCD19 antigen-specific stimulation for 24 h. The spleen-isolated cells were then collected and sorted into hCD45+ GFP+CD3+; CD56+; CD19+; CD33+ (CAR-immune cells) and hCD45-+GFP−CD3+; CD56+; CD19+; CD33+ (non-transduced control immune cells). All populations were sorted directly into and lysed in 500 μL TRIzol LS reagent (Thermo Fisher Scientific, Waltham, Massachusetts, catalog #10296028). Total RNA was extracted using the TAKARA SMARTer RNA extraction protocol (SMARTer Stranded Total RNA-Seq Kit, Takara Bio) according to the manufacturer's instructions. RNA quality and quantity were assessed with a NanoDrop 2000 spectrophotometer (Thermo Fisher Scientific). cDNA libraries were prepared using the TAKARA SMARTer Stranded Total RNA-Seq Kit (Takara Bio) and the high-output kit v2 (Illumina, San Diego, California), following the manufacturer's protocol, and sequenced on an Illumina NextSeq 500 sequencer to generate 2 × 36 paired-end reads. RNA extraction, library preparation, sequencing, and genome alignment were performed at Tsukuba i-Laboratory, LLC (Japan). FASTQ files and expression value data were requested for downstream analysis. Sequencing was performed on a single biological replicate per condition ($n = 1$) in line with the exploratory design. Downstream analyses were conducted descriptively without formal statistical inference.

RNA-seq data were analyzed in R (R v.4.5.0 [April 11, 2025]). To explore the dataset, PCA was performed on variance-stabilized count data generated using DESeq2 (v.1.48.2) using the top variable genes. Sample clustering was visualized using the first two principal components. To assess relative gene expression levels, raw count data was also used to generate counts per million- (CPM)-normalized log2 fold-change (log2FC). For pairwise comparisons, genes were ranked according to log2FC between GFP+ (CAR-expressing; CAR+) and GFP− (non-transduced control; CAR−) populations for each cell type expressing a specific CAR protein. This was exported as ranked lists for downstream GSEA. For exploratory gene-level comparisons, CPM-normalized expression values were calculated from raw count data and log2FC calculated per condition. Genes were filtered using a predefined expression threshold of CPM >1 to select genes that are consistently detected above background and exclude lowly expressed genes that are more likely to reflect sampling noise and unreliable fold-change estimates. A predefined effect size threshold of $|\log_2 FC| > 0.5$ was used to select genes that exhibit increased expression compared with non-transduced control immune cells while retaining genes that undergo subtle expression changes.

GSEA and hallmark pathway analysis were conducted using the.rnk files generated from exploratory fold change-based gene ranking, as described above and fgsea (v.1.34.2) with GOBP and hallmark gene set collections from MSigDB v.2025.1 and MSigDB v.2024.1 respectively. Gene-level contributions to GOBP immune cell activation pathways were visualized as a heatmap using leading-edge analysis from GSEA. Leading-edge genes were extracted and ranked according to their contribution to enrichment. The top 20 positive and negative contributors to enrichment for the GOBP B cell or Myeloid Leukocyte Activation pathways were visualized per CAR design separately for B cells and myeloid cells. Positively enriched hallmark pathways in GFP+ versus GFP− cells were visualized using the NES with ggplot2 (v.4.0.1) and pheatmap (V .0.13). The top 20 enriched pathways within an immune cell lineage across CAR designs were selected and used to construct an NES heatmap across CAR designs. The top 10 enriched pathways within a CAR design across immune cell lineages were selected and used to construct an NES heatmap across immune cell lineages within a CAR design. To identify examples of shared and CAR design-specific activation genes, filtered gene lists from exploratory fold change-based comparisons were intersected across CAR designs for each immune cell type and visualized using Venn diagrams. Selected activation-induced genes were defined as genes with an expression threshold of CPM >1 and were either genes uniquely present in the filtered gene list for a single CAR design or genes exhibiting an effect size difference of $|\log 2FC| > 1$ compared with the same gene in the other CAR designs for a given cell type. This more stringent threshold was applied to highlight genes undergoing pronounced changes in expression.

Benchmark reference signatures were derived from publicly available datasets. For PB-CAR-T cells (GEO: GSE190161),[6] raw counts were analyzed with DESeq2 using a paired design, retaining total counts ≥10. For PB-CAR-NK cells (GEO: GSE184552),[38] normalized expression data were used to calculate donor-matched log2FC. Ranked gene lists were generated from log2FC for all analyses. GSEA was performed using fgsea (v.1.34.2) on pre-ranked gene lists. Ranked lists were derived from log2FC from DESeq2 analysis or from comparisons of normalized expression data. Leading-edge genes were extracted by calculating their relative contribution to pathway enrichment based on the ranked statistic. The top 30 genes per comparison were selected for heatmap visualization. Hallmark gene sets (MSigDB v.2024.1) were analyzed using fgsea across all ranked gene lists. The top 20 enriched pathways from the PB references were selected and used to construct an NES heatmap across CAR designs within an immune cell lineage.

InTraSeq

Sorted spleen-isolated human non-transduced control immune cells and CAR-immune cells (4-1BB/ζ-immune cells and 2B4/ζ-immune cells) were multiplexed using 10× Genomics Protocol 3: Cell Multiplexing Oligo Labeling for samples with >80% viability, according to the manufacturer's instructions. Cells were combined and antigen stimulated for 10 min at 37°C. Cells were counted and immediately fixed with ice-cold methanol according to the InTraSeq protocol (Cell Signaling Technology, Danvers, Massachusetts). Library construction was performed using the 10× Genomics Chromium GEM-X Single Cell 3′ Reagent Kit (10× Genomics, Pleasanton, California), following the manufacturer's instructions. Cells were loaded

at a target concentration of approximately 1,000 cells/μL (29 μL cell suspension + 7.6 μL PBS). cDNA and cell-surface protein library purity and quantity were assessed using an Agilent TapeStation. Sequencing was performed on Illumina Novaseq X by Macrogen (Seoul, South Korea). Sequencing was performed on a single biological replicate per condition (*n* = 1) in line with the exploratory design. Downstream analyses were conducted descriptively without formal statistical inference.

Cells were quality-filtered to exclude low-quality or multiplet profiles (<700 detected genes, >7,000 detected genes, >7% mitochondrial reads). Single-cell antibody-derived tag (ADT) protein expression data and transcriptomic data were analyzed using Seurat (v.5.3.1.0) in R. Initial clustering was conducted using the single-cell RNA-seq data only and was performed on the full dataset at 0.4 after which a high-resolution, focused subset was created based on refined clustering of clusters 6, 7, 8, 9, 10, and 13 at a resolution of 0.5. Cluster annotation was based on canonical marker gene expression and the top 30 differentially expressed genes per cluster. ADT data were analyzed from the CLR-normalized assays, and median protein expression values were calculated for GFP+ and GFP− groups within 4-1BB/ζ-immune cells and 2B4/ζ-immune cells. These matrices were visualized using pheatmap with fixed symmetric color scales.

For a more detailed T cell analysis, T cells were subset from the whole dataset based on cluster identity (6, 7, 9, 10) and reprocessed using the RNA assay following log-normalization, identification of variable features, scaling, and dimensionality reduction by PCA and UMAP (resolution, 0.4). Clustering was performed using a shared nearest neighbor graph. For T cell annotation, average expression of curated lineage and functional marker genes was calculated and scaled across clusters and visualized as a heatmap. Cluster proportions across sample groups were calculated and visualized as stacked bar plots following exclusion of clusters 5 and 6. ADT-based signaling analysis was performed after excluding clusters 5, 6, and 7, where median ADT expression per cluster and sample group was calculated and differences between GFP+ and GFP− conditions were visualized as heatmaps. Median ADT expression differences per cluster were calculated for 4-1BB/ζ-T cells and 2B4/ζ-T cells and visualized as heatmaps.

Human reference PBMCs (PBMC 3k, 10× Genomics),[52] CD34− cells from post-natal thymus (GEO: GSM4127994, thymus 1),[56] and CBMCs (GEO: GSE100866, CBMC 8K 13 AB 10X-RNA UMI)[55] were processed using Seurat (v.5.3.1.0) in R. Raw count matrices were filtered to retain high-quality cells (RNA counts >700 and <7,000; mitochondrial content <7%) followed by log-normalization, identification of variable features, scaling and PCA. UMAP was performed using the first 20 principal components, and graph-based clustering was applied (resolution = 0.4). Cluster identities were annotated by canonical marker expression and the top 30 differentially expressed genes per cluster. Cell identities of the combined human non-transduced control immune cells and CAR-immune cells clustered at a resolution of 0.4 (query dataset) were assigned by label transfer using FindTransferAnchors and TransferData. For label transfer, CBMC clusters corresponding to erythroid lineages, CD34+ progenitor cells, and murine 3T3 fibroblast cells, as part of the experimental design reported by Stoeckius et al.,[55] were excluded as these populations were not expected within the query dataset due to FACS of hCD45+ cells in combination with human lineage markers. Label transfer was performed using the PBMC reference dataset and selected annotated CBMC reference clusters across *in vivo* differentiated HSPC-derived immune cells, or the post-natal CD34− thymocyte reference dataset for T cell subset analysis. Labels with a maximum prediction score were used to define high-confidence labels, and a threshold of <0.5 was used to classify cells as "Uncertain."

T cell subsets were extracted from PBMC and query datasets and reprocessed at a resolution of 0.4, using the single-cell RNA-seq expression levels. For comparative analysis, PBMC T cells, query T cells, and thymocyte populations were integrated using CCA-based integration following independent normalization and feature selection, restricting to shared genes. The integrated dataset was scaled, analyzed by PCA, and visualized using UMAP, retaining original cluster annotations. Average expression of manually selected canonical gene sets was calculated and visualized as row-scaled heatmaps using pheatmap.

Live-cell, quantitative phase imaging

The protocol was adapted from Yogo et al.[99] The day prior to imaging, antigen-expressing, tumor A549-CD19−mKate target cells were seeded onto U-bottomed 96-well plates. Co-culture was initiated the following day by FACS of human non-transduced control hCD45+ GFP-CD3+; CD56+; CD33+ and CAR-immune hCD45+GFP+ CD3+; CD56+; CD33+ cells directly into the wells of the U-bottomed 96-well plate. The HSPC-derived immune cells were co-cultured with A549-CD19− mKate target cells at a 1:1 effector to target (E:T) cell ratio and imaged for 48 h using the Livecyte system (Phasefocus, Sheffield, United Kingdom) at 10× magnification. Images were taken at 15-min intervals and analyzed using the Analyze software (Phasefocus) with single-cell segmentation. Effector cells and A549-CD19−mKate target cells were assessed by morphology. A549-CD19−mKate target cell identity was confirmed by reference to the corresponding mKate fluorescence, while binding events were quantified using quantitative phase images. Binding events were defined as direct physical contact between an effector cell and a target cell, identified by visible overlap of their cell boundaries within the phase image. For each well, target cell number, effector cell number, and binding events were quantified from three representative time points, 12:00, 12:15, and 12:30, selected to capture established effector cell-target cell interactions prior to substantial changes in E:T cell ratio. Binding frequency was calculated for each time point (binding events/[effector cell number × target cell number]) to account for variation in both effector cell and target cell abundance in the field of view. The resulting values were averaged across the three time points to generate a single value per well. Wells with no detectable effector cells or no detectable target cells were excluded from analysis.

Luminescence-based *in vitro* cytotoxicity assay

The day prior to the initiation of co-culture, clonal A549-CD19−akaluc target cells were seeded onto U-bottomed 96-well plates at 200 cells per well. The following day, spleen-isolated cells were sorted into hCD45+GFP− and hCD45+GFP+ populations for each CAR design, where 800 effector cells of each population were directly plate-sorted into wells containing the seeded target cells. Control wells remained target cell-only wells, and human immune cells were not sorted into them. After 30 h, luminescence was measured using the CLARIOstar Plus (BMGLabtech, Germany, Ortenberg) immediately after the addition of RPMI, 1% P/S, 1% BSA, and 1.7 μg/mL akalumine. Relative luminescence units from co-culture wells were compared with target cell-only wells seeded at the same density and at the same time as the target cells in the co-culture wells.

Human IL6 *ELISA*

300 spleen-isolated cells were sorted into hCD45+GFP+CD3+ and hCD45+GFP-CD3+ populations for the 4-1BB/ζ, 2B4/ζ, and CD3ζ CARs. The relevant cells were directly plate-sorted into RPMI, 1% P/S, 1% BSA, and 1 μg/mL human CD19/Leu-12 protein (His-Tag), biotinylated (Sino Biological, Beijing, China, catalog #11880-H08H-B). After 48 h of *ex vivo* culture, the supernatant was collected, centrifuged for 5 min at 500 × *g*, collected, and stored at −80°C. Human IL6 sandwich ELISA kit (Proteintech, Rosemont, United States, catalog #KE00139) was used according to manufacturer instructions. Cell supernatant for each sample was diluted 5-fold in sample diluent PT 4 solution. Following addition of tetramethylbenzidine (TMB) solution, plates were protected from light, and color development was observed at 3-min intervals. Once a visible signal was observed at the lowest standard concentration, the reaction was stopped by the addition of stop solution. Immediately after, the plate was briefly shaken to ensure mixing, and absorbance was subsequently measured at 450 nm using the CLARIOstar Plus (BMGLabtech, Germany, Ortenberg). ELISA measurements were performed on a single biological replicate ($n = 1$) and are presented descriptively without formal statistical testing.

Statistical analysis

Statistical analysis was performed in GraphPad Prism (v.10.4.2). Flow cytometric, quantitative phase imaging binding event quantification, *in vitro* cytotoxicity, and IL6 production data are presented as individual values, with the median indicated by a horizontal line. Measures of variance were not displayed owing to the small sample sizes in several experimental groups. Parametric statistical testing was used throughout, assuming normal distribution and homogeneity of variances, given the exploratory nature of the study and limited sample sizes in several comparisons. Formal normality testing was not performed.

Statistical comparisons were performed using a one-way ANOVA with Tukey's multiple comparisons test, two-way ANOVA with Tukey's multiple comparisons test, or two-way ANOVA with Šídák's multiple comparisons test, using a significance threshold of $p < 0.05$. Where one-way ANOVA with Tukey's post hoc test was applied, all pairwise comparisons were performed between individual CAR designs, unless stated otherwise, reflecting the independent xenotransplantation of HSPCs engineered with different CAR constructs. Where two-way ANOVA with Tukey's post hoc test was applied for hCD45+:mCD45+ cell ratios isolated from the spleen and bone marrow upon NOG-w41 sacrifice, all pairwise comparisons were performed to assess human engraftment. Assessment of immune cell distribution in the peripheral blood used two-way ANOVA with Tukey's multiple comparisons test, and all pairwise comparisons were conducted with comparisons visualized between immune cell lineages within a CAR design. Two-way ANOVA with Tukey's multiple comparisons test was used to assess CD69 expression on Jurkat cells and 4-1BB/ζ-Jurkat cells. Multiple comparisons were conducted across all pairwise combinations, and selected comparisons between culture medium condition and cell type were visualized. When comparing the immune cell distribution in the spleen and bone marrow, statistical comparisons were performed between GFP+ and GFP− populations within each CAR design and immune cell type using a two-way ANOVA and Šídák's multiple comparisons test. For *in vitro* cytotoxicity data, two-way ANOVA with Šídák's multiple comparisons test was used to compare hCD45+GFP+ and hCD45+GFP− luminescence values within a CAR design. Further information on the statistical tests applied, the number of biological replicates, and the groups compared are provided in the figure legends. Statistical significance is indicated as $^{*}p < 0.05$, $^{**}p \leq 0.01$, $^{***}p \leq 0.001$, and $^{****}p \leq 0.0001$.

ELISA measurements were performed on a single biological replicate ($n = 1$) and are presented descriptively without formal statistical inference. Sample concentrations were determined by fitting a four-parameter logistic (4PL) sigmoidal standard curve and interpolating from the fitted curve.

Bulk RNA-seq and single-cell InTraSeq dataset analyses were conducted in an exploratory framework using a single biological replicate per condition ($n = 1$) and are, therefore, presented descriptively without formal statistical testing. Results are presented descriptively based on normalized expression values, log2FC ranking, and pathway enrichment analyses.

DATA AND CODE AVAILABILITY

The datasets generated during the current study are available from the corresponding author on reasonable request. The sequencing data have been deposited in the Gene Expression Omnibus under accession number GEO: GSE333486 and are publicly available.

ACKNOWLEDGMENTS

We thank Dr. Maiko Morita for advice and supervision during PB mononuclear cell and PB-T cell culture. We thank Tsukuba i-Laboratory, LLC (Japan), for RNA extraction, library preparation, sequencing, and genome alignment. Flow cytometry and cell sorting were supported by the FACS Core Laboratory, Institute of Medical Science, University of Tokyo. All animal experiments were conducted in accordance with University of Tokyo guidelines and approved by the Institutional Animal Care and Use Committee of the Institute of Medical Science (approval no: A25IMS0615), and human PB samples were obtained and analyzed in accordance with University of Tokyo guidelines and approved by the Ethics Committee (approval no: 2026-18-0616). We thank Shu Iida, Jonathan Feito, and Kevin Langley at Bruker for their technical support with quantitative phase imaging and analysis. This work was financially enabled by the Japan Society for the

Promotion of Science (JSPS; 24K10972 to K.I., 25K10486 to Y.K., 24K19192 to T.Y., 23K14189 to H.J., 20K21613 to T.F., and 24K02478 to S.Y.), the Japan Agency for Medical Research and Development (AMED; 25bm1123084h0001 to T.Y. and 25bm1223011h0003 to S.Y.), the Japan Science and Technology Agency (JST; JPMJAX25L9 to T.F.), and JST SPRING (JPMJSP2108 to A.S.-F).

AUTHOR CONTRIBUTIONS

A.S.-F. and S.Y. conceptualized the study and designed the experiments. A.S.-F performed the experiments and wrote the manuscript. R.I. established the humanized NOG-w41 mouse model and supplied the mice used in this study. S.Y. performed xenotransplantation, peripheral blood collection, and euthanasia. T.K. supervised InTraSeq library preparation, conducted reference genome alignment, and supervised InTraSeq analysis. T.Y. provided vectors. S.Y., Y.K., K.I., T.Y., T.K., and T.F. provided experimental guidance, technical advice, and supervision. S.Y. oversaw the project and supervised. S.Y., T.Y., H.J., Y.K., and T.F. supported manuscript review and editing. All authors contributed to data interpretation and manuscript editing and approved the final version.

DECLARATION OF INTERESTS

The authors declare the following competing financial interest: Celaid Therapeutics Inc.

DECLARATION OF GENERATIVE AI AND AI-ASSISTED TECHNOLOGIES IN THE WRITING PROCESS

During the preparation of this work, the authors used ChatGPT in order to assist with code development, refinement of wording, and proofreading. After using this tool, the authors reviewed and edited the content as needed and take full responsibility for the content of the published article.

SUPPLEMENTAL INFORMATION

Supplemental information can be found online at https://doi.org/10.1016/j.omton.2026.201312.

REFERENCES

1. Wilkinson, A.C., Igarashi, K.J., and Nakauchi, H. (2020). Haematopoietic stem cell self-renewal in vivo and ex vivo. Nat. Rev. Genet. *21*, 541–554. https://doi.org/10.1038/s41576-020-0241-0.

2. Sakurai, M., Ishitsuka, K., Ito, R., Wilkinson, A.C., Kimura, T., Mizutani, E., Nishikii, H., Sudo, K., Becker, H.J., Takemoto, H., et al. (2023). Chemically defined cytokine-free expansion of human haematopoietic stem cells. Nature *615*, 127–133. https://doi.org/10.1038/s41586-023-05739-9.

3. Uchida, N., Nassehi, T., Drysdale, C.M., Gamer, J., Yapundich, M., Demirci, S., Haro-Mora, J.J., Leonard, A., Hsieh, M.M., and Tisdale, J.F. (2019). High-Efficiency Lentiviral Transduction of Human CD34+ Cells in High-Density Culture with Poloxamer and Prostaglandin E2. Mol. Ther. Methods Clin. Dev. *13*, 187–196. https://doi.org/10.1016/j.omtm.2019.01.005.

4. Sakurai, M., Ishitsuka, K., Becker, H.J., and Yamazaki, S. (2024). Ex vivo expansion of human hematopoietic stem cells and clinical applications. Cancer Sci. *115*, 698–705. https://doi.org/10.1111/cas.16066.

5. Sterner, R.C., and Sterner, R.M. (2021). CAR-T cell therapy: current limitations and potential strategies. Blood Cancer J. *11*, 69. https://doi.org/10.1038/s41408-021-00459-7.

6. Salter, A.I., Ivey, R.G., Kennedy, J.J., Voillet, V., Rajan, A., Alderman, E.J., Voytovich, U.J., Lin, C., Sommermeyer, D., Liu, L., et al. (2018). Phosphoproteomic analysis of chimeric antigen receptor signaling reveals kinetic and quantitative differences that affect cell function. Sci. Signal. *11*, eaat6753. https://doi.org/10.1126/scisignal.aat6753.

7. Schmidt, D., Ebrahimabadi, S., Gomes, K.R.D.S., de Moura Aguiar, G., Cariati Tirapelle, M., Nacasaki Silvestre, R., De Azevedo, J.T.C., Tadeu Covas, D., and Picanço-Castro, V. (2022). Engineering CAR-NK cells: how to tune innate killer cells for cancer immunotherapy. Immunother. Adv. *2*, ltac003. https://doi.org/10.1093/immadv/ltac003.

8. Zhong, Y., and Liu, J. (2024). Emerging roles of CAR-NK cell therapies in tumor immunotherapy: current status and future directions. Cell Death Discov. *10*, 318. https://doi.org/10.1038/s41420-024-02077-1.

9. Klichinsky, M., Ruella, M., Shestova, O., Lu, X.M., Best, A., Zeeman, M., Schmierer, M., Gabrusiewicz, K., Anderson, N.R., Petty, N.E., et al. (2020). Human chimeric antigen receptor macrophages for cancer immunotherapy. Nat. Biotechnol. *38*, 947–953. https://doi.org/10.1038/s41587-020-0462-y.

10. Chang, Y., Syahirah, R., Wang, X., Jin, G., Torregrosa-Allen, S., Elzey, B.D., Hummel, S.N., Wang, T., Li, C., Lian, X., et al. (2022). Engineering chimeric antigen receptor neutrophils from human pluripotent stem cells for targeted cancer immunotherapy. Cell Rep. *40*, 111128. https://doi.org/10.1016/j.celrep.2022.111128.

11. Behrens, L.M., Van Egmond, M., and Van Den Berg, T.K. (2023). Neutrophils as immune effector cells in antibody therapy in cancer. Immunol. Rev. *314*, 280–301. https://doi.org/10.1111/imr.13159.

12. De Oliveira, S.N., Ryan, C., Giannoni, F., Hardee, C.L., Tremcinska, I., Katebian, B., Wherley, J., Sahaghian, A., Tu, A., Grogan, T., et al. (2013). Modification of Hematopoietic Stem/Progenitor Cells with CD19-Specific Chimeric Antigen Receptors as a Novel Approach for Cancer Immunotherapy. Hum. Gene Ther. *24*, 824–839. https://doi.org/10.1089/hum.2012.202.

13. Wang, T., Liu, P., Zhang, D., Song, Z., Yu, M., Feng, D., Yu, X., Liu, N., Tang, G., and Yang, J. (2025). Chimeric antigen receptor modified hematopoietic stem cells (CAR-HSCs) arm all immune forces for anti-tumor in mice. Exp. Hematol. Oncol. *14*, 124. https://doi.org/10.1186/s40164-025-00715-7.

14. Pan, K., Farrukh, H., Chittepu, V.C.S.R., Xu, H., Pan, C.X., and Zhu, Z. (2022). CAR race to cancer immunotherapy: from CAR T, CAR NK to CAR macrophage therapy. J. Exp. Clin. Cancer Res. *41*, 119. https://doi.org/10.1186/s13046-022-02327-z.

15. Look, T., Sankowski, R., Bouzereau, M., Fazio, S., Sun, M., Buck, A., Binder, N., Mastall, M., Prisco, F., Seehusen, F., et al. (2025). CAR T cells, CAR NK cells, and CAR macrophages exhibit distinct traits in glioma models but are similarly enhanced when combined with cytokines. Cell Rep. Med. *6*, 101931. https://doi.org/10.1016/j.xcrm.2025.101931.

16. Li, Y., Hermanson, D.L., Moriarity, B.S., and Kaufman, D.S. (2018). Human iPSC-Derived Natural Killer Cells Engineered with Chimeric Antigen Receptors Enhance Anti-tumor Activity. Cell Stem Cell *23*, 181–192.e5. https://doi.org/10.1016/j.stem.2018.06.002.

17. Lei, A., Yu, H., Lu, S., Lu, H., Ding, X., Tan, T., Zhang, H., Zhu, M., Tian, L., Wang, X., et al. (2024). A second-generation M1-polarized CAR macrophage with antitumor efficacy. Nat. Immunol. *25*, 102–116. https://doi.org/10.1038/s41590-023-01687-8.

18. Wu, S.-Y., Tyagi, A., Wu, K., Smith, E.C., Song, Q., Sharma, S., Miller, L.D., Zhang, W., Pan, B.-S., Lin, H.-K., et al. (2026). MyD88-mediated chimaeric antigen receptor macrophages suppress brain metastasis using target-specific phagocytosis. Nat. Biomed. Eng. https://doi.org/10.1038/s41551-026-01613-x.

19. Azevedo, J., Silvestre, R., Covas, D., and Picanço-Castro, V. (2021). NK-SPECIFIC CAR DESIGN IMPROVES NK CYTOTOXICITY AGAINST TUMOR CELLS. Hematol. Transfus. Cell Ther. *43*, S265. https://doi.org/10.1016/j.htct.2021.10.449.

20. Ito, R., Ohno, Y., Mu, Y., Ka, Y., Ito, S., Emi-Sugie, M., Mochizuki, M., Kawai, K., Goto, M., Ogura, T., et al. (2024). Improvement of multilineage hematopoiesis in hematopoietic stem cell-transferred c-kit mutant NOG-EXL humanized mice. Stem Cell Res. Ther. *15*, 182. https://doi.org/10.1186/s13287-024-03799-w.

21. Silva, G., Rodrigues, A.F., Ferreira, S., Matos, C., Eleutério, R.P., Marques, G., Kucheryava, K., Lemos, A.R., Sousa, P.M.F., Castro, R., et al. (2023). Novel scFv against Notch Ligand JAG1 Suitable for Development of Cell Therapies toward JAG1-Positive Tumors. Biomolecules *13*, 459. https://doi.org/10.3390/biom13030459.

22. Cimo, A.-M., Ahmed, Z., McIntyre, B.W., Lewis, D.E., and Ladbury, J.E. (2013). CD25 and CD69 induction by α4β1 outside-in signalling requires TCR early signalling complex proteins. Biochem. J. *454*, 109–121. https://doi.org/10.1042/BJ20130485.

23. Lindner, S.E., Johnson, S.M., Brown, C.E., and Wang, L.D. (2020). Chimeric antigen receptor signaling: Functional consequences and design implications. Sci. Adv. *6*, eaaz3223. https://doi.org/10.1126/sciadv.aaz3223.

24. Chang, Z.L., Lorenzini, M.H., Chen, X., Tran, U., Bangayan, N.J., and Chen, Y.Y. (2018). Rewiring T-cell responses to soluble factors with chimeric antigen receptors. Nat. Chem. Biol. *14*, 317–324. https://doi.org/10.1038/nchembio.2565.

25. Ise, W., Kohyama, M., Schraml, B.U., Zhang, T., Schwer, B., Basu, U., Alt, F.W., Tang, J., Oltz, E.M., Murphy, T.L., and Murphy, K.M. (2011). The transcription

factor BATF controls the global regulators of class-switch recombination in both B cells and T cells. Nat. Immunol. *12*, 536–543. https://doi.org/10.1038/ni.2037.

26. Thomas, M.D., Kremer, C.S., Ravichandran, K.S., Rajewsky, K., and Bender, T.P. (2005). c-Myb Is Critical for B Cell Development and Maintenance of Follicular B Cells. Immunity *23*, 275–286. https://doi.org/10.1016/j.immuni.2005.08.005.

27. Ohtsuka, M., Arase, H., Takeuchi, A., Yamasaki, S., Shiina, R., Suenaga, T., Sakurai, D., Yokosuka, T., Arase, N., Iwashima, M., et al. (2004). NFAM1, an immunoreceptor tyrosine-based activation motif-bearing molecule that regulates B cell development and signaling. Proc. Natl. Acad. Sci. *101*, 8126–8131. https://doi.org/10.1073/pnas.0401119101.

28. Matthes, T., Werner-Favre, C., Tang, H., Zhang, X., Kindler, V., and Zubler, R.H. (1993). Cytokine mRNA expression during an in vitro response of human B lymphocytes: kinetics of B cell tumor necrosis factor alpha, interleukin (IL)6, IL-10, and transforming growth factor beta 1 mRNAs. J. Exp. Med. *178*, 521–528. https://doi.org/10.1084/jem.178.2.521.

29. Zhu, Y.X., Benn, S., Li, Z.H., Wei, E., Masih-Khan, E., Trieu, Y., Bali, M., McGlade, C.J., Claudio, J.O., and Stewart, A.K. (2004). The SH3–SAM Adaptor HACS1 is Up-regulated in B Cell Activation Signaling Cascades. J. Exp. Med. *200*, 737–747. https://doi.org/10.1084/jem.20031816.

30. Kerscher, B., Wilson, G.J., Reid, D.M., Mori, D., Taylor, J.A., Besra, G.S., Yamasaki, S., Willment, J.A., and Brown, G.D. (2016). Mycobacterial receptor, Clec4d (CLECSF8, MCL), is coregulated with Mincle and upregulated on mouse myeloid cells following microbial challenge. Eur. J. Immunol. *46*, 381–389. https://doi.org/10.1002/eji.201545858.

31. Tridandapani, S., Lyden, T.W., Smith, J.L., Carter, J.E., Coggeshall, K.M., and Anderson, C.L. (2000). The Adapter Protein LAT Enhances Fcγ Receptor-mediated Signal Transduction in Myeloid Cells. J. Biol. Chem. *275*, 20480–20487. https://doi.org/10.1074/jbc.M909462199.

32. Vandenbroucke, R.E., Dejonckheere, E., Van Lint, P., Demeestere, D., Van Wonterghem, E., Vanlaere, I., Puimège, L., Van Hauwermeiren, F., De Rycke, R., Mc Guire, C., et al. (2012). Matrix Metalloprotease 8-Dependent Extracellular Matrix Cleavage at the Blood-CSF Barrier Contributes to Lethality during Systemic Inflammatory Diseases. J. Neurosci. *32*, 9805–9816. https://doi.org/10.1523/JNEUROSCI.0967-12.2012.

33. Vanlint, P., and Libert, C. (2006). Matrix metalloproteinase-8: Cleavage can be decisive. Cytokine Growth Factor Rev. *17*, 217–223. https://doi.org/10.1016/j.cytogfr.2006.04.001.

34. Hams, E., Roberts, J., Bermingham, R., Hogan, A.E., O'Shea, D., O'Neill, L., and Fallon, P.G. (2020). Role for Retinoic Acid-Related Orphan Receptor Alpha (RORα) Expressing Macrophages in Diet-Induced Obesity. Front. Immunol. *11*, 1966. https://doi.org/10.3389/fimmu.2020.01966.

35. Wolf, Y., Shemer, A., Polonsky, M., Gross, M., Mildner, A., Yona, S., David, E., Kim, K.-W., Goldmann, T., Amit, I., et al. (2017). Autonomous TNF is critical for in vivo monocyte survival in steady state and inflammation. J. Exp. Med. *214*, 905–917. https://doi.org/10.1084/jem.20160499.

36. Wang, W., Ren, X., Chen, X., Hong, Q., and Cai, G. (2024). Integrin β1–rich extracellular vesicles of kidney recruit Fn1+ macrophages to aggravate ischemia-reperfusion–induced inflammation. JCI Insight *9*, e169885. https://doi.org/10.1172/jci.insight.169885.

37. Ma, W.Y., Wu, Q.L., Wang, S.S., Wang, H.Y., Ye, J.R., Sun, H.S., Feng, Z.P., He, W.B., Chu, S.F., Zhang, Z., and Chen, N.H. (2023). A breakdown of metabolic reprogramming in microglia induced by CKLF1 exacerbates immune tolerance in ischemic stroke. J. Neuroinflammation *20*, 97. https://doi.org/10.1186/s12974-023-02779-w.

38. Christodoulou, I., Ho, W.J., Marple, A., Ravich, J.W., Tam, A., Rahnama, R., Fearnow, A., Rietberg, C., Yanik, S., Solomou, E.E., et al. (2021). Engineering CAR-NK cells to secrete IL-15 sustains their anti-AML functionality but is associated with systemic toxicities. J. Immunother. Cancer *9*, e003894. https://doi.org/10.1136/jitc-2021-003894.

39. Lee, W., Kim, H.S., Baek, S.Y., and Lee, G.R. (2016). Transcription factor IRF8 controls Th1-like regulatory T-cell function. Cell. Mol. Immunol. *13*, 785–794. https://doi.org/10.1038/cmi.2015.72.

40. Ma, X., Xiao, L., Liu, L., Ye, L., Su, P., Bi, E., Wang, Q., Yang, M., Qian, J., and Yi, Q. (2021). CD36-mediated ferroptosis dampens intratumoral CD8+ T cell effector function and impairs their antitumor ability. Cell Metab. *33*, 1001–1012.e5. https://doi.org/10.1016/j.cmet.2021.02.015.

41. Chen, J., Liao, S., Xiao, Z., Pan, Q., Wang, X., Shen, K., Wang, S., Yang, L., Guo, F., Liu, H.F., and Pan, Q. (2022). The development and improvement of immunodeficient mice and humanized immune system mouse models. Front. Immunol. *13*, 1007579. https://doi.org/10.3389/fimmu.2022.1007579.

42. Jorgovanovic, D., Song, M., Wang, L., and Zhang, Y. (2020). Roles of IFN-γ in tumor progression and regression: a review. Biomark. Res. *8*, 49. https://doi.org/10.1186/s40364-020-00228-x.

43. Mazet, J.M., Mahale, J.N., Tong, O., Watson, R.A., Lechuga-Vieco, A.V., Pirgova, G., Lau, V.W.C., Attar, M., Koneva, L.A., Sansom, S.N., et al. (2023). IFNγ signaling in cytotoxic T cells restricts anti-tumor responses by inhibiting the maintenance and diversity of intra-tumoral stem-like T cells. Nat. Commun. *14*, 321. https://doi.org/10.1038/s41467-023-35948-9.

44. Rowbotham, N.J., Hager-Theodorides, A.L., Cebecauer, M., Shah, D.K., Drakopoulou, E., Dyson, J., Outram, S.V., and Crompton, T. (2007). Activation of the Hedgehog signaling pathway in T-lineage cells inhibits TCR repertoire selection in the thymus and peripheral T-cell activation. Blood *109*, 3757–3766. https://doi.org/10.1182/blood-2006-07-037655.

45. Crompton, T., Outram, S.V., and Hager-Theodorides, A.L. (2007). Sonic hedgehog signalling in T-cell development and activation. Nat. Rev. Immunol. *7*, 726–735. https://doi.org/10.1038/nri2151.

46. Gounari, F., and Khazaie, K. (2022). TCF-1: a maverick in T cell development and function. Nat. Immunol. *23*, 671–678. https://doi.org/10.1038/s41590-022-01194-2.

47. Shimizu, H., Mitomo, K., Watanabe, T., Okamoto, S., and Yamamoto, K. (1990). Involvement of a NF-κB-Like Transcription Factor in the Activation of the Interleukin-6 Gene by Inflammatory Lymphokines. Mol. Cell Biol. *10*, 561–568. https://doi.org/10.1128/mcb.10.2.561-568.1990.

48. Takahashi, K., Koga, K., Linge, H.M., Zhang, Y., Lin, X., Metz, C.N., Al-Abed, Y., Ojamaa, K., and Miller, E.J. (2009). Macrophage CD74 contributes to MIF-induced pulmonary inflammation. Respir. Res. *10*, 33. https://doi.org/10.1186/1465-9921-10-33.

49. Ishinaga, H., Jono, H., Lim, J.H., Kweon, S.M., Xu, H., Ha, U.H., Xu, H., Koga, T., Yan, C., Feng, X.H., et al. (2007). TGF-β induces p65 acetylation to enhance bacteria-induced NF-κB activation. EMBO J. *26*, 1150–1162. https://doi.org/10.1038/sj.emboj.7601546.

50. Fenton, S.E., Saleiro, D., and Platanias, L.C. (2021). Type I and II interferons in the anti-tumor immune response. Cancers *13*, 1037. https://doi.org/10.3390/cancers13051037.

51. Ariss, M. (2023). 1513 InTraSeq^{TM}: a multimodal single cell technology for simultaneous measurement of RNA and proteins in single cells to uncover new single cell biology. In Late-Breaking Abstracts (BMJ Publishing Group Ltd), pp. A1737–A1738. https://doi.org/10.1136/jitc-2023-SITC2023.1513.

52. Peela, S. (2025). PBMC 3k dataset. (figshare). https://doi.org/10.6084/M9.FIGSHARE.30610988.

53. Linderman, G.C., Zhao, J., Roulis, M., Bielecki, P., Flavell, R.A., Nadler, B., and Kluger, Y. (2022). Zero-preserving imputation of single-cell RNA-seq data. Nat. Commun. *13*, 192. https://doi.org/10.1038/s41467-021-27729-z.

54. Hu, H., Liu, R., Zhao, C., Lu, Y., Xiong, Y., Chen, L., Jin, J., Ma, Y., Su, J., Yu, Z., et al. (2022). CITEMO^{XMBD} : A flexible single-cell multimodal omics analysis framework to reveal the heterogeneity of immune cells. RNA Biol. *19*, 290–304. https://doi.org/10.1080/15476286.2022.2027151.

55. Stoeckius, M., Hafemeister, C., Stephenson, W., Houck-Loomis, B., Chattopadhyay, P.K., Swerdlow, H., Satija, R., and Smibert, P. (2017). Simultaneous epitope and transcriptome measurement in single cells. Nat. Methods *14*, 865–868. https://doi.org/10.1038/nmeth.4380.

56. Le, J., Ha, V.L., Li, F., Camacho, V., Patel, S., Welner, R.S., and Parekh, C. (2019). Single Cell Transcriptome Mapping of Human Thymopoiesis Reveals a Continuum of Cell States during T-Lineage Specification and Commitment. Blood *134*, 1183. https://doi.org/10.1182/blood-2019-129886.

57. Xu, W., Nair, J.S., Malhotra, A., and Zhang, J.J. (2005). B Cell Antigen Receptor Signaling Enhances IFN-γ-Induced Stat1 Target Gene Expression Through Calcium Mobilization and Activation of Multiple Serine Kinase Pathways. J. Interferon Cytokine Res. *25*, 113–124. https://doi.org/10.1089/jir.2005.25.113.

58. Kovarik, P., Mangold, M., Ramsauer, K., Heidari, H., Steinborn, R., Zotter, A., Levy, D.E., Müller, M., and Decker, T. (2001). Specificity of signaling by STAT1 depends on SH2 and C-terminal domains that regulate Ser727 phosphorylation, differentially affecting specific target gene expression. EMBO J. *20*, 91–100. https://doi.org/10.1093/emboj/20.1.91.

59. Zhang, C.y., Wu, Y.-L., and Boxer, L.M. (2002). Impaired Proliferation and Survival of Activated B Cells in Transgenic Mice That Express a Dominant-negative cAMP-response Element-binding Protein Transcription Factor in B Cells. J. Biol. Chem. *277*, 48359–48365. https://doi.org/10.1074/jbc.M209329200.

60. Schraml, B.U., Hildner, K., Ise, W., Lee, W.-L., Smith, W.A.-E., Solomon, B., Sahota, G., Sim, J., Mukasa, R., Cemerski, S., et al. (2009). The AP-1 transcription factor Batf controls TH17 differentiation. Nature *460*, 405–409. https://doi.org/10.1038/nature08114.

61. Zhao, Y., Ma, C., Chen, C., Li, S., Wang, Y., Yang, T., Stetler, R.A., Bennett, M.V.L., Dixon, C.E., Chen, J., and Shi, Y. (2022). STAT1 Contributes to Microglial/Macrophage Inflammation and Neurological Dysfunction in a Mouse Model of Traumatic Brain Injury. J. Neurosci. *42*, 7466–7481. https://doi.org/10.1523/JNEUROSCI.0682-22.2022.

62. Huang, Z., You, X., Chen, L., Du, Y., Brodeur, K., Jee, H., Wang, Q., Linder, G., Darbousset, R., Cunin, P., et al. (2022). mTORC1 links pathology in experimental models of Still's disease and macrophage activation syndrome. Nat. Commun. *13*, 6915. https://doi.org/10.1038/s41467-022-34480-6.

63. Srour, E.F., Leemhuis, T., Jenski, L., Redmond, R., Fillak, D., and Jansen, J. (2008). Characterization of normal human CD3+ CD5- and γδ T cell receptor positive T lymphocytes. Clin. Exp. Immunol. *80*, 114–121. https://doi.org/10.1111/j.1365-2249.1990.tb06450.x.

64. Ajina, A., and Maher, J. (2018). Strategies to Address Chimeric Antigen Receptor Tonic Signaling. Mol. Cancer Ther. *17*, 1795–1815. https://doi.org/10.1158/1535-7163.MCT-17-1097.

65. Kim, E.H., Sullivan, J.A., Plisch, E.H., Tejera, M.M., Jatzek, A., Choi, K.Y., and Suresh, M. (2012). Signal integration by Akt regulates CD8 T cell effector and memory differentiation. J. Immunol. *188*, 4305–4314. https://doi.org/10.4049/jimmunol.1103568.

66. Kim, E.H., and Suresh, M. (2013). Role of PI3K/Akt signaling in memory CD8 T cell differentiation. Front. Immunol. *4*, 20. https://doi.org/10.3389/fimmu.2013.00020.

67. Clavijo, P.E., and Frauwirth, K.A. (2012). Anergic CD8+ T Lymphocytes Have Impaired NF-κB Activation with Defects in p65 Phosphorylation and Acetylation. J. Immunol. *188*, 1213–1221. https://doi.org/10.4049/jimmunol.1100793.

68. Wan, Y.Y., and DeGregori, J. (2003). The Survival of Antigen-Stimulated T Cells Requires NFκB-Mediated Inhibition of p73 Expression. Immunity *18*, 331–342. https://doi.org/10.1016/S1074-7613(03)00053-0.

69. Li, G., Boucher, J.C., Kotani, H., Park, K., Zhang, Y., Shrestha, B., Wang, X., Guan, L., Beatty, N., Abate-Daga, D., and Davila, M.L. (2018). 4-1BB enhancement of CAR T function requires NF-κB and TRAFs. JCI Insight *3*, e121322. https://doi.org/10.1172/jci.insight.121322.

70. Kaloni, D., Diepstraten, S.T., Strasser, A., and Kelly, G.L. (2023). BCL-2 protein family: attractive targets for cancer therapy. Apoptosis *28*, 20–38. https://doi.org/10.1007/s10495-022-01780-7.

71. Wang, C.-Y., Mayo, M.W., Korneluk, R.G., Goeddel, D.V., and Baldwin, A.S. (1998). NF-κB Antiapoptosis: Induction of TRAF1 and TRAF2 and c-IAP1 and c-IAP2 to Suppress Caspase-8 Activation. Science *281*, 1680–1683. https://doi.org/10.1126/science.281.5383.1680.

72. Catz, S.D., and Johnson, J.L. (2001). Transcriptional regulation of bcl-2 by nuclear factor κB and its significance in prostate cancer. Oncogene *20*, 7342–7351. https://doi.org/10.1038/sj.onc.1204926.

73. Woznicki, J.A., Flood, P., Bustamante-Garrido, M., Stamou, P., Moloney, G., Fanning, A., Zulquernain, S.A., McCarthy, J., Shanahan, F., Melgar, S., and Nally, K. (2020). Human BCL-G regulates secretion of inflammatory chemokines but is dispensable for induction of apoptosis by IFN-γ and TNF-α in intestinal epithelial cells. Cell Death Dis. *11*, 68. https://doi.org/10.1038/s41419-020-2263-0.

74. Grumont, R.J., Rourke, I.J., and Gerondakis, S. (1999). Rel-dependent induction of A1 transcription is required to protect B cells from antigen receptor ligation-induced apoptosis. Genes Dev. *13*, 400–411. https://doi.org/10.1101/gad.13.4.400.

75. Morrissey, M.A., Williamson, A.P., Steinbach, A.M., Roberts, E.W., Kern, N., Headley, M.B., and Vale, R.D. (2018). Chimeric antigen receptors that trigger phagocytosis. eLife *7*, e36688. https://doi.org/10.7554/eLife.36688.

76. Buys, W., and Zambidis, E.T. (2024). Designing Chimeric Antigen Receptors for Myeloid Immune Cells. J. Cancer Biol. Res. *11*, 1144.

77. Stoll, A., Bruns, H., Fuchs, M., Völkl, S., Nimmerjahn, F., Kunz, M., Peipp, M., Mackensen, A., and Mougiakakos, D. (2021). CD137 (4-1BB) stimulation leads to metabolic and functional reprogramming of human monocytes/macrophages enhancing their tumoricidal activity. Leukemia *35*, 3482–3496. https://doi.org/10.1038/s41375-021-01287-1.

78. Geng, T., Yan, Y., Xu, L., Cao, M., Xu, Y., Pu, J., and Yan, J.C. (2020). CD137 signaling induces macrophage M2 polarization in atherosclerosis through STAT6/PPARδ pathway. Cell. Signal. *72*, 109628. https://doi.org/10.1016/j.cellsig.2020.109628.

79. Bartkowiak, T., Jaiswal, A.R., Ager, C.R., Chin, R., Chen, C.-H., Budhani, P., Ai, M., Reilley, M.J., Sebastian, M.M., Hong, D.S., and Curran, M.A. (2018). Activation of 4-1BB on Liver Myeloid Cells Triggers Hepatitis via an Interleukin-27–Dependent Pathway. Clin. Cancer Res. *24*, 1138–1151. https://doi.org/10.1158/1078-0432.CCR-17-1847.

80. Tu, T.H., Kim, C.-S., Goto, T., Kawada, T., Kim, B.-S., and Yu, R. (2012). 4-1BB/4-1BBL Interaction Promotes Obesity-Induced Adipose Inflammation by Triggering Bidirectional Inflammatory Signaling in Adipocytes/Macrophages. Mediators Inflamm. *2012*, 972629. https://doi.org/10.1155/2012/972629.

81. Morva, A., Arroyo, A.B., Andreeva, L., Tapia-Abellán, A., and Luengo-Gil, G. (2025). Unleashing the power of CAR-M therapy in solid tumors: a comprehensive review. Front. Immunol. *16*, 1615760. https://doi.org/10.3389/fimmu.2025.1615760.

82. Du, X., Goh, P.K., Ma, C., Coughlan, E., Greatorex, S., Porter, L.H., Russ, B., Cummins, K.D., Sek, K., Slaney, C.Y., et al. (2025). Targeting PTPN2 enhances human CAR T cell efficacy and the development of long-term memory in mouse xenograft models. Sci. Transl. Med. *17*, eadk0627. https://doi.org/10.1126/scitranslmed.adk0627.

83. Hirschhorn, D., Budhu, S., Kraehenbuehl, L., Gigoux, M., Schröder, D., Chow, A., Ricca, J.M., Gasmi, B., De Henau, O., Mangarin, L.M.B., et al. (2023). T cell immunotherapies engage neutrophils to eliminate tumor antigen escape variants. Cell *186*, 1432–1447.e17. https://doi.org/10.1016/j.cell.2023.03.007.

84. Wang, L., Wang, Y., He, X., Mo, Z., Zhao, M., Liang, X., Hu, K., Wang, K., Yue, Y., Mo, G., et al. (2025). CD70-targeted iPSC-derived CAR-NK cells display potent function against tumors and alloreactive T cells. Cell Rep. Med. *6*, 101889. https://doi.org/10.1016/j.xcrm.2024.101889.

85. Benoist, C., Germain, R.N., and Mathis, D. (2006). A *Plaidoyer* for 'Systems Immunology. Immunol. Rev. *210*, 229–234. https://doi.org/10.1111/j.0105-2896.2006.00374.x.

86. Han, S., Wang, Y., Wang, B., Patel, E., Okada, S., Yang, L.-J., Moreb, J.S., and Chang, L.-J. (2010). Ex vivo development, expansion and in vivoanalysis of a novel lineage of dendritic cells from hematopoietic stem cells. J. Immune Based Ther. Vaccines *8*, 8. https://doi.org/10.1186/1476-8518-8-8.

87. Khodayari, H., Khodayari, S., Ebrahimi, E., Hadjilooei, F., Vesovic, M., Mahmoodzadeh, H., Saric, T., Stücker, W., Van Gool, S., Hescheler, J., and Nayernia, K. (2021). Stem cells-derived natural killer cells for cancer immunotherapy: current protocols, feasibility, and benefits of ex vivo generated natural killer cells in treatment of advanced solid tumors. Cancer Immunol. Immunother. *70*, 3369–3395. https://doi.org/10.1007/s00262-021-02975-8.

88. Li, Y.-R., Dunn, Z.S., Yu, Y., Li, M., Wang, P., and Yang, L. (2023). Advancing cell-based cancer immunotherapy through stem cell engineering. Cell Stem Cell *30*, 592–610. https://doi.org/10.1016/j.stem.2023.02.009.

89. Chan, J.D., Lai, J., Slaney, C.Y., Kallies, A., Beavis, P.A., and Darcy, P.K. (2021). Cellular networks controlling T cell persistence in adoptive cell therapy. Nat. Rev. Immunol. *21*, 769–784. https://doi.org/10.1038/s41577-021-00539-6.

90. Jing, R., Scarfo, I., Najia, M.A., Lummertz da Rocha, E., Han, A., Sanborn, M., Bingham, T., Kubaczka, C., Jha, D.K., Falchetti, M., et al. (2022). EZH1 repression generates mature iPSC-derived CAR T cells with enhanced antitumor activity. Cell Stem Cell *29*, 1181–1196.e6. https://doi.org/10.1016/j.stem.2022.06.014.

91. Hazzalin, C.A., and Mahadevan, L.C. (2002). MAPK-regulated transcription: a continuously variable gene switch? Nat. Rev. Mol. Cell Biol. *3*, 30–40. https://doi.org/10.1038/nrm715.

92. Charrier, S., Ferrand, M., Zerbato, M., Précigout, G., Viornery, A., Bucher-Laurent, S., Benkhelifa-Ziyyat, S., Merten, O.W., Perea, J., and Galy, A. (2011). Quantification of lentiviral vector copy numbers in individual hematopoietic colony-forming cells shows vector dose-dependent effects on the frequency and level of transduction. Gene Ther. *18*, 479–487. https://doi.org/10.1038/gt.2010.163.

93. Coppin, E., Sundarasetty, B.S., Rahmig, S., Blume, J., Verheyden, N.A., Bahlmann, F., Ravens, S., Schubert, U., Schmid, J., Ludwig, S., et al. (2021). Enhanced differentiation of functional human T cells in NSGW41 mice with tissue-specific expression of human interleukin-7. Leukemia *35*, 3561–3567. https://doi.org/10.1038/s41375-021-01259-5.

94. Shultz, L.D., Brehm, M.A., Garcia-Martinez, J.V., and Greiner, D.L. (2012). Humanized mice for immune system investigation: progress, promise and challenges. Nat. Rev. Immunol. *12*, 786–798. https://doi.org/10.1038/nri3311.

95. Watanabe, Y., Takahashi, T., Okajima, A., Shiokawa, M., Ishii, N., Katano, I., Ito, R., Ito, M., Minegishi, M., Minegishi, N., et al. (2009). The analysis of the functions of human B and T cells in humanized NOD/shi-scid/γcnull (NOG) mice (hu-HSC NOG mice). Int. Immunol. *21*, 843–858. https://doi.org/10.1093/intimm/dxp050.

96. Tîrziu, A., Gavriliuc, O.-I., Bojin, M.-F., and Păunescu, V. (2025). Exploring CAR-PBMCs: A Novel Strategy Against EGFR-Positive Tumor Cells. Biomedicines *13*, 264. https://doi.org/10.3390/biomedicines13020264.

97. Zhao, Y., Chokshi, C., Davidson, R., Lin, Y.-C., Santoriello, C., Varshney, D., Pratama, A., Trinidad, D.R., Fox, C., Seregin, A., et al. (2025). 302 *In vivo* HSC engineering with VLPs generates lineage-restricted, multiplexed CAR-M, NK, and T cells to cooperatively mediate robust and durable solid tumor control in pre-clinical models. In Regular and Young Investigator Award Abstracts (BMJ Publishing Group Ltd), pp. A341–A342. https://doi.org/10.1136/jitc-2025-SITC2025.0302.

98. Kawano, Y., Kawano, H., LaMere, M.W., LaMere, E.A., Byun, D.K., McGrath, K.E., Palis, J., Bajaj, J., Liesveld, J.L., Katayama, Y., et al. (2025). IL-1R1 and IL-18 signals regulate mesenchymal stromal cells in an aged murine model of myelodysplastic syndromes. Blood *145*, 1632–1644. https://doi.org/10.1182/blood.2024024818.

99. Yogo, T., Iwamoto, Y., Becker, H.J., Kimura, T., Ishida, R., Sugiyama-Finnis, A., Yokomizo, T., Suda, T., Ota, S., and Yamazaki, S. (2025). Quantitative phase imaging with temporal kinetics predicts hematopoietic stem cell diversity. Nat. Commun. *16*, 6496. https://doi.org/10.1038/s41467-025-61846-3.

OMTON, Volume *34*

Supplemental information

CAR design equips diverse immune cells with unique antigen-specific activation identities

Ayano Sugiyama-Finnis, Takaharu Kimura, Kiyoko Izawa, Yuko Kawano, Takao Yogo, Hyojung Jeon, Tsuyoshi Fukushima, Ryoji Ito, and Satoshi Yamazaki

Table S1: Individual values underlying Figure S1e. Individual values for lineage distributions in peripheral blood at 15 weeks post-transplantation, expressed as the percentage of T cells, NK cells, B cells and myeloid cells among live, single hCD45+GFP+ cells for each CAR design. GFP expression denotes CAR-transduced cells.

Tissue	Condition	T cell (CD3+)					NK cell (CD56+)					B cell (CD19+)					Myeloid cell (CD33+)				
Peripheral blood	4-1BB/ζ	16.9	13.2	7.69	17.7	2.04	0	0	0	0.63	0	63.7	61.8	75.2	57.4	73.5	6.2	6.76	8.97	9.68	5.44
	2B4/ζ	0	35.2	13.2	0	32.1	0	0	0	0	0	20	40.2	57.9	68	53.2	20	11.5	13.2	0	2.63
	CD3ζ	8.75	0	13.6	1.34	0.91	0.19	0	0	0	0	57.2	70	51.3	80.8	80.8	26	12.5	14.3	2.23	4.11

Table S2: Individual values underlying Figure 1f and Figure 1g. Individual values for lineage distributions in spleen and bone marrow shown as the percentage of T cells, NK cells, B cells and myeloid cells among live, single hCD45+GFP+ (CAR+) and hCD45+GFP- (CAR-) cell populations for each CAR design. GFP expression denotes CAR-transduced cells.

Tissue	Condition	T cell (CD3+)			NK cell (CD56+)			B cell (CD19+)			Myeloid cell (CD33+)		
Spleen	4-1BB/ζ (GFP+)	5.83	5.77	2.46	0.16	1.6	0	20.2	24.4	10.7	62.6	53.5	77.9
	2B4/ζ (GFP+)	4.81	1.56	6.64	0.2	0.73	0	10.4	27.2	62.2	79.3	63.5	12.8
	CD3ζ (GFP+)	0.33	0.84	1.31	0.26	1.96	0.44	17.8	4.76	65.6	75.4	88.5	8.18
	4-1BB/ζ control (GFP-)	13.7	14.3	12.7	0.27	0.85	0.23	71	73.7	73	5.59	2.47	6.8
	2B4/ζ control (GFP-)	8.05	3.12	9.34	0.18	0.17	0.24	75.6	87.8	74	5.59	2.08	0.49
	CD3ζ control (GFP-)	1.39	10.4	1.51	0.26	0.85	0.34	84.8	58.4	73.6	3.9	17.2	0.56
Bone marrow	4-1BB/ζ (GFP+)	0.3	0.77	0.091	0.044	0.26	0.091	0.48	1.54	0.37	93.5	81	96.3
	2B4/ζ (GFP+)	0.34	0.39	0.27	0.11	0.39	0.14	0.76	2.71	1.08	91.1	87.2	92
	CD3ζ (GFP+)	0	0	0	0	0	0.33	1.13	1.71	0.31	94.6	85.5	93.1
	4-1BB/ζ control (GFP-)	0.66	0.97	1.14	0.043	0.17	0.071	58.1	79.9	57.2	30.6	11.5	33.9
	2B4/ζ control (GFP-)	0.93	0.92	0.5	0.04	0.038	0.089	56.4	66.6	60.6	31.4	26	29
	CD3ζ control (GFP-)	0.083	0	0.34	0	0	0.16	72	82.8	24.1	19.1	11.2	62.6

Table S3: Antibodies used for flow cytometry and western blotting

Target	Application	Isotype or source	Conjugate	Clone	Manufacturer	Catalogue number
CD34	Flow cytometry	Mouse IgG1 κ	APC	581	Biolegend	343510
CD201	Flow cytometry	Rat IgG1 κ	PE	RCR-401	Biolegend	351940
Lin antibody cocktail	Flow cytometry	Mouse IgG1 κ, Mouse IgG2b κ	BV421	UCHT1, HCD14, 3G8, HIB19, 2H7, HCD56	Biolegend	348805
CD45RA	Flow cytometry	Mouse IgG2b κ	BV510	HI100	Biolegend	304142
CD90	Flow cytometry	Mouse IgG1 κ	APC-cy7	5E10	Biolegend	328132
CD45	Flow cytometry	Mouse IgG1 κ	APC-cy7	HI30	Biolegend	304014
Mouse CD45	Flow cytometry	Rat IgG2b κ	e450	30-F11	eBioscience	48-0451-82
CD3	Flow cytometry	Mouse IgG2a κ	PE	OKT3	eBioscience	12-0037-42
CD56	Flow cytometry	Mouse IgG1 κ	BV510	HCD56	Biolegend	318340
CD19	Flow cytometry	Mouse IgG1 κ	APC	HIB19	eBioscience	17-0199-42
CD33	Flow cytometry	Mouse IgG1 κ	PE-cy7	WM-53	eBioscience	25-0338-42
CD69	Flow cytometry	Mouse IgG1 κ	BV650	FN50	Biolegend	310933
phospho-NF-κB	Western blotting	Rabbit	-	-	Cell Signalling Technologies	3033
phospho-Akt	Western blotting	Rabbit	-	-	Cell Signalling Technologies	9271
Anti-rabbit IgG, horseradish peroxidase linked	Western blotting	Goat	Horseradish peroxidase linked	-	Cell Signalling Technologies	7074

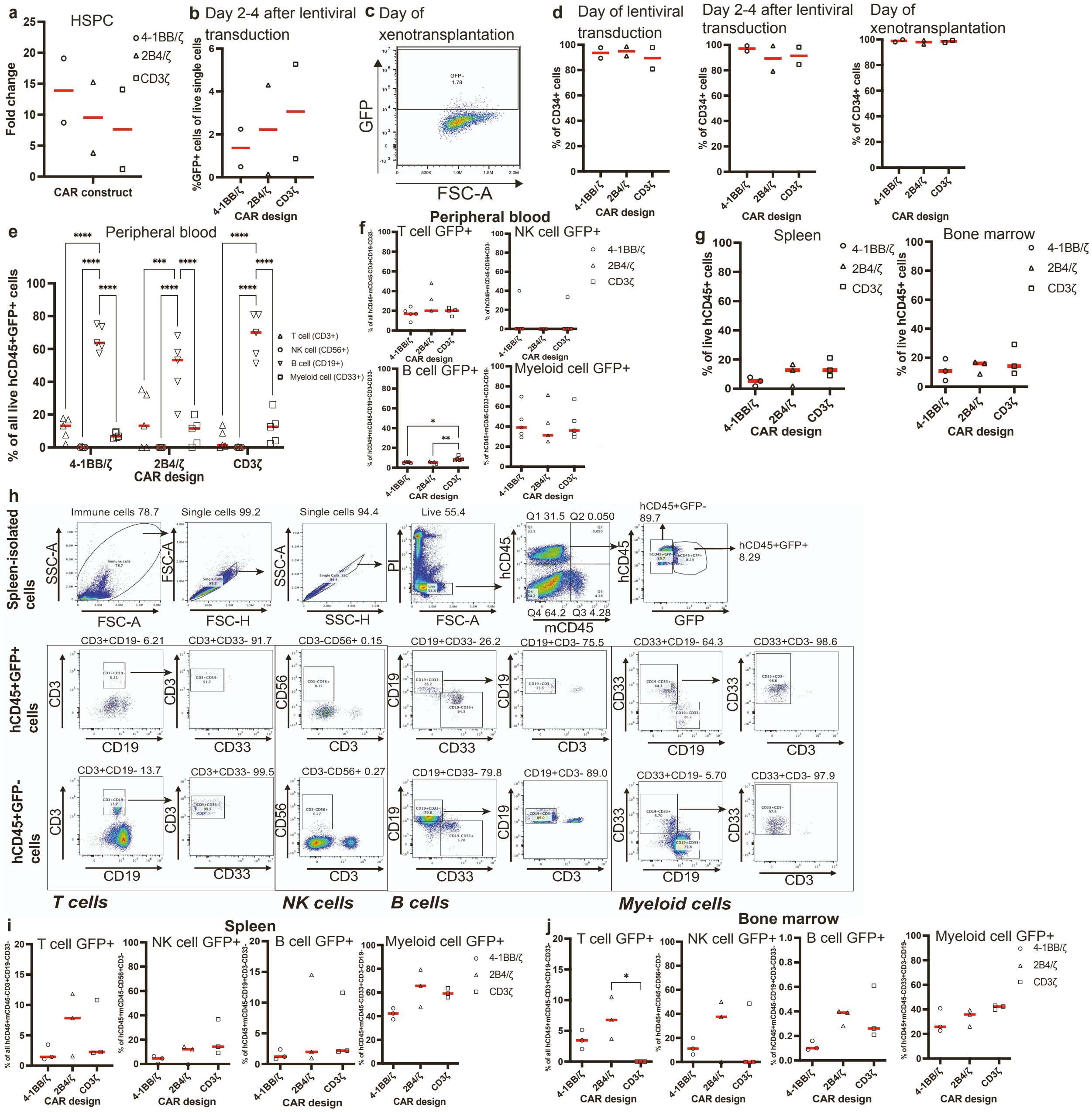

Figure S1: Development of HSPC-derived CAR-immune cells **a** Fold change in live cell number at day 14 compared with day 0 of HSPC expansion culture across the CAR designs. **b** Bar graph showing the percentage of GFP+ cells of live, single CD34+ cells across CAR designs at days 2-4 after lentiviral transduction. **c** Representative flow cytometry plot on the day of xenotransplantation showing GFP+ cells gated on live, single cells. **d** Percentage of CD34+ cells gated on live, single cells across CAR designs on the day of lentiviral transduction (left), 2-4 days after transduction (middle) and on the day of xenotransplantation (right). **e** Immune cell composition in peripheral blood at 15 weeks post-transplantation across CAR designs showing the proportion of T (CD3+), NK (CD56+), B (CD19+) and myeloid cells (CD33+) gated on live, single hCD45+GFP+ cells. **f** Percentage of T (CD3+)(top left), NK (CD56+)(top right), B (CD19+)(*p=0.0209)(bottom left) and myeloid cells (CD33+)(bottom right) in the peripheral blood that are hCD45+GFP+ across CAR designs. **g** Percentage of GFP+ cells out of the live, hCD45+ single cells in the spleen (left) and bone marrow (right) across CAR designs. **h** Representative gating strategy for spleen-isolated cells including gating of immune, single and live cells followed by sequential separation of hCD45+mCD45-GFP+ and hCD45+mCD45-GFP- populations (first row), isolation of T, NK, B and myeloid cells within the hCD45+mCD45-GFP+ population (second row) and the corresponding gating strategy within the hCD45+mCD45-GFP- population (third row). **i** Percentage of T (CD3+)(far left), NK (CD56+)(centre left), B (CD19+)(centre right) and myeloid cells (CD33+)(far right) that are hCD45+GFP+ across CAR designs in the spleen. **j** Percentage of T (CD3+)(*p=0.0175)(far left), NK (CD56+)(centre left), B (CD19+)(centre right) and myeloid cells (CD33+)(far right) that are hCD45+GFP+ across CAR designs in the bone marrow. **a, b, d, e, f, g, i, j** represent individual values with red crossbars indicating the median. No symmetric measure of variance is displayed. **a, b, d** n=2; **e, f** n=5**; g, i, j** n=3. **e,** two-way ANOVA with Tukey's post-hoc test (all pairwise comparisons; selected comparisons shown between cell lineages within each CAR design) **f, g, i, j** one-way ANOVA with Tukey's post-hoc test (all pairwise comparisons across CAR designs), *p < 0.05, **p ≤ 0.01, ***p ≤ 0.001, ****p ≤ 0.0001

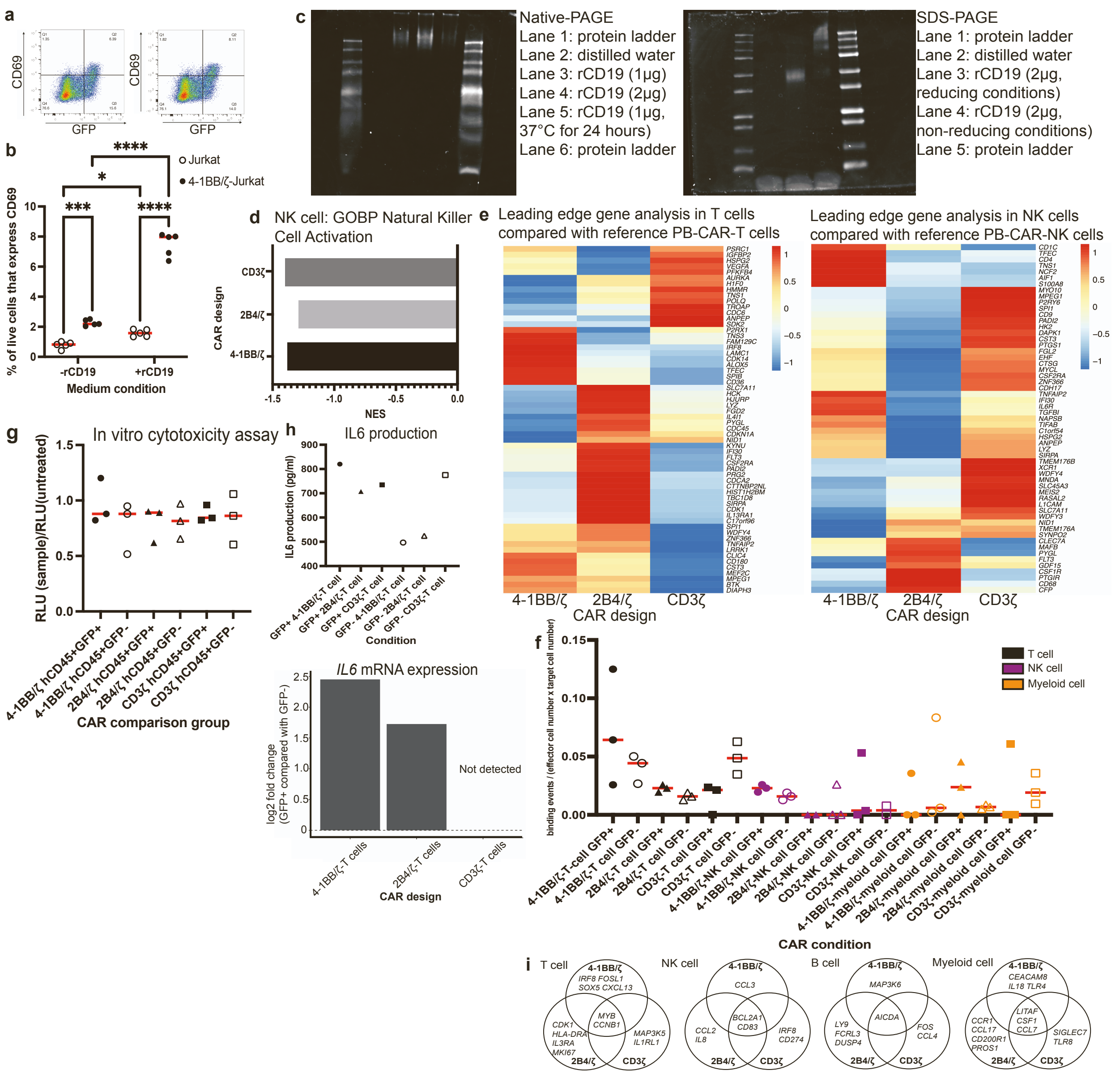

Figure S2: Validation of antigen-specific activation and functional characterisation of HSPC-derived CAR-immune cells. **a** Representative flow cytometry plots of Jurkat (GFP-) and 4-1BB/ζ-Jurkat cells (GFP+) cultured in control medium (left) or rCD19-supplemented medium (right) gated on live, single Jurkat cells. **b** Percentage of CD69+ cells gated on live single cells, for Jurkat (GFP-) and 4-1BB/ζ-Jurkat cells (GFP+) cultured in control or rCD19-supplemented medium (*p=0.0468). **c** Native-PAGE and SDS-PAGE gels stained with Coomassie Blue (Bio-Safe) with lane annotations indicating amounts of rCD19 protein and condition per lane. **d** Bar graph showing NES from GSEA for the GOBP Natural Killer Cell Activation pathway in NK cells across CAR designs following antigen-specific activation. **e** Leading-edge gene contribution heatmaps from GSEA for T cells using reference activated PB-CAR-T cells (left) and for NK cells using reference activated PB-CAR-NK (right) cells across the different CAR designs. The top 30 contributing genes were selected, combined across designs and row-scaled to visualise relative contributions across CAR designs. **f** Quantification of effector cell-target cell binding events from quantitative phase imaging data. Binding events are normalised to effector and target cell number [binding event / (effector cell number x target cell number)]. Wells lacking detectable effector cells or target cells were excluded from analysis. Data are shown descriptively due to the limited number of evaluable wells in certain conditions. **g** In vitro cytotoxicity assay showing relative luminescence units (RLU) normalised to target cell-only wells for each condition across CAR designs. GFP+ indicates CAR+ cells; GFP- indicates non-transduced control cells. **h** IL6 production from CAR-T cells (GFP+) compared with non-transduced control T cells (GFP-) across CAR designs measured by ELISA. Cytokine concentrations are presented as pg/ml (top). Bar graphs showing the log2FC of *IL6* mRNA expression in T cells comparing GFP+ and GFP- cells across CAR designs after 24 hours of antigen-specific activation. *IL6* expression was not detected in CD3ζ-T cells (bottom). **i** Venn diagrams illustrating candidate upregulated genes following antigen-specific activation in T cells (far left), NK cells (centre left), B cells (centre right) and myeloid cells (far right) with each CAR design represented as a separate set. Genes satisfying expression and effect size thresholds (CPM > 1 and |log2FC| > 1 between CAR designs) were included. **b, f, g** represent individual values with median values indicated by red crossbars **b** n=5, two-way ANOVA with Tukey's post-hoc test (all pairwise comparisons; selected comparisons between culture medium and cell type shown). **f** Individual points represent independent wells, n=2/3 due to well exclusion **g** n=3, two-way ANOVA with Šídák's multiple comparisons test (comparisons restricted to GFP+ (CAR+) and GFP- (CAR-) populations within each CAR design) **h** ELISA n=1. **d, e, h, i** RNA-sequencing n=1. *p < 0.05, **p ≤ 0.01, ***p ≤ 0.001, ****p ≤ 0.0001

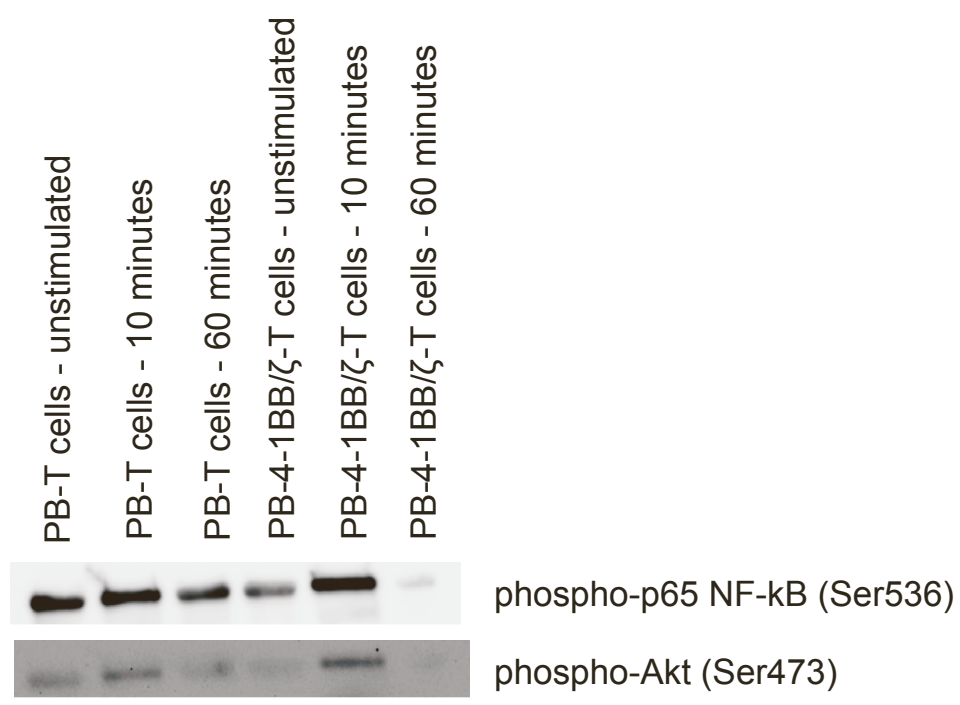

Figure S3: Validation of 10-minute antigen-stimulation. Western blot of peripheral blood (PB)-T cells and PB-4-1BB/ζ-T cells, assessing phosphorylation of p65 NF-kB (Ser536) and Akt (Ser473) in PB-T cells and PB-4-1BB/ζ-T cells that are unstimulated, have undergone 10 minutes of antigen-specific activation or 60 minutes of antigen-specific activation, n=1 samples.

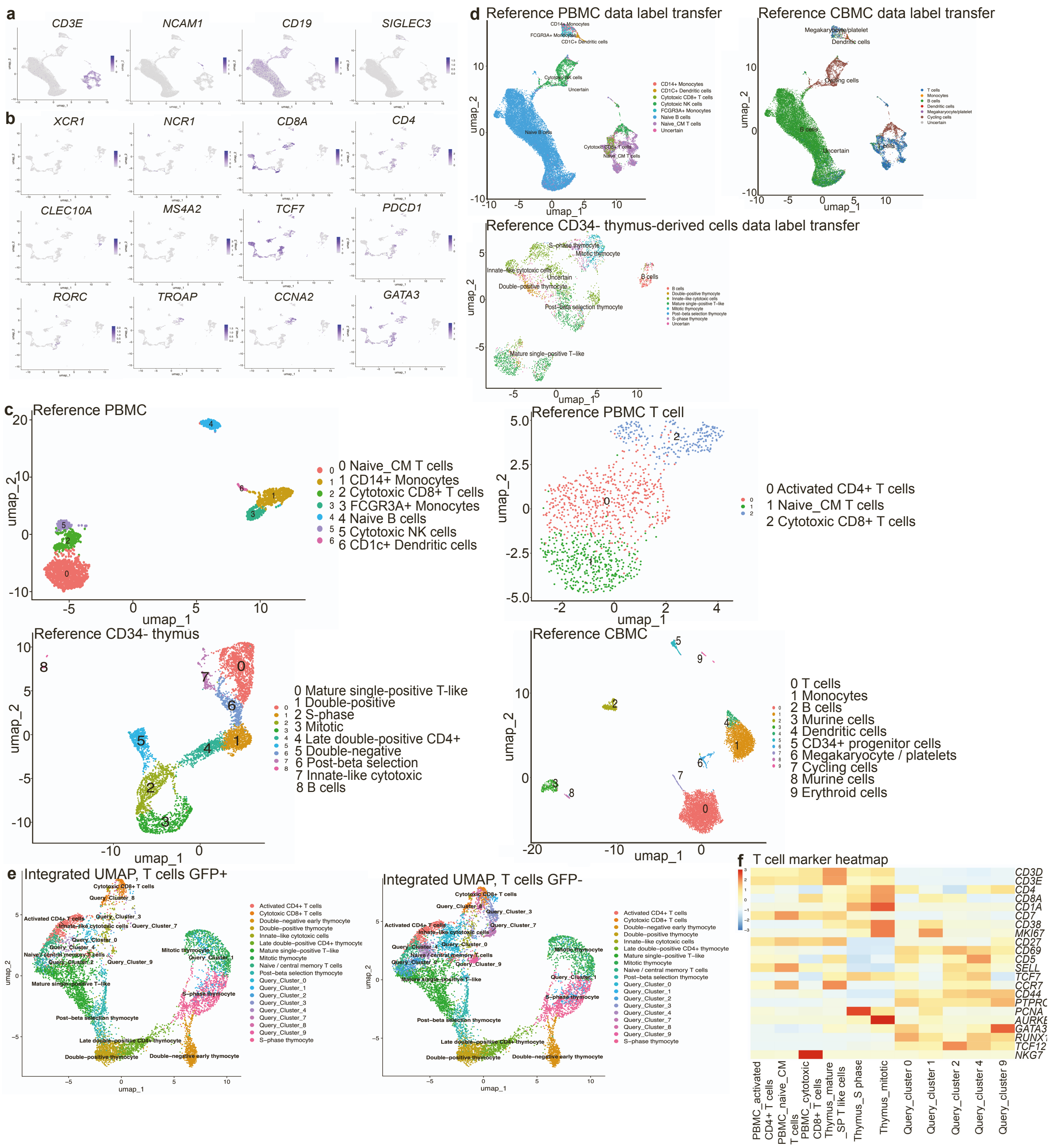
a
CD3E
NCAM1
CD19
SIGLEC3
b
XCR1
NCR1
CD8A
CD4
CLEC10A
MS4A2
TCF7
PDCD1
RORC
TROAP
CCNA2
GATA3
c
Reference PBMC
0 Naive_CM T cells
1 CD14+ Monocytes
2 Cytotoxic CD8+ T cells
3 FCGR3A+ Monocytes
4 Naive B cells
5 Cytotoxic NK cells
6 CD1c+ Dendritic cells
Reference PBMC T cell
0 Activated CD4+ T cells
1 Naive_CM T cells
2 Cytotoxic CD8+ T cells
Reference CD34- thymus
0 Mature single-positive T-like
1 Double-positive
2 S-phase
3 Mitotic
4 Late double-positive CD4+
5 Double-negative
6 Post-beta selection
7 Innate-like cytotoxic
8 B cells
Reference CBMC
0 T cells
1 Monocytes
2 B cells
3 Murine cells
4 Dendritic cells
5 CD34+ progenitor cells
6 Megakaryocyte / platelets
7 Cycling cells
8 Murine cells
9 Erythroid cells
d
Reference PBMC data label transfer
Reference CBMC data label transfer
Reference CD34- thymus-derived cells data label transfer
e
Integrated UMAP, T cells GFP+
Integrated UMAP, T cells GFP-
f
T cell marker heatmap
umap_1
umap_2

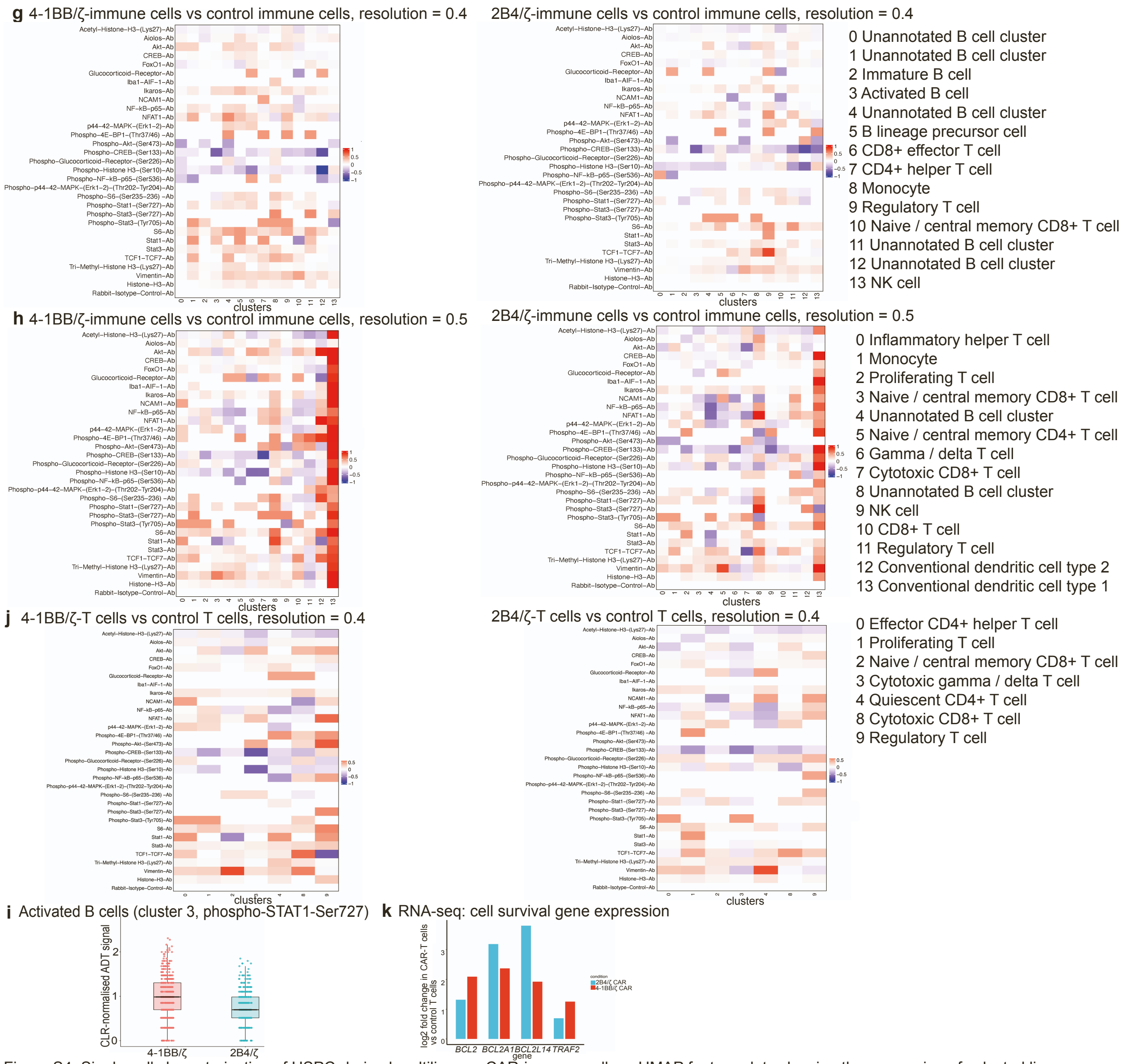

Figure S4: Single-cell characterisation of HSPC-derived multilineage CAR-immune cells. **a** UMAP feature plots showing the expression of selected lineage marker genes across pooled spleen-isolated HSPC-derived immune cells (resolution, 0.4). **b** UMAP feature plots of additional marker gene expression across re-clustered subsets (clusters 6, 7, 8, 9, 10, 13 from **a**; resolution, 0.5). **c** UMAP feature plots of reference PBMC (top left), subset T cells from reference PBMC (top right), reference thymus CD34- cells (bottom left) and reference CBMC (bottom right) processed independently using identical QC filtering and log-normalisation as HSPC-derived CAR-immune cells from **a**. **d** UMAP of pooled HSPC-derived immune cells (from **a**) coloured by predicted cell identity from label transfer using reference PBMC (top left), reference CBMC (top right) and HSPC-derived T cells (clusters 6, 7, 9, 10 from **a**; resolution, 0.4) coloured by predicted cell identity from label transfer using reference thymus CD34- cell (bottom left) datasets. Predicted identities were filtered by prediction score (threshold ≥ 0.5), with low-confidence assignments labelled as "Uncertain". **e** Three-way integrated UMAP of reference PBMC, thymus CD34- and HSPC-derived T cells (GFP+)(left) and HSPC-derived T cells (GFP-)(right) generated using shared gene features and CCA-based integration. Original cluster labels were maintained. **f** Heatmap of selected marker gene expression (rows) across selected reference PBMC, thymus CD34- and HSPC-derived T cell clusters (columns). Values represent row-scaled average single-cell RNA expression. Columns are not hierarchically clustered. **g** Heatmaps of CLR-normalised ADT signalling features with median differences between 4-1BB/ζ-immune cells (GFP+) vs non-transduced control immune cells (GFP-)(left) and 2B4/ζ-immune cells (GFP+) vs non-transduced control immune cells (GFP-)(right) across all HSPC-derived immune cells from **a**, resolved at 0.4. **h** Heatmaps of CLR-normalised ADT signalling features with median differences between 4-1BB/ζ-immune cells (GFP+) vs non-transduced control immune cells (GFP-)(left) and 2B4/ζ-immune cells (GFP+) vs non-transduced control immune cells (GFP-)(right)(clusters 6, 7, 8, 9, 10, 13 from **a**; resolution, 0.5). **i** Boxplots overlaid with individual cells, showing CLR-normalised ADT expression of pSTAT1-(Ser727) for cluster 3 (from **a**, resolution, 0.4). **j** Heatmaps of CLR-normalised ADT signalling features with median differences between 4-1BB/ζ-T cells (GFP+) vs non-transduced control T cells (GFP-)(left) and 2B4/ζ-T cells (GFP+) vs non-transduced control T cells (GFP-)(right)(clusters 6, 7, 9, 10 from **a**; resolution, 0.4). **k** Bar graph showing log2FC expression of selected survival-associated genes from RNA-seq analysis in 4-1BB/ζ-, 2B4/ζ-T cells (GFP+) compared with respective non-transduced control T cells (GFP-) after 24-hour activation. **g**, **h**, **j** Only clusters present in both conditions are shown. Rows represent signalling proteins or post-translational modifications and columns represent clusters. GFP+ denotes CAR+, CAR-expressing cells; GFP- denotes non-transduced control cells. RNA-seq and InTraSeq samples: n=1 biological replicate. Abbreviations: CM, central memory

Movie S1: Quantitative phase imaging of HSPC-derived 4-1BB/ζ-T cells (GFP+) co-cultured with A549-CD19-mKate target cells. Phase contrast videos of a single field of view of HSPC-derived 4-1BB/ζ-T cells (GFP+) co-cultured with A549-CD19-mKate target cells at a 1:1 E:T ratio and imaged at 10x magnification at 15-minute intervals for 48 hours. Scale bar, 200μm, related to Figure 2.

Movie S2: Quantitative phase imaging of HSPC-derived 4-1BB/ζ-NK cells (GFP+) co-cultured with A549-CD19-mKate target cells. Phase contrast videos of a single field of view of HSPC-derived 4-1BB/ζ-NK cells (GFP+) co-cultured with A549-CD19-mKate target cells at a 1:1 E:T ratio and imaged at 10x magnification at 15-minute intervals for 48 hours. Scale bar, 200μm, related to Figure 2.

Movie S3: Quantitative phase imaging of HSPC-derived 4-1BB/ζ-myeloid cells (GFP+) co-cultured with A549-CD19-mKate target cells. Phase contrast videos of a single field of view of HSPC-derived 4-1BB/ζ-myeloid cells (GFP+) co-cultured with A549-CD19-mKate target cells at a 1:1 E:T ratio and imaged at 10x magnification at 15-minute intervals for 48 hours. Scale bar, 200μm, related to Figure 2.
